# Supplementary material for: Stochastic biological system-of-systems modelling for iPSC culture
Source: Commun Biol. 2024 Jan 8;7:39. doi: 10.1038/s42003-023-05653-w (PMC10774284; doi:10.1038/s42003-023-05653-w)
Supplement: Supplementary file 2 — Supplementary Information [file 42003_2023_5653_MOESM2_ESM.pdf]

Supplementary Methods

Proposition 1 *Under the homogeneous population condition, the expected metabolite concentrations are*

$$\frac{d\mathbb{E}[\boldsymbol{u}(t,X)|X]}{dt} = N\frac{d\mathbb{E}[\boldsymbol{\Lambda}(t)]}{dt}X \tag{S.1}$$

where $\boldsymbol{\Lambda}(t) = \big(\Lambda_1(t), \Lambda_2(t), \dots, \Lambda_k(t)\big)$.

Proof. Taking the difference of metabolite concentration (equation (S.1)) between time $\mathrm{t}+\Delta\mathrm{t}$ and t gives

$$\Delta\boldsymbol{u}(t,X) = \boldsymbol{u}(t+\Delta t, X) - \boldsymbol{u}(t,X) = \sum_{j=1}^{k} Y\left(X\int_t^{t+\Delta t} v_j\big(\widetilde{\boldsymbol{u}}_b(\tau)\big)d\tau\right)\big(\boldsymbol{\eta}'_j - \boldsymbol{\eta}_j\big).$$

Given the cell density X at time t, taking expectation of the equation above gives

$$\begin{aligned}
\mathbb{E}[\boldsymbol{u}(t+\Delta t, X)|X] - \mathbb{E}[\boldsymbol{u}(t,X)|X] &= \sum_{j=1}^{k} \mathbb{E}\left[Y\left(X\int_t^{t+\Delta t} v_j\big(\widetilde{\boldsymbol{u}}_b(\tau)\big)d\tau\right)|X\right]\big(\boldsymbol{\eta}'_j - \boldsymbol{\eta}_j\big) \\
&= \sum_{j=1}^{k} X\left(\mathbb{E}\left[Y\left(\Lambda_j(t+\Delta t)\right)\right] - \mathbb{E}\left[Y\left(\Lambda_j(t)\right)\right]\right)\big(\boldsymbol{\eta}'_j - \boldsymbol{\eta}_j\big) \\
&= \sum_{j=1}^{k} X\big(\mathbb{E}[\Lambda_j(t+\Delta t)] - \mathbb{E}[\Lambda_j(t)]\big)\big(\boldsymbol{\eta}'_j - \boldsymbol{\eta}_j\big).
\end{aligned}$$

By dividing Δt and taking limit from both sides, the derivative of expected metabolite concentrations is

$$\begin{aligned}
\frac{d\mathbb{E}[\boldsymbol{u}(t,X)|X]}{dt} &= \lim_{\Delta t\to 0}\frac{\mathbb{E}[\boldsymbol{u}(t+\Delta t, X)|X] - \mathbb{E}[\boldsymbol{u}(t,X)|X]}{\Delta t} = \sum_{j=1}^{k}\frac{\mathbb{E}[\Lambda_j(t+\Delta t)] - \mathbb{E}[\Lambda_j(t)]}{\Delta t}\big(\boldsymbol{\eta}'_j - \boldsymbol{\eta}_j\big)X \\
&= N\frac{d\mathbb{E}[\boldsymbol{\Lambda}(t)]}{dt}X
\end{aligned}$$

which completes the proof.

A constant flux rate $v_j\big(\widetilde{\boldsymbol{u}}_b(t)\big) = v_j$ is often assumed in MFA and EMU method, which indicates a special case of Proposition 1. Notice that $\frac{d\mathbb{E}[\boldsymbol{u}(t,X)|X]}{dt} = N\boldsymbol{v}X$ due to the fact $\boldsymbol{\Lambda}(t) = \int_0^t \boldsymbol{v}dx = \boldsymbol{v}t$. For the differential equation based metabolic network model, we have deterministic metabolite concentrations $\boldsymbol{u}$

and flux rates $\boldsymbol{v}$. Under the steady state, we have the commonly used dynamic metabolic flux analysis model, i.e.,

$$\frac{d\boldsymbol{u}}{dt} = N\boldsymbol{v}X \qquad (S.2)$$

Therefore, by comparing the equation $(S.1)$ with the deterministic model $(S.2)$, we can interpret the classical PDE/ODE-based dynamic metabolic flux analysis model as a special case of the queueing network with mean metabolite concentrations and mean flux rates, i.e., ignoring cell-to-cell variation, stochastic nature of living cells, metabolic and spatial heterogeneity.

Supplementary Figures and Tables

Supplementary Fig. 1. Metabolic network for iPSC used for creating intracellular SMN model (adapted from Wang et al.[30])

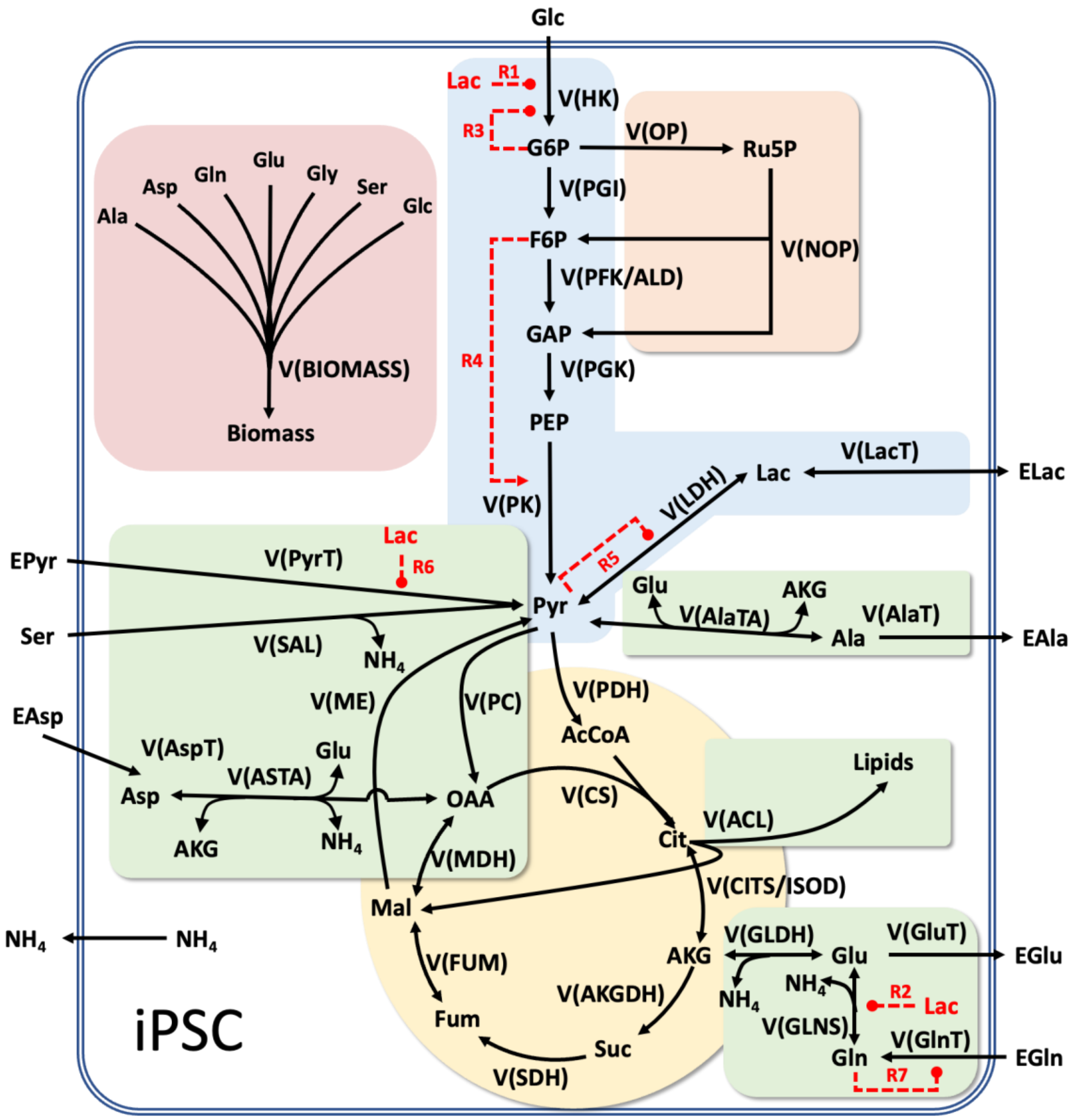

Supplementary Fig. 2. Steady-state metabolite concentration profiles for iPSC aggregates of different sizes. Aggregates sizes shown are 60 μm to 600 μm in radius, where the concentration represent the extracellular conditions within the aggregate. All diffusion effects, porosity $\varepsilon = 0.27$ and tortuosity $\tau = 1.5$ and initial intracellular metabolite concentrations were the same for all aggregate sizes.

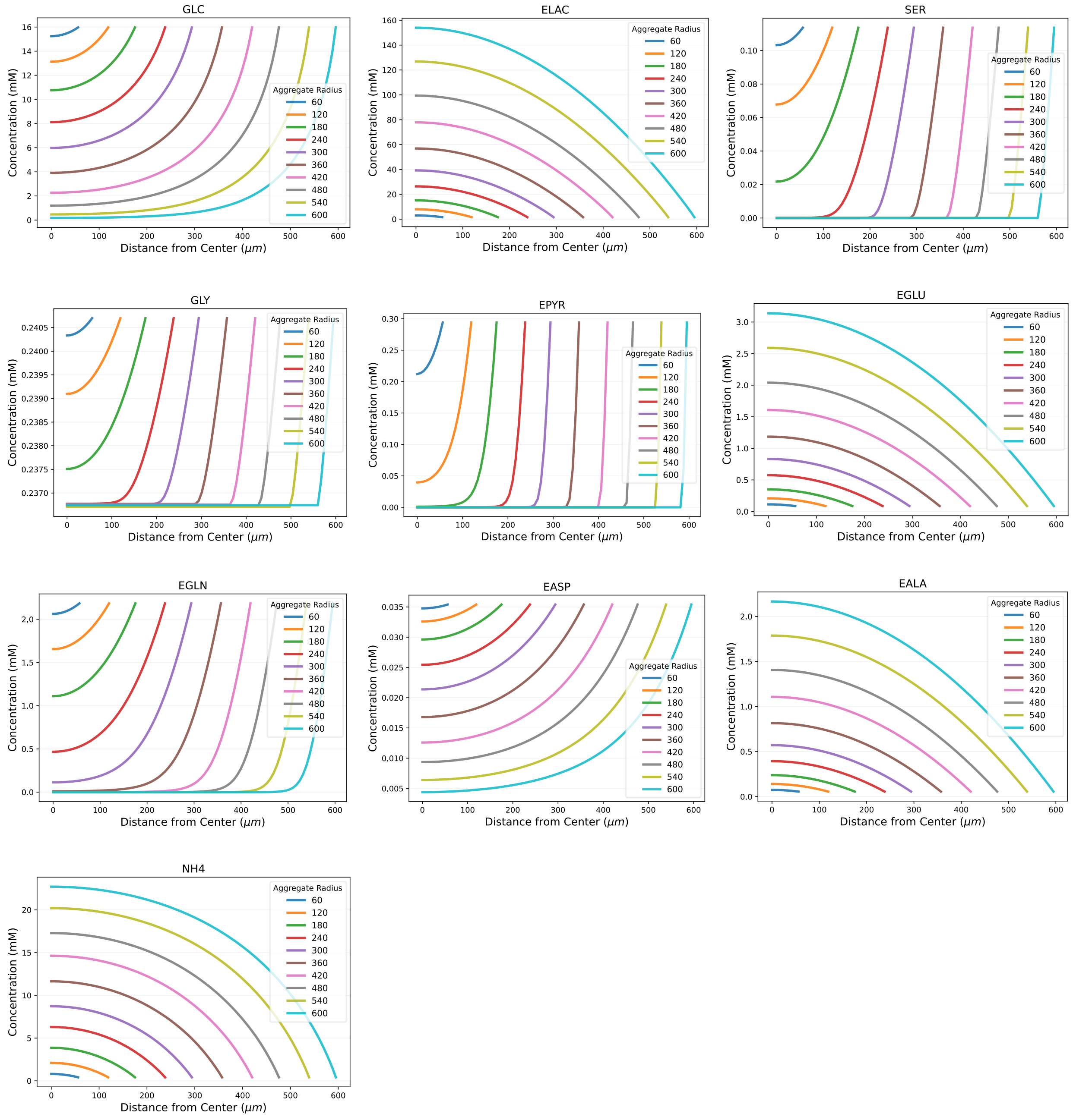

Supplementary Fig. 3. The plot shows the predicted biomass production of different aggregates (ranging from 30 μm to 300 μm in radius) for a duration of one hour based on the simulation results with 100 replicates. The recorded results correspond to 0, 6, 12, …, 72 culture hour respectively. The blue line represents the mean, and the red line represents the RSD of the biomass (i.e., $RSD_{biomass,\ell,t}$ with $\ell = 30,45,\ldots,300$).

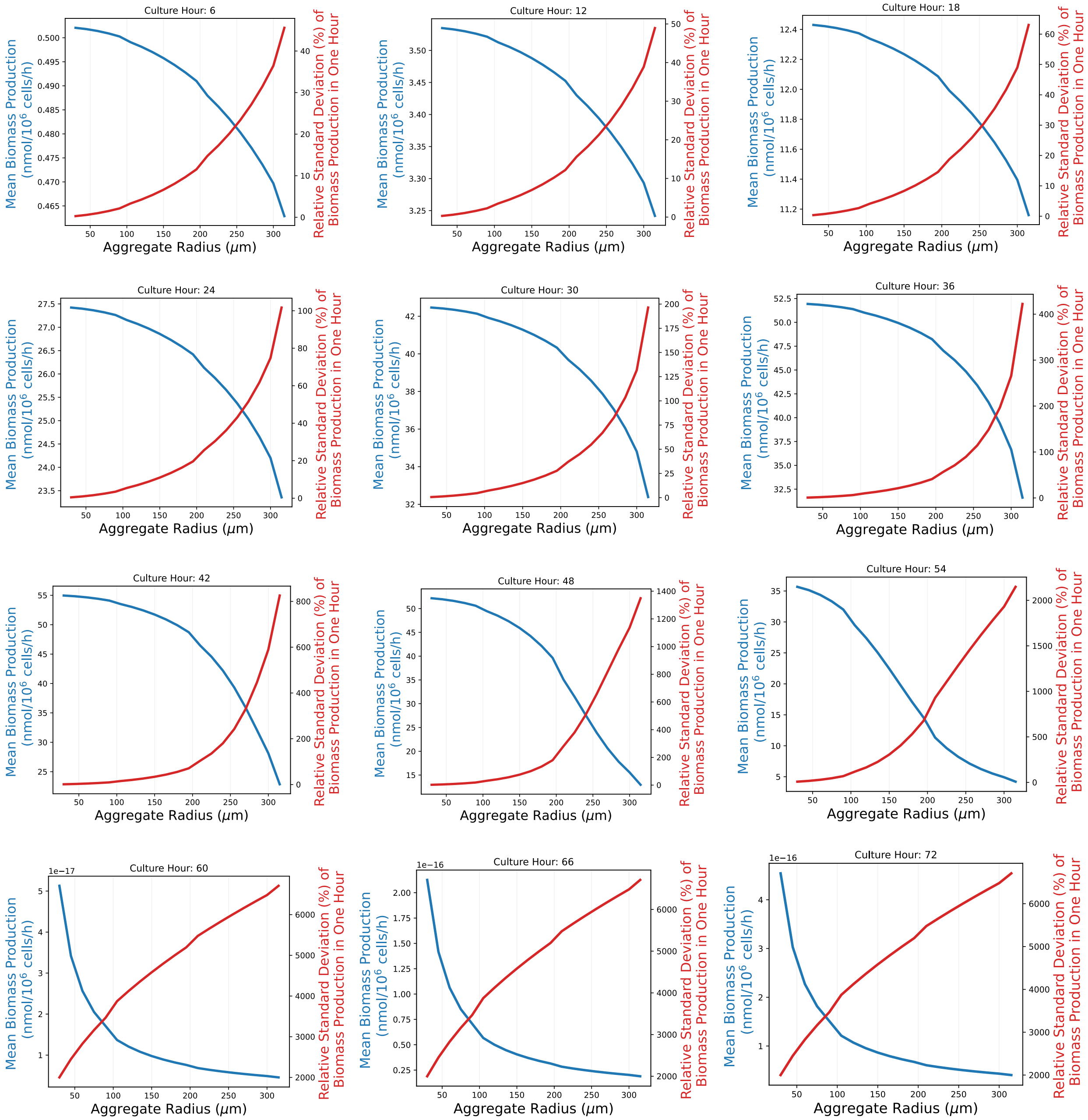

Supplementary Table 1 Reactions of the metabolic network

No.	**Glycolysis**
1	$$v(HK) = v_{max,HK} \cdot \frac{GLC}{k_{m,GLC} + GLC} \cdot \frac{K_{i,G6P}}{K_{i,G6P} + G6P} \cdot \frac{K_{i,LACtoHK}}{K_{i,LACtoHK} + LAC}$$
2	$$v(PGI) = v_{max,PGI} \cdot \frac{G6P}{K_{m,G6P} + G6P}$$
3	$$v(PFK/ALD) = v_{max,PFK/ALD} \cdot \frac{F6P}{K_{m,F6P} + F6P}$$
4	$$v(PGK) = v_{max,PGK} \cdot \frac{GAP}{K_{m,GAP} + GAP}$$
5	$$v(PK) = v_{max,PK} \cdot \frac{PEP}{K_{m,PEP} \cdot \left(1 + \frac{K_{a,F6P}}{F6P}\right) + PEP}$$
6f	$$v(LDHf) = v_{max,fLDH} \cdot \frac{PYR}{K_{m,PYR} + PYR}$$
6r	$$v(LDHr) = v_{max,rLDH} \cdot \frac{LAC}{K_{m,LAC} + LAC} \cdot \frac{K_{i,PYR}}{K_{i,PYR} + PYR}$$
7	$$v(PyrT) = v_{max,PyrT} \cdot \frac{EPYR}{K_{m,EPYR} + EPYR} \cdot \frac{K_{i,LACtoPYR}}{K_{i,LACtoPYR} + LAC}$$
8f	$$v(LacTf) = v_{max,fLacT} \cdot \frac{LAC}{K_{m,LAC} + LAC}$$
8r	$$v(LacTr) = v_{max,rLacT} \cdot \frac{ELAC}{K_{m,ELAC} + ELAC}$$
	**PPP**
9	$$v(OP) = v_{max,OP} \cdot \frac{G6P}{K_{m,G6P} + G6P}$$
10	$$v(NOP) = v_{max,NOP} \cdot \frac{Ru5P}{K_{m,Ru5P} + Ru5P}$$
	**TCA**

11	$v(PDH) = v_{max,PDH} \cdot \frac{PYR}{K_{m,PYR} + PYR} \cdot$
12	$v(CS) = v_{max,CS} \cdot \frac{AcCoA}{K_{m,AcCoA} + AcCoA} \cdot \frac{OAA}{K_{m,OAA} + OAA}$
13f	$v(CITS/ISODf) = v_{max,fCITS/ISOD} \cdot \frac{CIT}{K_{m,CIT} + CIT}$
13r	$v(CITS/ISODr) = v_{max,rCITS/ISOD} \cdot \frac{AKG}{K_{m,AKG} + AKG}$
14	$v(AKGDH) = v_{max,AKGDH} \cdot \frac{AKG}{K_{m,AKG} + AKG}$
15	$v(SDH) = v_{max,SDH} \cdot \frac{SUC}{K_{m,SUC} + SUC}$
16f	$v(FUMf) = v_{max,fFUM} \cdot \frac{FUM}{K_{m,FUM} + FUM}$
16r	$v(FUMr) = v_{max,rFUM} \cdot \frac{MAL}{K_{m,MAL} + MAL}$
17f	$v(MDHf) = v_{max,fMDH} \cdot \frac{MAL}{K_{m,MAL} + MAL}$
17r	$v(MDHr) = v_{max,rMDH} \cdot \frac{OAA}{K_{m,OAA} + OAA}$
	**Anaplerosis and Amino Acid**
18	$v(ME) = v_{max,ME} \cdot \frac{MAL}{K_{m,MAL} + MAL}$
19	$v(PC) = v_{max,PC} \cdot \frac{PYR}{K_{m,PYR} + PYR}$
20f	$v(GLNSf) = v_{max,fGLNS} \cdot \frac{GLN}{K_{m,GLN} + GLN} \cdot \frac{K_{i,LACtoGLNS}}{K_{i,LACtoGLNS} + LAC}$
20r	$v(GLNSr) = v_{max,rGLNS} \cdot \frac{GLU}{K_{m,GLU} + GLU} \cdot \frac{NH_4}{K_{m,NH_4} + NH_4}$

21f	$$v(GLDHf) = v_{max,fGLDH} \cdot \frac{GLU}{K_{m,GLU} + GLU}$$
21r	$$v(GLDHr) = v_{max,rGLDH} \cdot \frac{AKG}{K_{m,AKG} + AKG} \cdot \frac{NH_4}{K_{m,NH_4} + NH_4}$$
22f	$$v(AlaTAf) = v_{max,fAlaTA} \cdot \frac{GLU}{K_{m,GLU} + GLU} \cdot \frac{PYR}{K_{m,PYR} + PYR}$$
22r	$$v(AlaTAr) = v_{max,rAlaTA} \cdot \frac{ALA}{K_{m,Ala} + Ala} \cdot \frac{AKG}{K_{m,AKG} + AKG} \cdot \left(1 + \frac{K_{a,GLN}}{GLN}\right)$$
23	$$v(AlaT) = v_{max,AlaT} \cdot \frac{ALA}{K_{m,ALA} + ALA}$$
24	$$v(GluT) = v_{max,GluT} \cdot \frac{GLU}{K_{m,GLU} + GLU}$$
25	$$v(GlnT) = v_{max,GlnT} \cdot \frac{EGLN}{K_{m,EGLN} + EGLN} \cdot \frac{K_{i,GLN}}{K_{i,GLN} + GLN}$$
26	$$v(SAL) = v_{max,SAL} \cdot \frac{SER}{K_{m,Ser} + SER}$$
27f	$$v(ASTAf) = v_{max,fASTA} \cdot \frac{ASP}{K_{m,ASP} + ASP} \cdot \frac{AKG}{K_{m,AKG} + AKG}$$
27r	$$v(AspT) = v_{max,AspT} \cdot \frac{EASP}{K_{m,EASP} + EASP}$$
28	$$v(AspT) = v_{max,AspT} \cdot \frac{EASP}{K_{m,EASP}}$$
29	$$v(ACL) = v_{max,ACL} \cdot \frac{CIT}{K_{m,CIT} + CIT}$$
	**Biomass**
30	$$v(Biomass) = v_{max,Biomass} \cdot \frac{GLN}{K_{m,GLN} + GLN} \cdot \frac{GLC}{K_{m,GLC} + GLC} \cdot \frac{GLU}{K_{m,GLU} + GLU} \cdot \frac{ALA}{K_{m,ALA} + ALA} \cdot \frac{ASP}{K_{m,ASP} + ASP} \cdot \frac{SER}{K_{m,SER} + SER} \cdot \frac{GLY}{K_{m,GLY} + GLY}$$

Supplementary Table 2 Description of Metabolites

Component	Description	Component	Description
**ACCoA**	Acetyl-CoezymeA	ALA	Alanine
**AKG**	α-Ketoglutarate	ASP	Aspartate
**CIT**	Citrate	LAC	Lactate
**CO2**	Intracellular Carbonoxygen	GLN	Glutamine
**F6P**	Fructose 6-Phosphate	EGLY	Extracellular Glycine
**G6P**	Glucose 6-Phosphate	SER	Extracellular Serine
**GAP**	Glyceraldehyde 3-Phosphate	GLC	Extracellular Glucose
**GLU**	Glutamate	EGLN	Extracellular Glutamine
**GLY**	Glycine	EGLU	Extracellular Glutamate
**MAL**	Malate	EPYR	Extracellular Pyruvate
**OAA**	Oxaloacetate	EASP	Extracellular Aspartate
**PEP**	Phosphoenolpyruvate	EALA	Extracellular Alanine
**FUM**	Fumarate	ELAC	Extracellular Lactate
**Ru5P**	Ribulose 5-Phosphate	NH4	Extracellular Ammonia
**SUC**	Succinate	LIPID	Lipid
**PYR**	Pyruvate	Bio	Cell Density

Supplementary Table 3 Description of Enzymes

**Abbreviation**	**Description**	**EC-No.**
**HK**	Hexokinase	2.7.1.1
**PGI**	Phosphoglucose Isomerase	5.3.1.9
**PFK/ALD**	Phosphofructokinase/Aldolase	2.7.1.11/4.1.2.13
**PGK**	Phosphoglycerate Kinase	2.7.2.3
**PK**	Pyruvate Kinase	2.7.1.40
**OP**	Oxidative Phase of PPP	
**NOP**	Non-oxidative Phase of PPP	
**PyrT**	Membrane Transport of Pyruvate	
**SAL**	Membrane Transport of Serine	
**LDH**	Lactate Dehydrogenase	1.1.1.27
**AlaTA**	Alanine Transaminase	2.6.1.2
**PC**	Pyruvate Carboxylase	6.4.1.1
**PDH**	Pyruvate Dehydrogenase	1.2.4.1
**CS**	Citrate (Si)-Synthase	2.3.3.1
**CITS/ISOD**	Aconitase/Isocitrate Dehydrogenase	4.2.1.3/1.1.1.41
**GLDH**	Glutamate Dehydrogenase	1.4.1.2
**GluT**	Membrane Transport of Glutamate	
**GLNS**	Glutamine Synthetase	6.3.1.2
**AKGDH**	α-ketoglutarate Dehydrogenase	1.2.1.105
**SDH**	Succinate Dehydrogenase	1.3.5.1
**MDH**	Malate Dehydrigenase	1.1.1.37

**ME**	Malic Enzyme	1.1.1.40
**ASTA**	Aspartate Aminotransferase	2.6.1.1
**ACL**	ATP citrate synthase	2.3.3.8
**FUM**	Fumarase	4.2.1.2

Supplementary Table 4. Diffusion Coefficients for Extracellular Metabolites (10^{-9} m^2/s)

Metabolite	**Diffusion Coefficients** (D_i^a)	Metabolite	**Diffusion Coefficients** (D_i^a)
Pyruvate	1.12[49]	Glucose	0.6[51]
Alanine	0.91[50]	Glutamine	0.76[52]
Aspartate	0.741[53]	Glutamate	0.708[54]
Glycine	1.04[50]	Lactate	1.033[49]
Serine	0.891[50]	Ammonia	1.86[55]

Supplementary Table 5. Comparison of reaction flux rates between outer and inner cells in aggregates with a radius of 60, 120,240 and 360 μm at 24 hours.

Aggregate Radius	60 μm		120 μm		240 μm		360 μm	
**Reaction**	Outer Cells	Inner Cells	Outer Cells	Inner Cells	Outer Cells	Inner Cells	Outer Cells	Inner Cells
**HK**	1460.62	1425.16	1460.62	1349.82	1460.62	1134.31	1460.85	927.78
**PGI**	1202.59	1193.26	1202.59	1171.14	1202.59	1085.17	1202.50	882.00
**PEKALD**	1177.50	1169.23	1177.50	1149.71	1177.50	1074.26	1177.37	864.47
**PGK**	2282.12	2267.92	2282.12	2234.48	2282.12	2105.11	2281.76	1679.23
**PK**	2175.71	2164.09	2175.71	2136.83	2175.71	2031.51	2175.26	1607.29
**LDHf**	2077.26	2065.22	2077.26	2035.65	2077.26	1920.72	2076.80	1536.53
**LDHr**	80.42	80.82	80.42	81.06	80.42	79.50	80.41	69.00
**PyrT**	92.29	85.60	92.29	69.14	92.29	15.40	92.29	0.27
**LacTf**	2353.55	2364.75	2353.55	2370.20	2353.55	2319.61	2353.15	2002.84
**LacTr**	478.28	498.90	478.28	527.27	478.28	563.52	478.28	578.95
**OP**	10.15	10.07	10.15	9.88	10.15	9.16	10.15	7.44
**NOP**	3.27	3.25	3.27	3.19	3.27	2.97	3.27	2.26
**PDH**	139.79	138.98	139.79	136.99	139.79	129.26	139.76	103.40
**CS**	127.10	126.53	127.10	125.10	127.10	119.24	127.06	101.82
**CITSISODf**	36.02	35.86	36.02	35.45	36.02	33.81	36.01	29.33
**CITSISODr**	5.56	5.49	5.56	5.39	5.56	5.07	5.55	4.65
**AKGDH**	126.00	124.56	126.00	122.28	126.00	115.01	125.96	105.37
**SDH**	136.59	136.03	136.59	135.14	136.59	132.13	136.62	148.53
**FUMf**	159.99	159.88	159.99	159.69	159.99	159.04	160.01	165.23
**FUMr**	6.80	6.79	6.80	6.77	6.80	6.67	6.80	6.54
**MLDf**	242.60	242.22	242.60	241.35	242.60	237.95	242.59	233.33
**MLDr**	81.01	80.98	81.01	80.96	81.01	80.96	81.01	79.53
**ME**	84.95	84.82	84.95	84.51	84.95	83.32	84.95	81.70
**PC**	39.00	38.77	39.00	38.22	39.00	36.06	38.99	28.85
**GLNSf**	254.08	254.34	254.08	252.51	254.08	238.57	254.06	202.43
**GLNSr**	60.54	63.67	60.54	67.07	60.54	67.77	60.52	52.58
**GLDHf**	85.23	83.11	85.23	80.39	85.23	74.42	85.20	55.94
**GLDHr**	3.03	3.23	3.03	3.46	3.03	3.55	3.03	3.36

**AlaTAf**	171.37	166.14	171.37	158.40	171.37	138.36	171.26	83.19
**AlaTAr**	139.01	133.77	139.01	126.57	139.01	109.85	138.91	65.69
**AlaT**	24.61	24.13	24.61	23.42	24.61	21.30	24.59	12.24
**GluT**	55.04	53.67	55.04	51.92	55.04	48.06	55.02	36.12
**GlnT**	199.84	196.51	199.84	190.90	199.84	175.92	199.85	159.14
**SAL**	11.28	11.23	11.28	11.09	11.28	10.28	11.28	6.08
**ASTAf**	0.01	0.01	0.01	0.01	0.01	0.01	0.01	0.01
**ASTAr**	5.80	6.10	5.80	6.43	5.80	6.49	5.80	4.95
**ASPT**	1.09	1.09	1.09	1.07	1.09	1.00	1.09	0.90
**ACL**	94.37	93.94	94.37	92.89	94.37	88.59	94.34	76.85
**Biomass**	30.88	33.90	30.88	35.21	30.88	29.00	30.83	3.32
**Sum Flux**	15715.36	15634.04	15715.36	15411.55	15715.36	14492.93	15713.21	12009.12
**Difference of Sum Fluxes**	81.32		303.81		1222.43		3704.09	

Supplementary Table 6 Comparison of reaction flux rates between outer and inner cells in aggregates with a radius of 60, 120,240 and 360 μm at 48 hours.

Aggregate Radius	60 μm		120 μm		240 μm		360 μm	
**Reaction**	Outer Cells	Inner Cells	Outer Cells	Inner Cells	Outer Cells	Inner Cells	Outer Cells	Inner Cells
**HK**	958.80	950.99	958.80	930.32	958.80	834.98	958.82	691.52
**PGI**	1152.67	1134.02	1152.67	1088.34	1152.67	913.23	1152.65	713.28
**PEKALD**	1176.10	1158.30	1176.10	1114.16	1176.10	937.13	1176.08	725.62
**PGK**	2382.12	2348.27	2382.12	2263.44	2382.12	1908.75	2382.07	1466.69
**PK**	2407.84	2376.52	2407.84	2297.16	2407.84	1947.74	2407.78	1483.59
**LDHf**	2413.83	2384.48	2413.83	2311.43	2413.83	1983.97	2419.50	1542.15
**LDHr**	94.82	94.63	94.82	93.96	94.82	87.31	94.80	69.51
**PyrT**	19.71	16.06	19.71	9.52	19.71	1.05	19.71	0.03
**LacTf**	2803.66	2794.63	2803.66	2767.51	2803.66	2550.49	2803.53	2017.84
**LacTr**	568.73	570.49	568.73	573.86	568.73	581.28	568.73	586.22
**OP**	9.73	9.57	9.73	9.19	9.73	7.71	9.73	6.02
**NOP**	3.29	3.24	3.29	3.12	3.29	2.64	3.29	2.05
**PDH**	162.44	160.47	162.44	155.55	162.44	133.51	162.82	103.78
**CS**	163.88	162.14	163.88	157.84	163.88	138.47	163.84	104.27
**CITSISODf**	47.20	46.70	47.20	45.49	47.20	40.09	47.32	31.19
**CITSISODr**	6.92	6.83	6.92	6.64	6.91	5.97	6.71	4.35
**AKGDH**	156.82	154.81	156.82	150.65	156.82	135.43	152.07	98.69
**SDH**	153.60	151.83	153.60	148.47	153.60	136.73	153.74	115.77
**FUMf**	155.06	153.66	155.06	151.14	155.06	142.42	155.04	127.58
**FUMr**	7.40	7.35	7.40	7.23	7.40	6.77	7.38	11.96
**MLDf**	263.98	262.03	263.98	257.92	263.98	241.53	263.26	426.62
**MLDr**	85.40	85.36	85.40	85.32	85.40	85.23	85.40	84.55
**ME**	92.44	91.75	92.44	90.32	92.44	84.58	92.19	149.39
**PC**	45.31	44.76	45.31	43.39	45.31	37.25	45.42	28.95
**GLNSf**	231.57	229.56	231.57	224.98	231.57	209.34	230.77	174.56
**GLNSr**	68.44	68.21	68.44	67.76	68.44	67.33	70.36	67.17
**GLDHf**	77.75	76.84	77.75	75.20	77.75	72.62	79.94	71.18
**GLDHr**	4.68	4.66	4.68	4.60	4.68	4.26	4.54	3.16

**AlaTAf**	181.65	177.35	181.65	168.24	181.66	139.44	187.20	106.24
**AlaTAr**	148.56	144.91	148.56	137.60	148.53	117.27	65.23	0.00
**AlaT**	23.45	23.04	23.45	22.20	23.45	20.00	10.44	0.00
**GluT**	50.21	49.62	50.21	48.56	50.21	46.89	51.62	45.96
**GlnT**	167.66	165.68	167.66	160.91	167.66	141.28	168.29	110.69
**SAL**	9.23	8.89	9.23	7.82	9.23	0.84	9.23	0.00
**ASTAf**	0.03	0.03	0.03	0.03	0.03	0.05	0.03	0.04
**ASTAr**	6.92	6.89	6.92	6.84	6.92	6.79	7.11	6.72
**ASPT**	0.96	0.95	0.96	0.94	0.96	0.88	0.96	0.79
**ACL**	123.65	122.35	123.65	119.17	123.65	105.03	123.96	81.72
**Biomass**	53.47	52.07	53.47	46.93	53.46	6.41	24.06	0.00
**Sum Flux**	16479.95	16299.95	16479.95	15853.77	16479.91	13882.69	16365.61	11259.87
**Difference of Sum Fluxes**	180.00		626.18		2597.22		5105.75	
