# Supplementary material for: Stochastic biological system-of-systems modelling for iPSC culture
Source: Commun Biol. 2024 Jan 8;7:39. doi: 10.1038/s42003-023-05653-w (PMC10774284; doi:10.1038/s42003-023-05653-w)
Supplement: Supplementary file 3 — Supplementary Software [file 42003_2023_5653_MOESM3_ESM.zip › MultiScaleModel-master/multi_scale_model/result/flux_rate/flux_comparison.pdf]

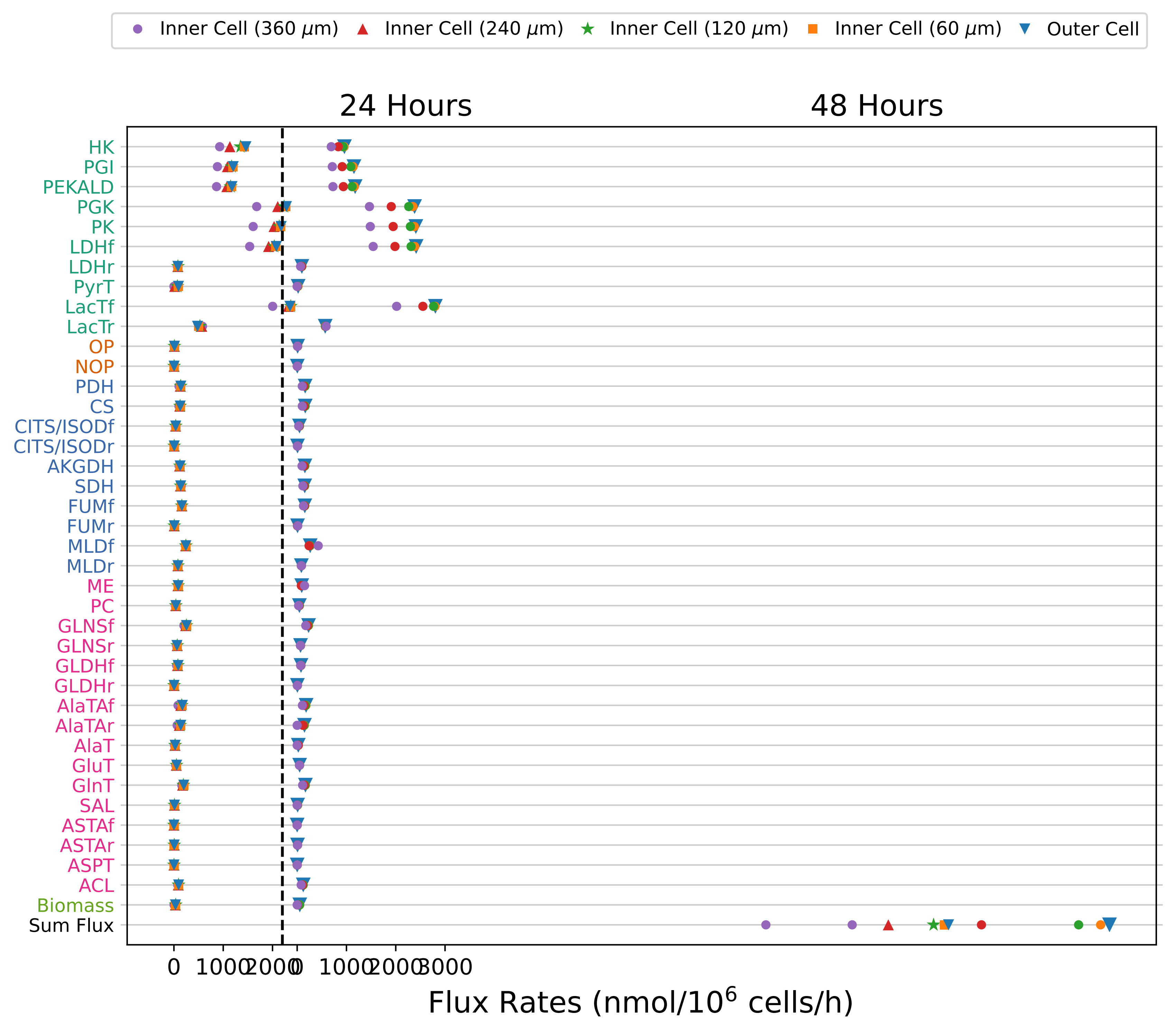

Inner Cell (360 μm)
Inner Cell (240 μm)
Inner Cell (120 μm)
Inner Cell (60 μm)
Outer Cell
24 Hours
48 Hours
HK
PGI
PEKALD
PGK
PK
LDHf
LDHr
PyrT
LacTf
LacTr
OP
NOP
PDH
CS
CITS/ISODf
CITS/ISODr
AKGDH
SDH
FUMf
FUMr
MLDf
MLDr
ME
PC
GLNSf
GLNSr
GLDHf
GLDHr
AlaTAf
AlaTAr
AlaT
GluT
GlnT
SAL
ASTAf
ASTAr
ASPT
ACL
Biomass
Sum Flux
0
1000
2000
0
1000
2000
3000
Flux Rates (nmol/10^6 cells/h)
