# Supplementary material for: Stochastic biological system-of-systems modelling for iPSC culture
Source: Commun Biol. 2024 Jan 8;7:39. doi: 10.1038/s42003-023-05653-w (PMC10774284; doi:10.1038/s42003-023-05653-w)
Supplement: Supplementary file 3 — Supplementary Software [file 42003_2023_5653_MOESM3_ESM.zip › MultiScaleModel-master/multi_scale_model/result/optimal_size/Biomass-reaction-ala-0.15.pdf]

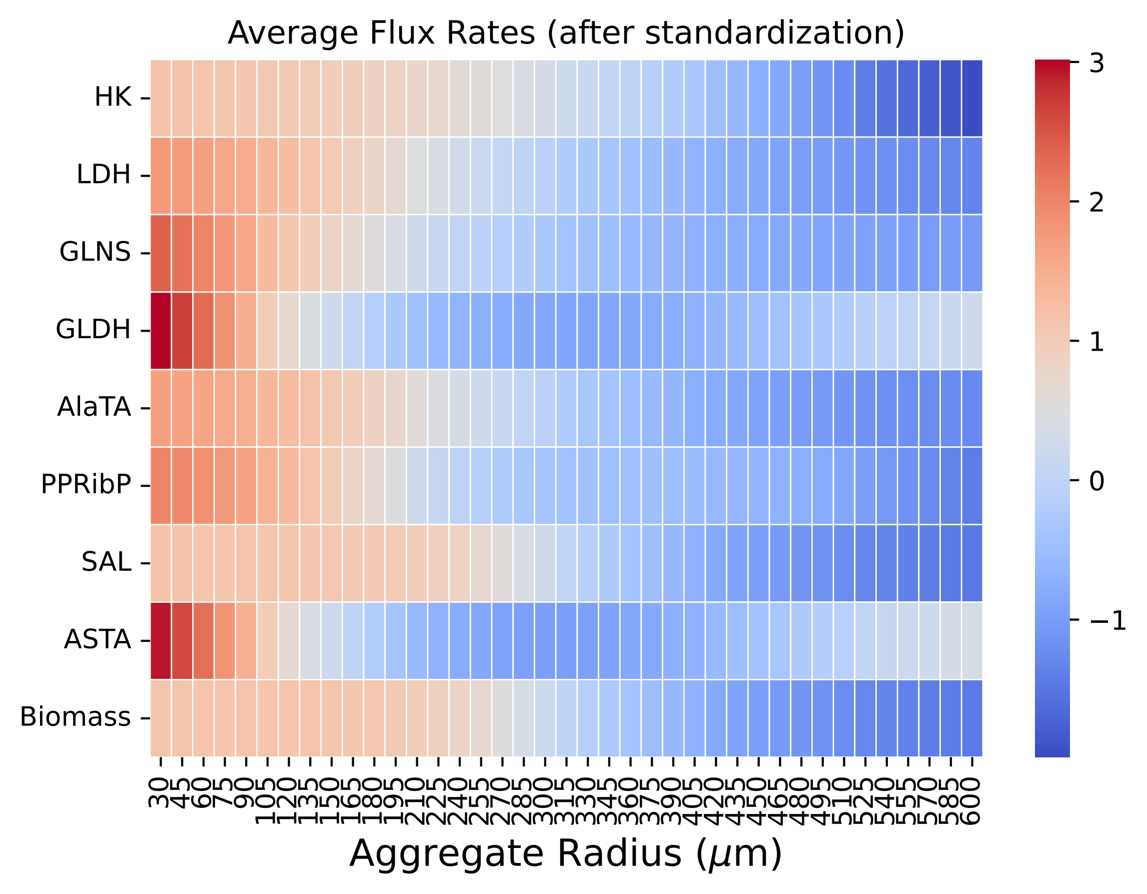

Average Flux Rates (after standardization)
HK
LDH
GLNS
GLDH
AlaTA
PPRibP
SAL
ASTA
Biomass
30 45 60 75 90 105 120 135 150 165 180 195 210 225 240 255 270 285 300 315 330 345 360 375 390 405 420 435 450 465 480 495 510 525 540 555 570 585 600
Aggregate Radius (μm)
3
2
1
0
−1
