# Supplementary material for: Stochastic biological system-of-systems modelling for iPSC culture
Source: Commun Biol. 2024 Jan 8;7:39. doi: 10.1038/s42003-023-05653-w (PMC10774284; doi:10.1038/s42003-023-05653-w)
Supplement: Supplementary file 3 — Supplementary Software [file 42003_2023_5653_MOESM3_ESM.zip › MultiScaleModel-master/multi_scale_model/result/optimal_size/Biomass-reaction-ala-0.5.pdf]

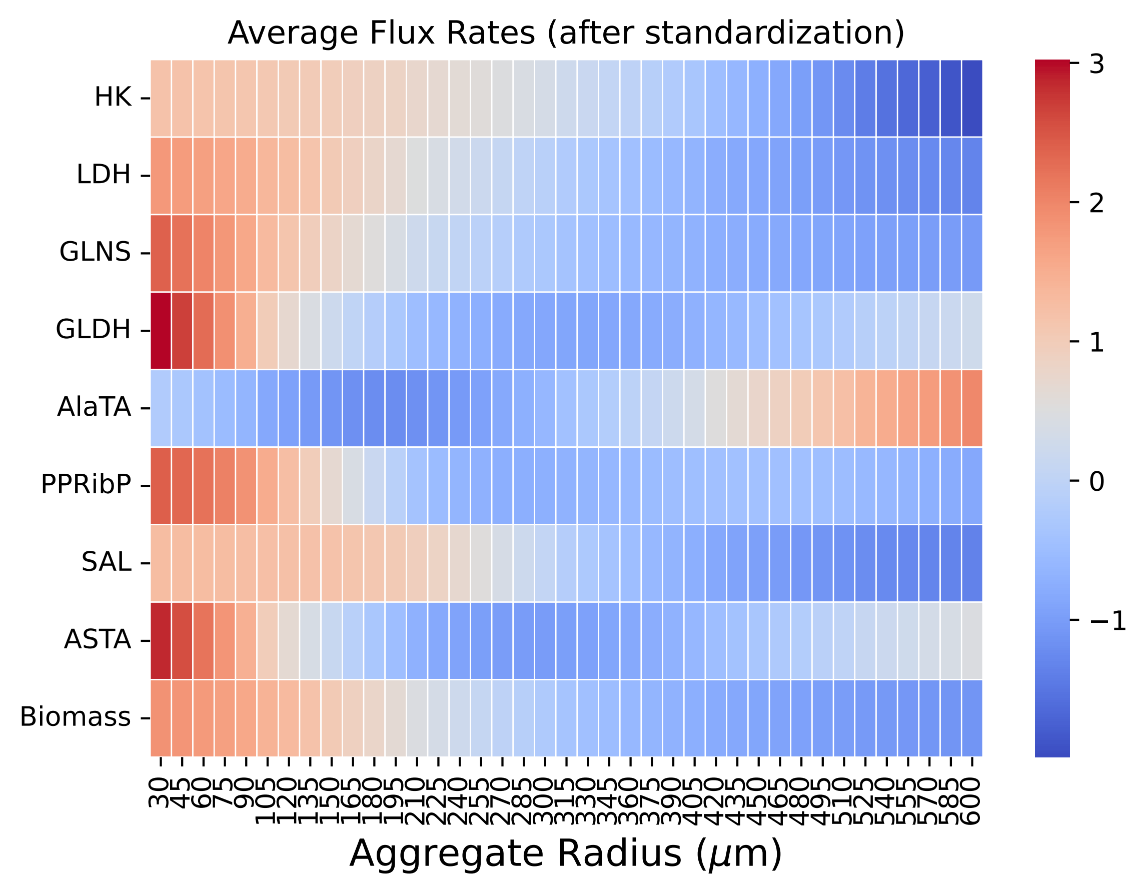
Average Flux Rates (after standardization)
HK
LDH
GLNS
GLDH
AlaTA
PPRibP
SAL
ASTA
Biomass
Aggregate Radius (μm)
3
2
1
0
−1
