# Supplementary figures and images for: Stochastic biological system-of-systems modelling for iPSC culture (part 2 of 2)
Source: Commun Biol. 2024 Jan 8;7:39. doi: 10.1038/s42003-023-05653-w (PMC10774284; doi:10.1038/s42003-023-05653-w)

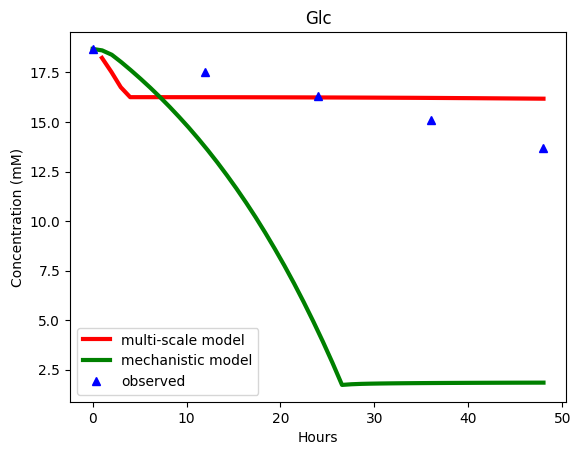

Supplement: Supplementary file 3 — Supplementary Software [file 42003_2023_5653_MOESM3_ESM.zip › MultiScaleModel-master/multi_scale_model/result/simulation/HGHL-Glc.png]

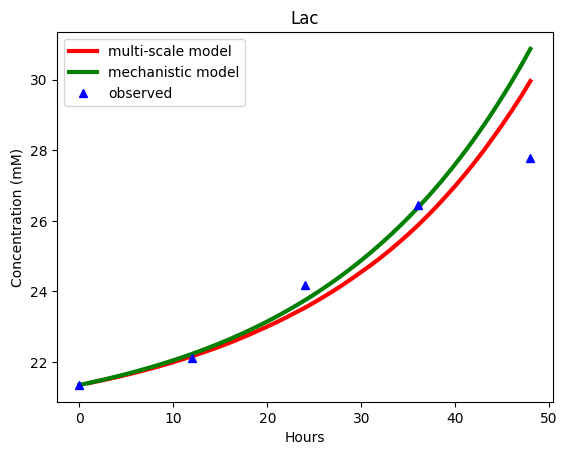

Supplement: Supplementary file 3 — Supplementary Software [file 42003_2023_5653_MOESM3_ESM.zip › MultiScaleModel-master/multi_scale_model/result/simulation/HGHL-Lac.png]

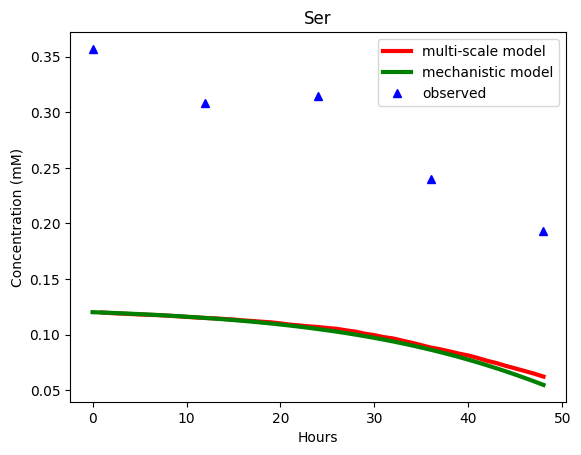

Supplement: Supplementary file 3 — Supplementary Software [file 42003_2023_5653_MOESM3_ESM.zip › MultiScaleModel-master/multi_scale_model/result/simulation/HGHL-Ser.png]

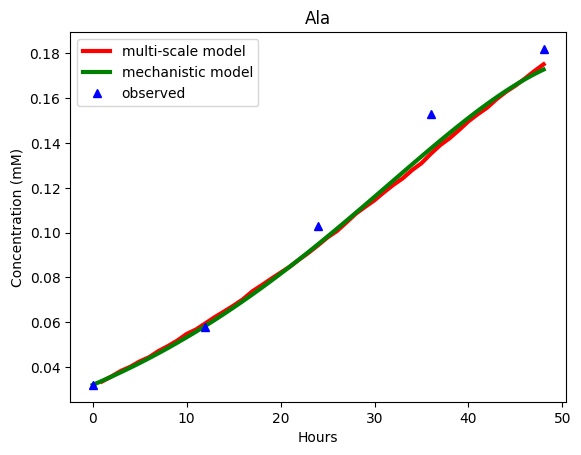

Supplement: Supplementary file 3 — Supplementary Software [file 42003_2023_5653_MOESM3_ESM.zip › MultiScaleModel-master/multi_scale_model/result/simulation/HGLL-Ala.png]

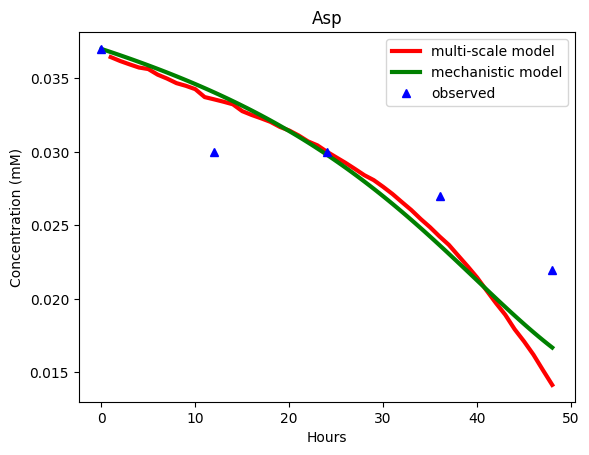

Supplement: Supplementary file 3 — Supplementary Software [file 42003_2023_5653_MOESM3_ESM.zip › MultiScaleModel-master/multi_scale_model/result/simulation/HGLL-Asp.png]

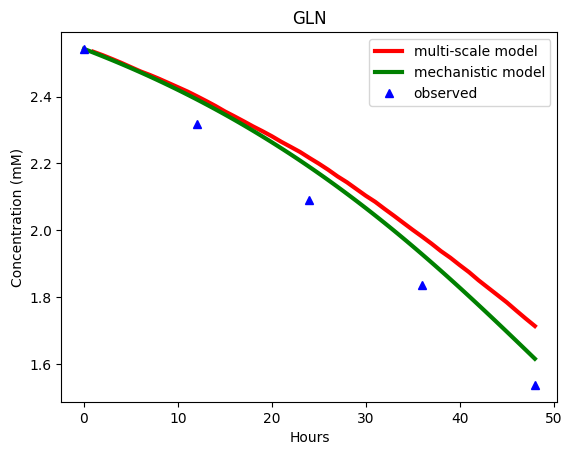

Supplement: Supplementary file 3 — Supplementary Software [file 42003_2023_5653_MOESM3_ESM.zip › MultiScaleModel-master/multi_scale_model/result/simulation/HGLL-GLN.png]

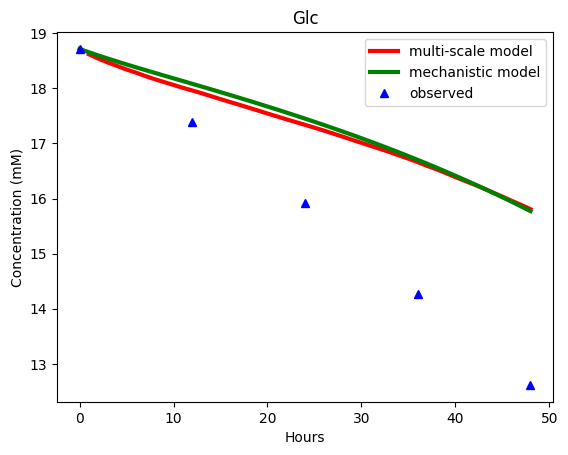

Supplement: Supplementary file 3 — Supplementary Software [file 42003_2023_5653_MOESM3_ESM.zip › MultiScaleModel-master/multi_scale_model/result/simulation/HGLL-Glc.png]

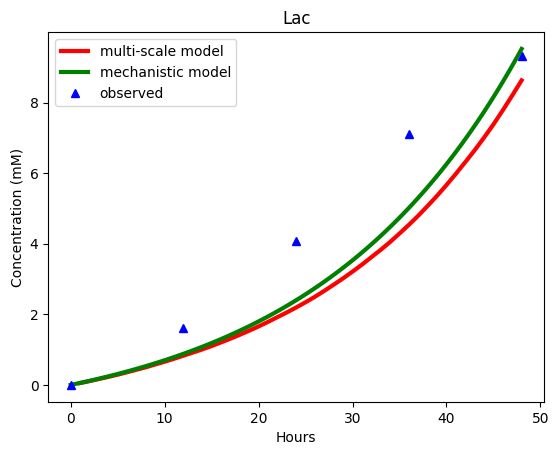

Supplement: Supplementary file 3 — Supplementary Software [file 42003_2023_5653_MOESM3_ESM.zip › MultiScaleModel-master/multi_scale_model/result/simulation/HGLL-Lac.png]

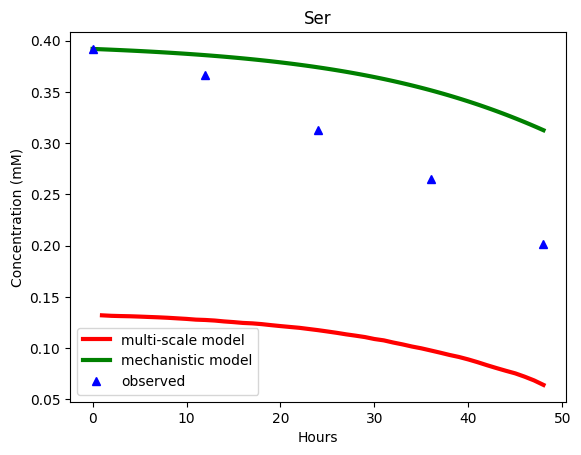

Supplement: Supplementary file 3 — Supplementary Software [file 42003_2023_5653_MOESM3_ESM.zip › MultiScaleModel-master/multi_scale_model/result/simulation/HGLL-Ser.png]

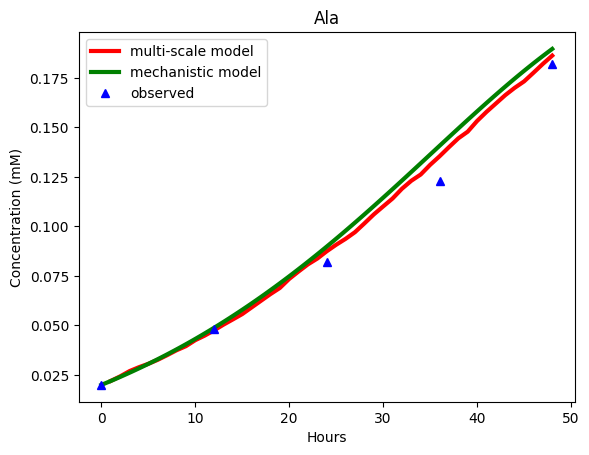

Supplement: Supplementary file 3 — Supplementary Software [file 42003_2023_5653_MOESM3_ESM.zip › MultiScaleModel-master/multi_scale_model/result/simulation/LGHL-Ala.png]

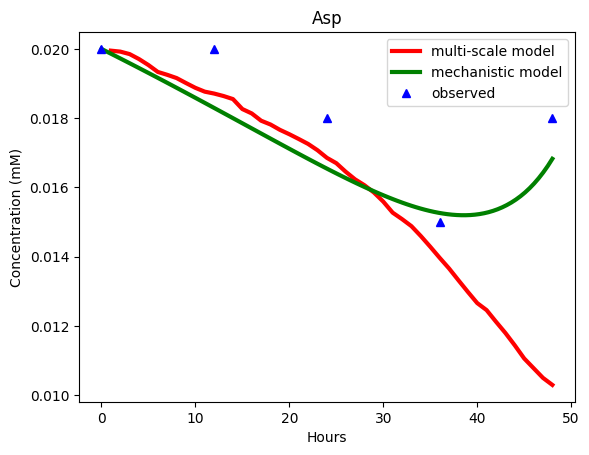

Supplement: Supplementary file 3 — Supplementary Software [file 42003_2023_5653_MOESM3_ESM.zip › MultiScaleModel-master/multi_scale_model/result/simulation/LGHL-Asp.png]

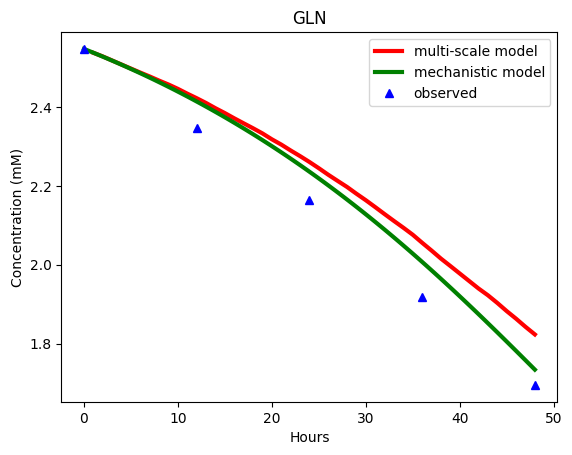

Supplement: Supplementary file 3 — Supplementary Software [file 42003_2023_5653_MOESM3_ESM.zip › MultiScaleModel-master/multi_scale_model/result/simulation/LGHL-GLN.png]

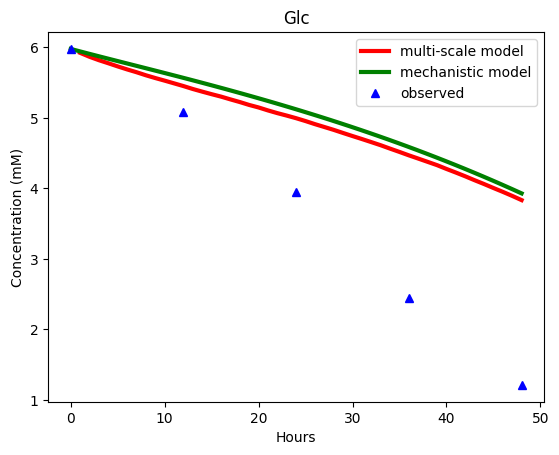

Supplement: Supplementary file 3 — Supplementary Software [file 42003_2023_5653_MOESM3_ESM.zip › MultiScaleModel-master/multi_scale_model/result/simulation/LGHL-Glc.png]

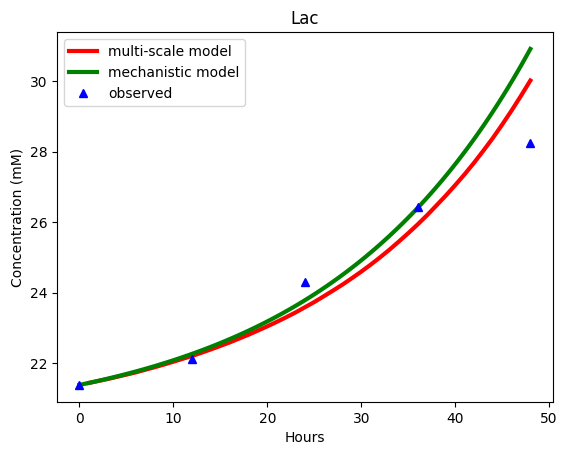

Supplement: Supplementary file 3 — Supplementary Software [file 42003_2023_5653_MOESM3_ESM.zip › MultiScaleModel-master/multi_scale_model/result/simulation/LGHL-Lac.png]

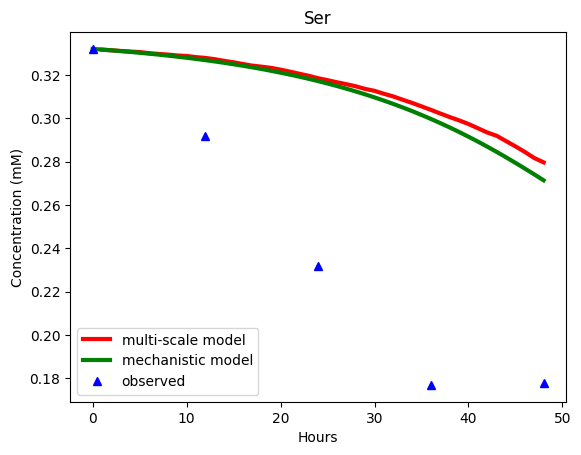

Supplement: Supplementary file 3 — Supplementary Software [file 42003_2023_5653_MOESM3_ESM.zip › MultiScaleModel-master/multi_scale_model/result/simulation/LGHL-Ser.png]

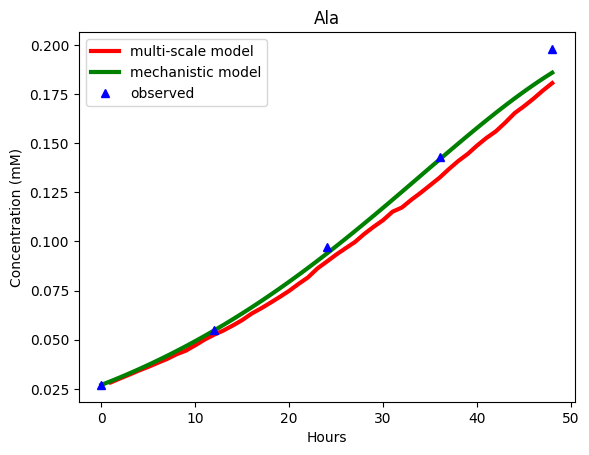

Supplement: Supplementary file 3 — Supplementary Software [file 42003_2023_5653_MOESM3_ESM.zip › MultiScaleModel-master/multi_scale_model/result/simulation/LGLL-Ala.png]

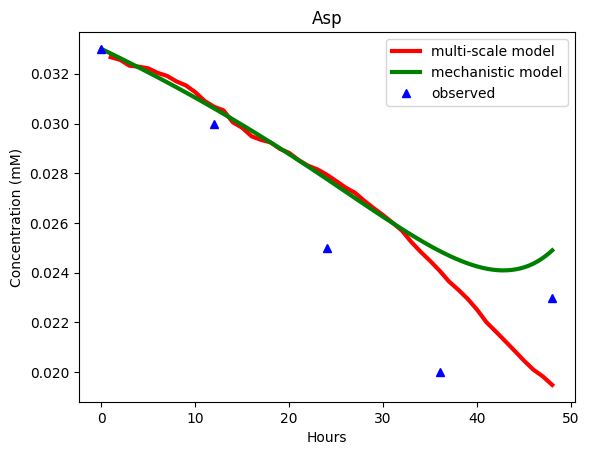

Supplement: Supplementary file 3 — Supplementary Software [file 42003_2023_5653_MOESM3_ESM.zip › MultiScaleModel-master/multi_scale_model/result/simulation/LGLL-Asp.png]

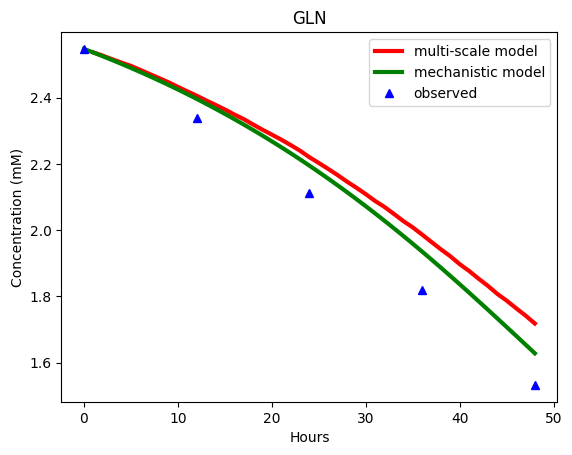

Supplement: Supplementary file 3 — Supplementary Software [file 42003_2023_5653_MOESM3_ESM.zip › MultiScaleModel-master/multi_scale_model/result/simulation/LGLL-GLN.png]

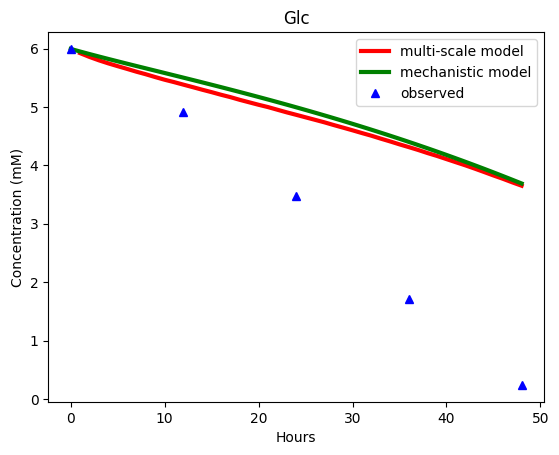

Supplement: Supplementary file 3 — Supplementary Software [file 42003_2023_5653_MOESM3_ESM.zip › MultiScaleModel-master/multi_scale_model/result/simulation/LGLL-Glc.png]

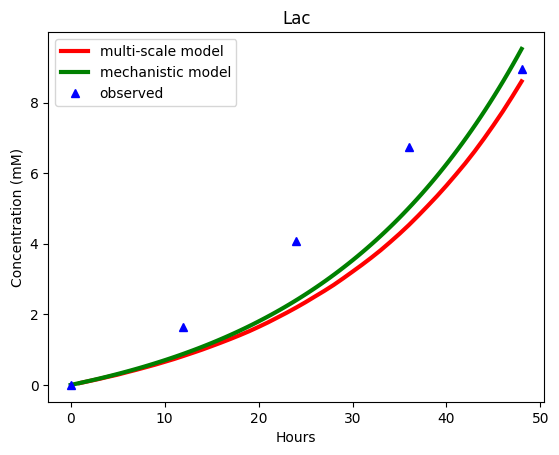

Supplement: Supplementary file 3 — Supplementary Software [file 42003_2023_5653_MOESM3_ESM.zip › MultiScaleModel-master/multi_scale_model/result/simulation/LGLL-Lac.png]

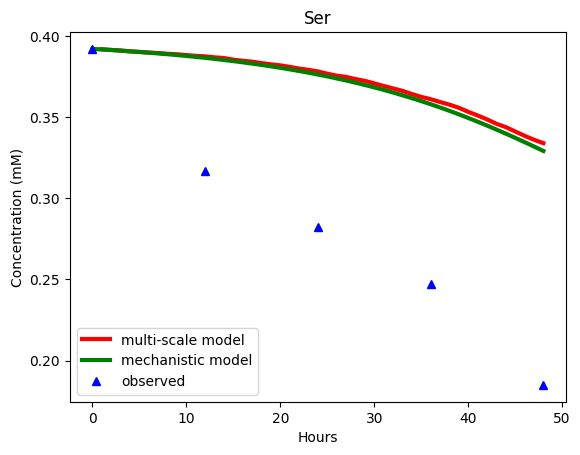

Supplement: Supplementary file 3 — Supplementary Software [file 42003_2023_5653_MOESM3_ESM.zip › MultiScaleModel-master/multi_scale_model/result/simulation/LGLL-Ser.png]

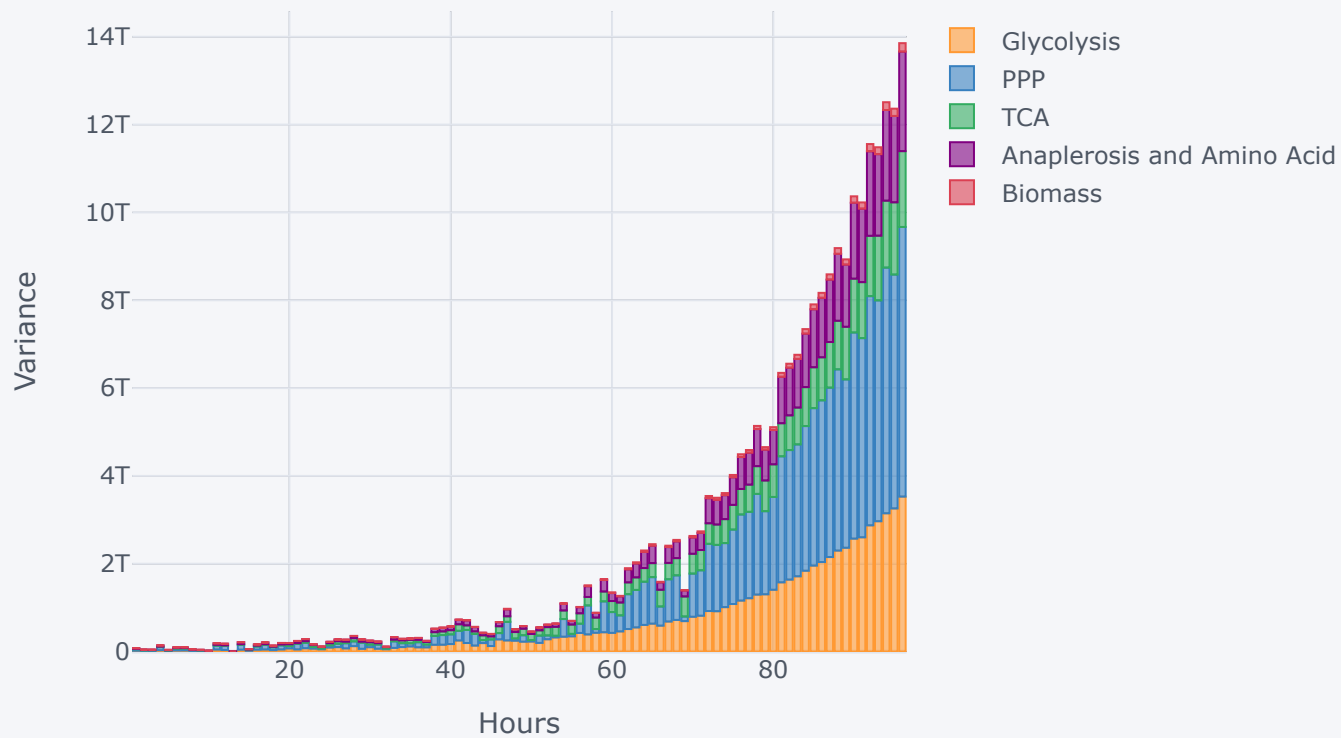

Supplement: Supplementary file 3 — Supplementary Software [file 42003_2023_5653_MOESM3_ESM.zip › MultiScaleModel-master/multi_scale_model/result/stacked_bar_plot.pdf]

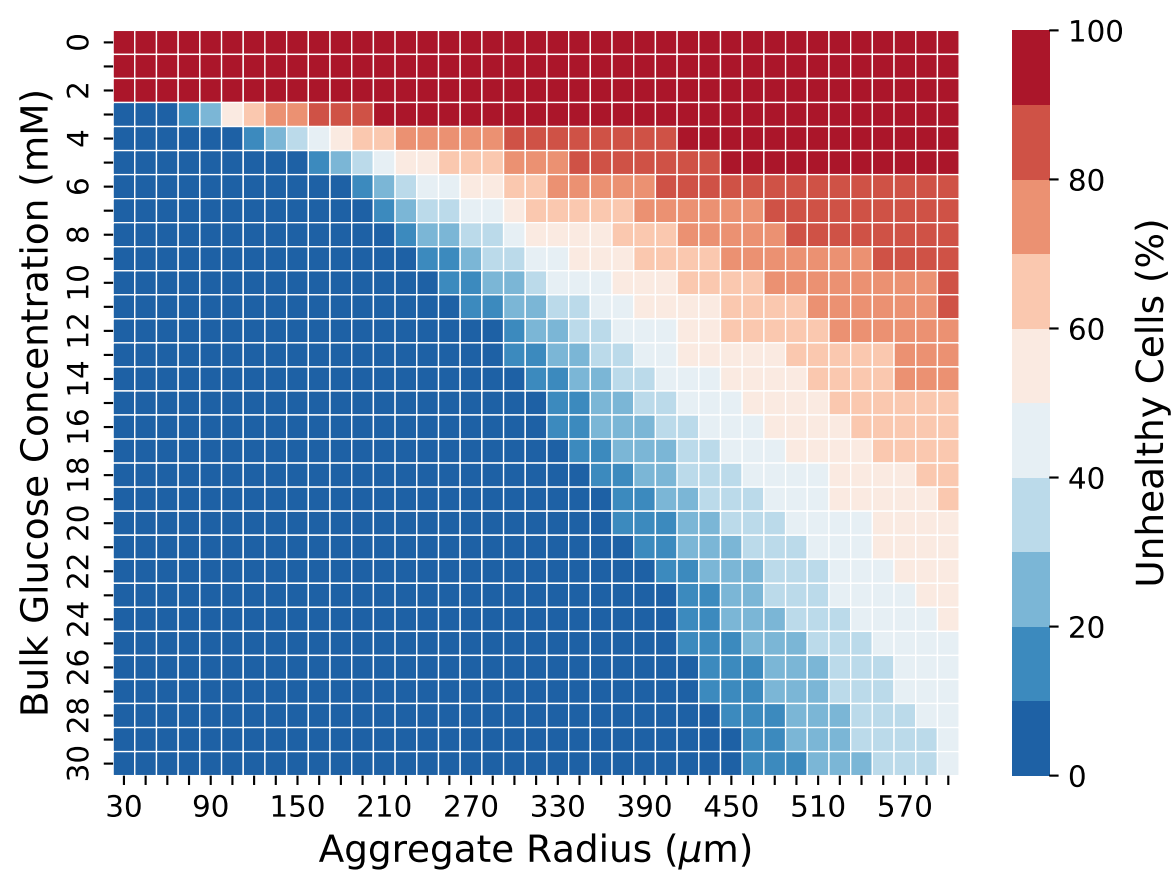

Supplement: Supplementary file 3 — Supplementary Software [file 42003_2023_5653_MOESM3_ESM.zip › MultiScaleModel-master/multi_scale_model/result/unhealthy/Glc-40-2.5-large-font.pdf]

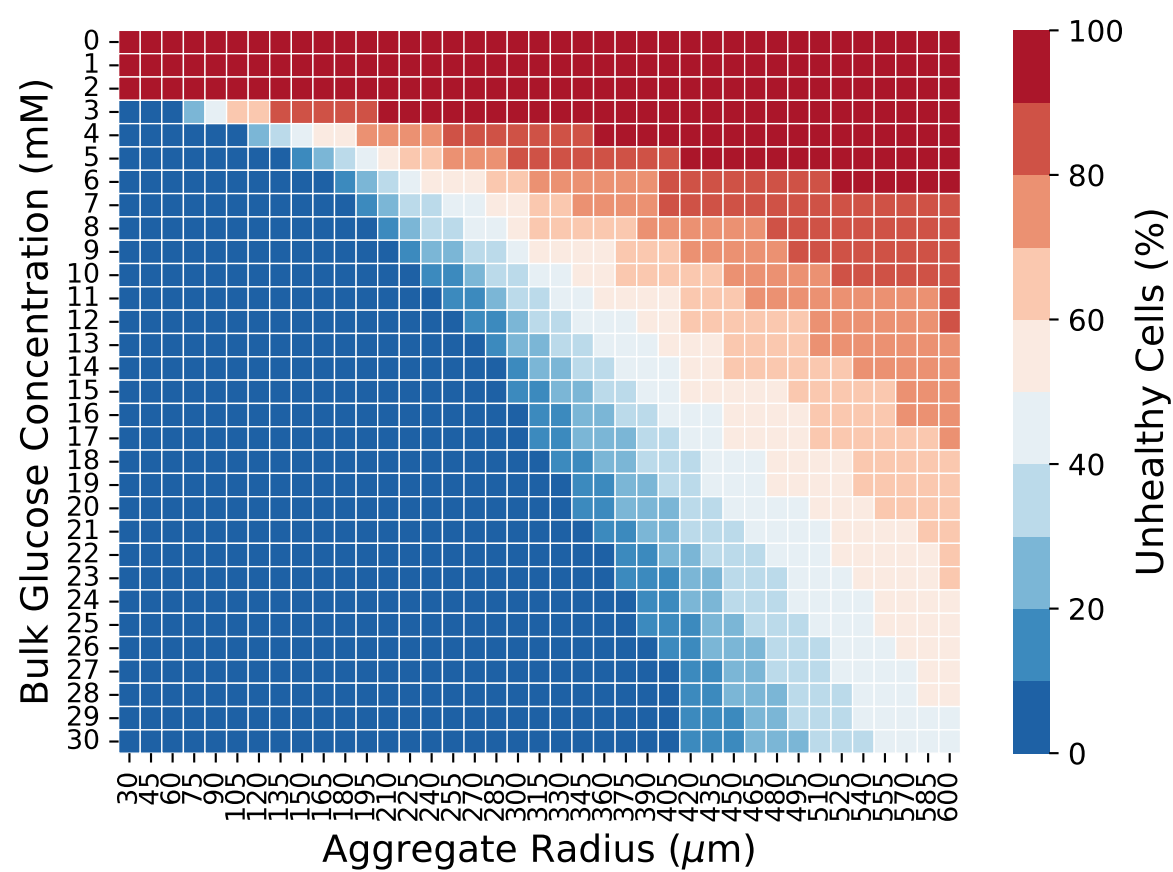

Supplement: Supplementary file 3 — Supplementary Software [file 42003_2023_5653_MOESM3_ESM.zip › MultiScaleModel-master/multi_scale_model/result/unhealthy/Glc-40-2.5.pdf]

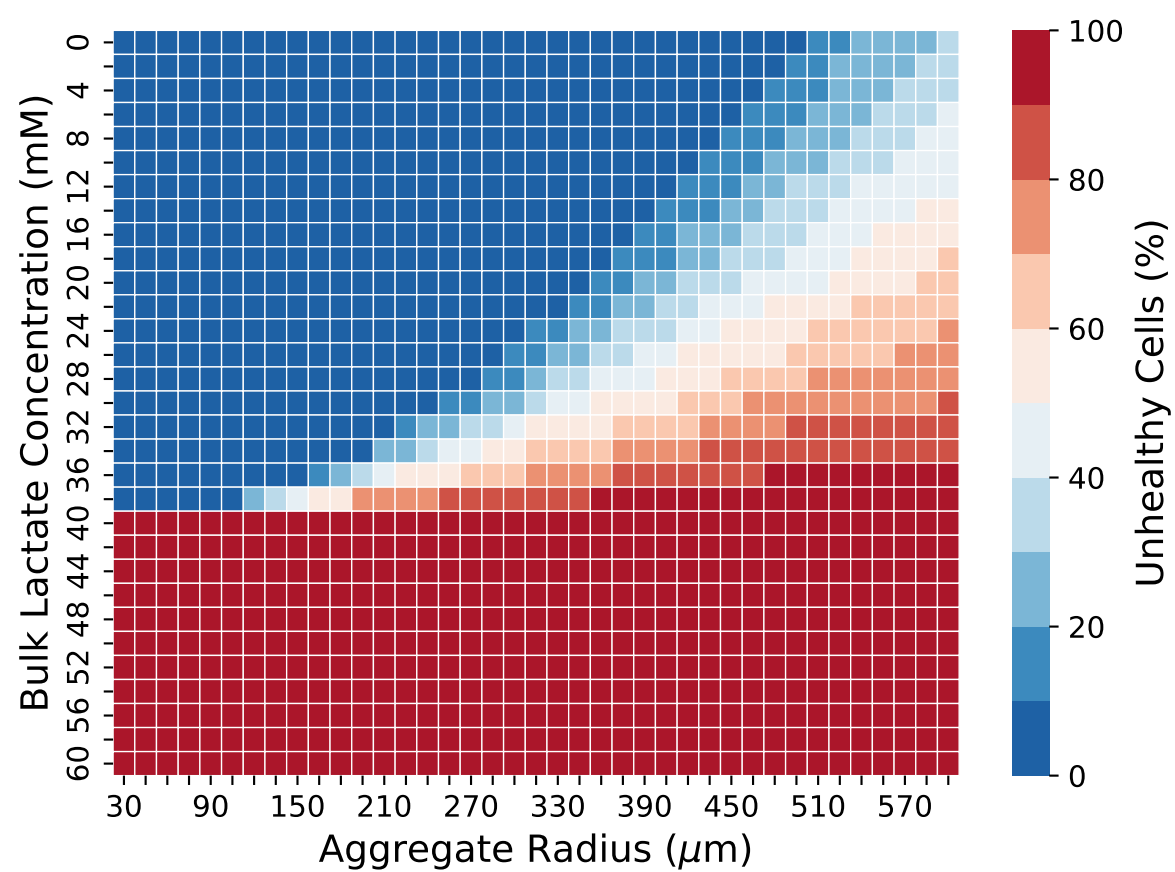

Supplement: Supplementary file 3 — Supplementary Software [file 42003_2023_5653_MOESM3_ESM.zip › MultiScaleModel-master/multi_scale_model/result/unhealthy/Lac-40-2.5-large-font.pdf]

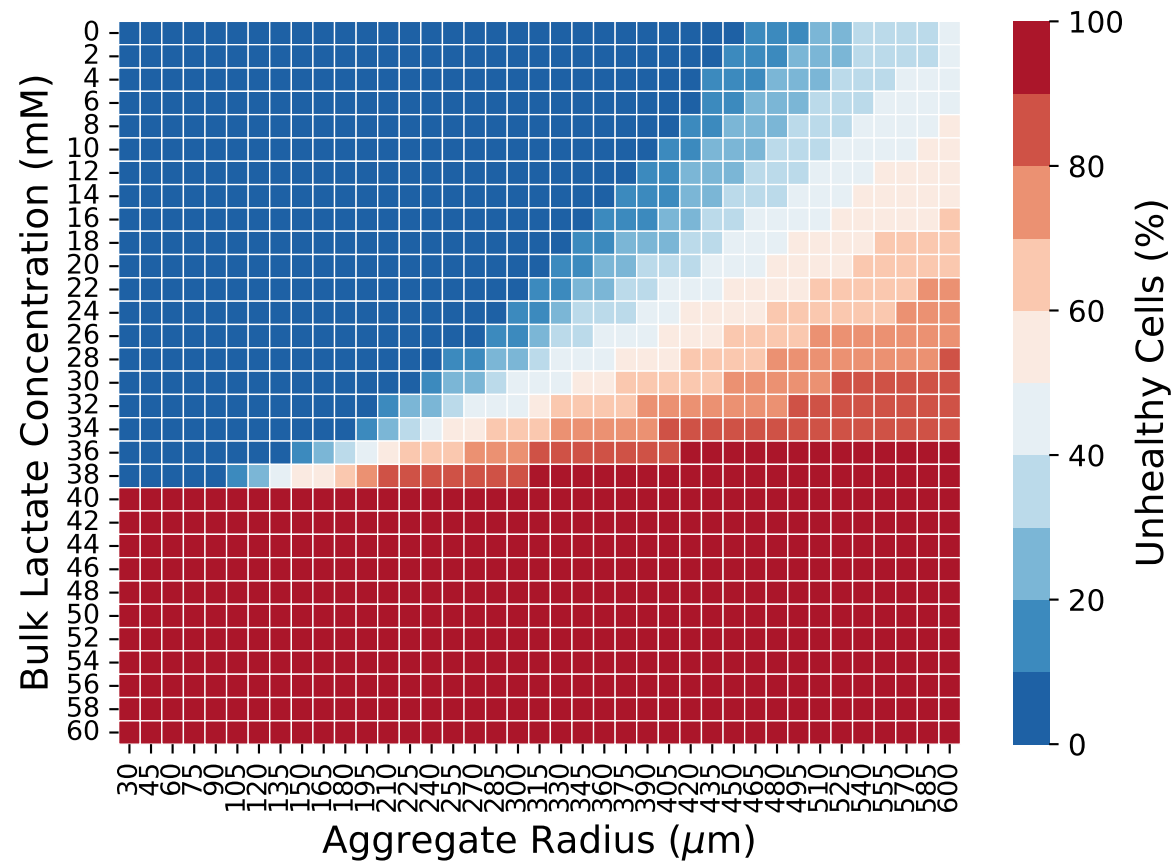

Supplement: Supplementary file 3 — Supplementary Software [file 42003_2023_5653_MOESM3_ESM.zip › MultiScaleModel-master/multi_scale_model/result/unhealthy/Lac-40-2.5.pdf]

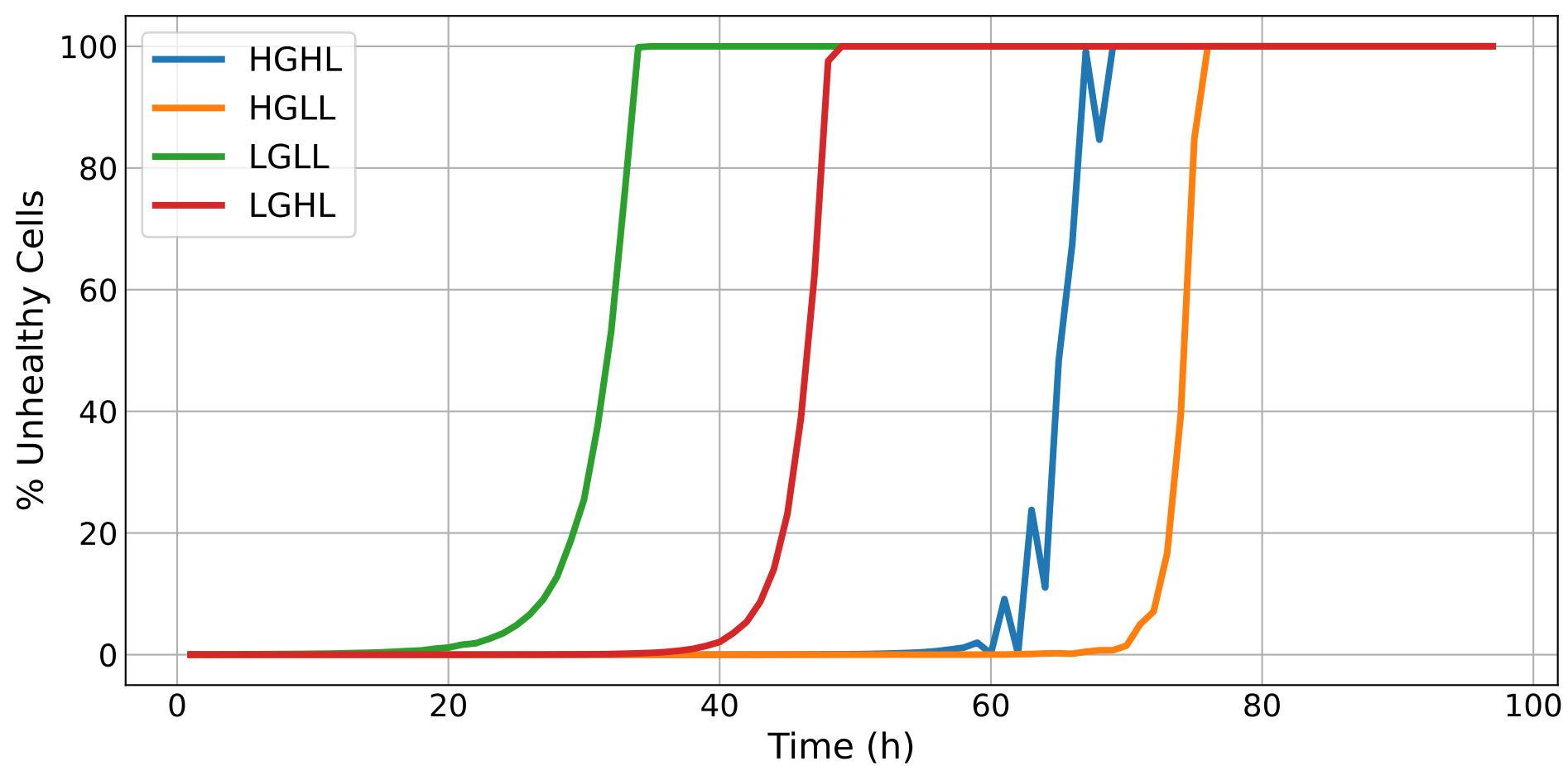

Supplement: Supplementary file 3 — Supplementary Software [file 42003_2023_5653_MOESM3_ESM.zip › MultiScaleModel-master/multi_scale_model/result/unhealthy/batch-unhealthy-time.pdf]

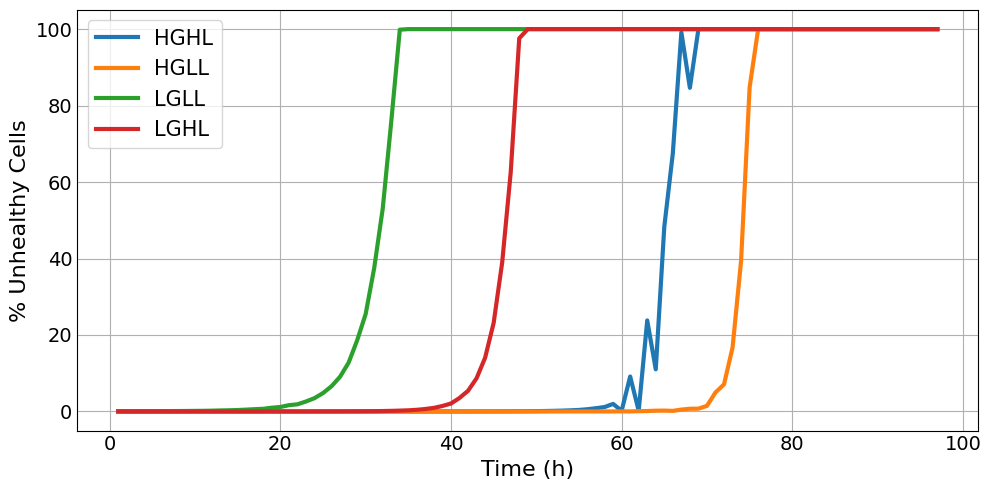

Supplement: Supplementary file 3 — Supplementary Software [file 42003_2023_5653_MOESM3_ESM.zip › MultiScaleModel-master/multi_scale_model/result/unhealthy/batch-unhealthy-time.png]

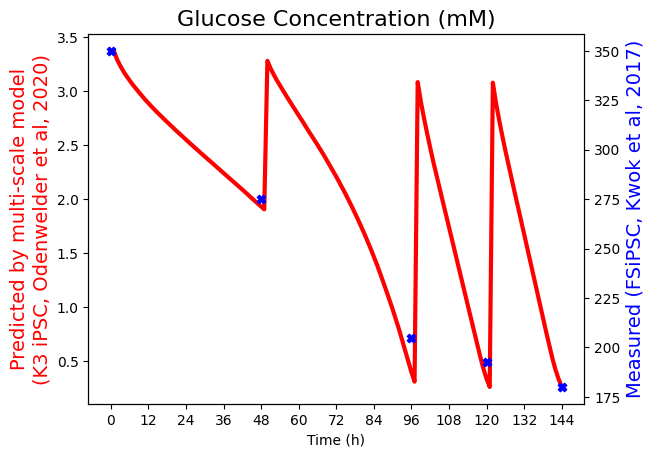

Supplement: Supplementary file 3 — Supplementary Software [file 42003_2023_5653_MOESM3_ESM.zip › MultiScaleModel-master/multi_scale_model/result/validation-Kwok2017/HGLL-43.png]

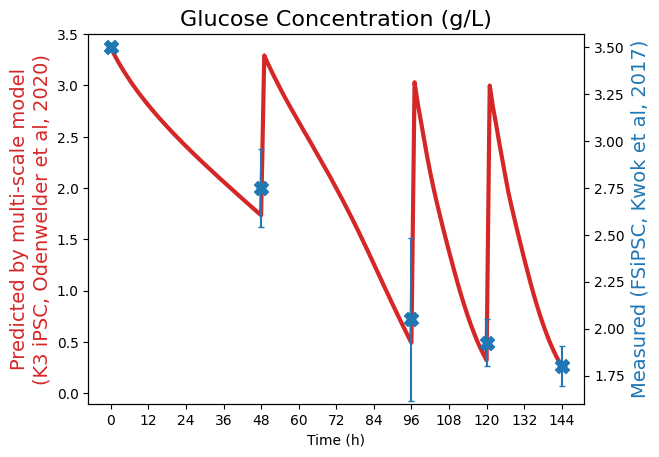

Supplement: Supplementary file 3 — Supplementary Software [file 42003_2023_5653_MOESM3_ESM.zip › MultiScaleModel-master/multi_scale_model/result/validation-Kwok2017/HGLL-GLC-with-err.png]

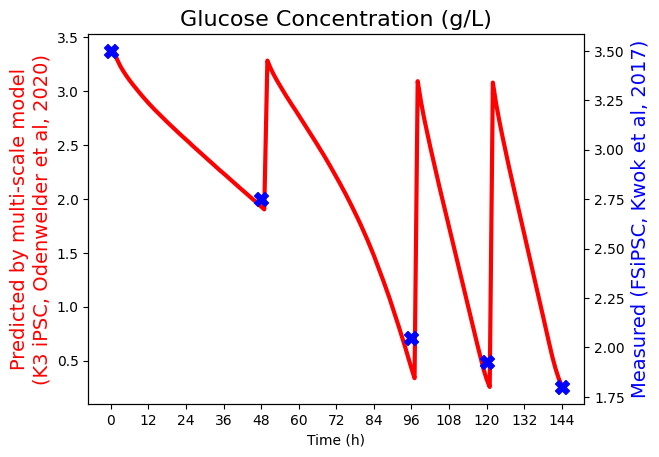

Supplement: Supplementary file 3 — Supplementary Software [file 42003_2023_5653_MOESM3_ESM.zip › MultiScaleModel-master/multi_scale_model/result/validation-Kwok2017/HGLL-GLC.png]

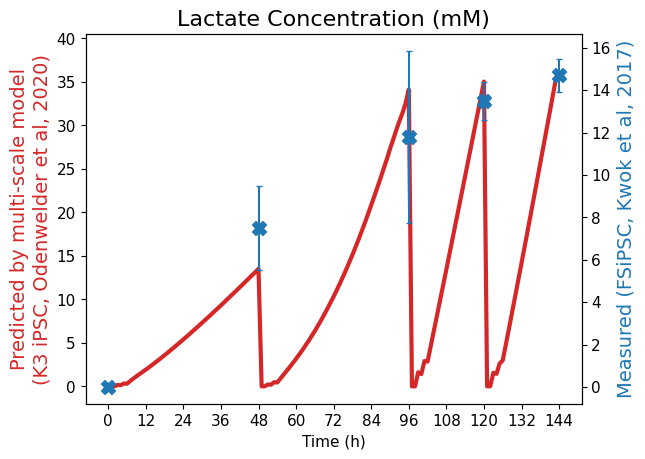

Supplement: Supplementary file 3 — Supplementary Software [file 42003_2023_5653_MOESM3_ESM.zip › MultiScaleModel-master/multi_scale_model/result/validation-Kwok2017/HGLL-Lac-with-err.png]

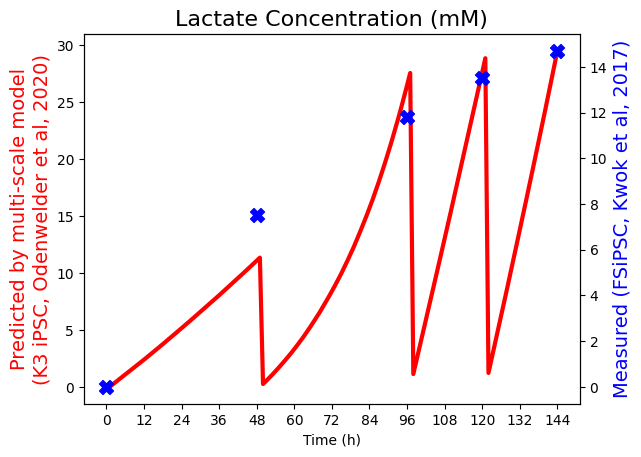

Supplement: Supplementary file 3 — Supplementary Software [file 42003_2023_5653_MOESM3_ESM.zip › MultiScaleModel-master/multi_scale_model/result/validation-Kwok2017/HGLL-Lac.png]

Fraction of Average Flux Rate  
of different Metabolic Pathways

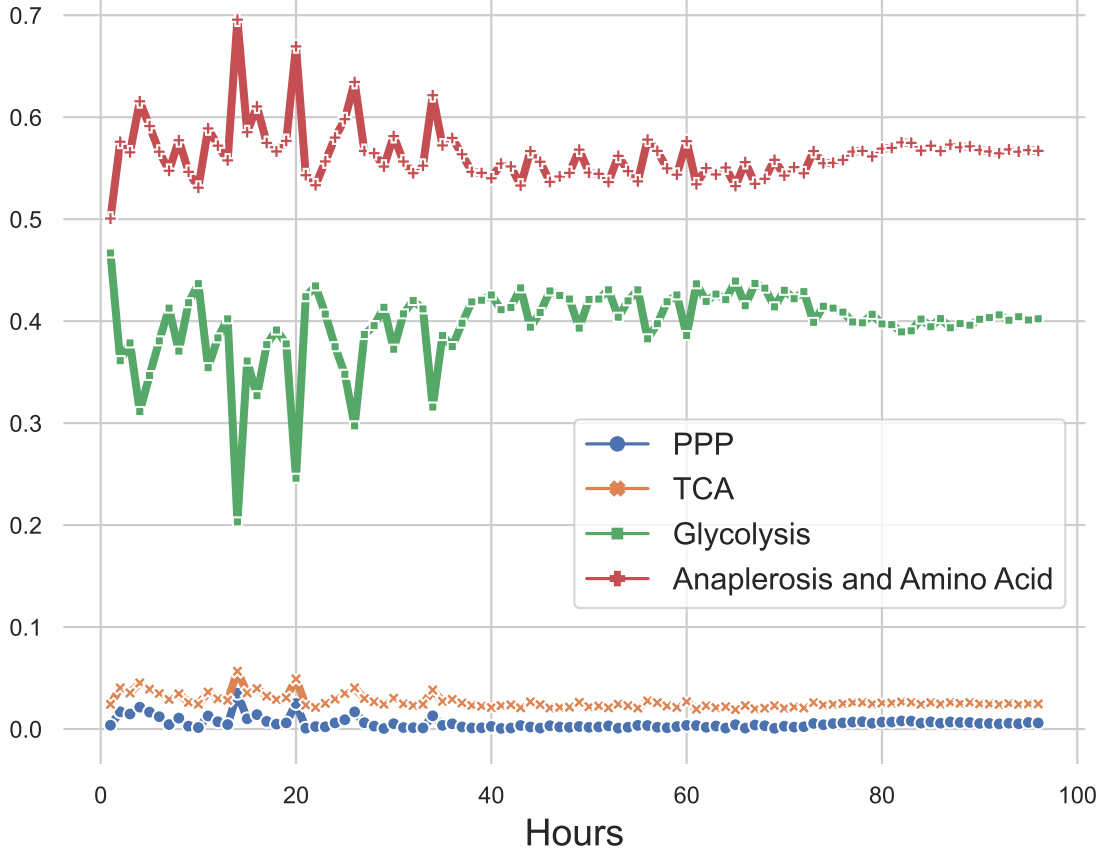

Supplement: Supplementary file 3 — Supplementary Software [file 42003_2023_5653_MOESM3_ESM.zip › MultiScaleModel-master/multi_scale_model/result/variance-component-analysis/expectation-decomposition-with-time.pdf]

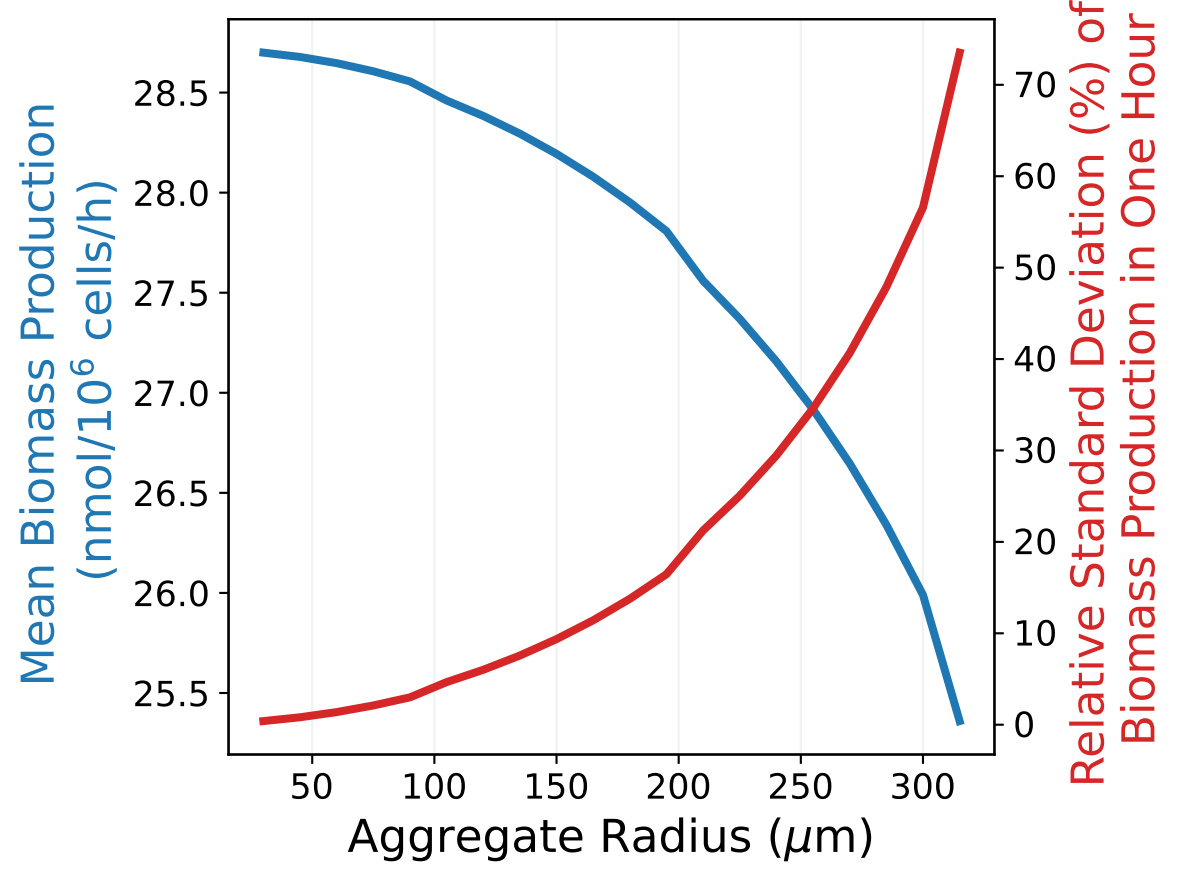

Supplement: Supplementary file 3 — Supplementary Software [file 42003_2023_5653_MOESM3_ESM.zip › MultiScaleModel-master/multi_scale_model/result/variance-component-analysis/large-mean-variance-24.pdf]

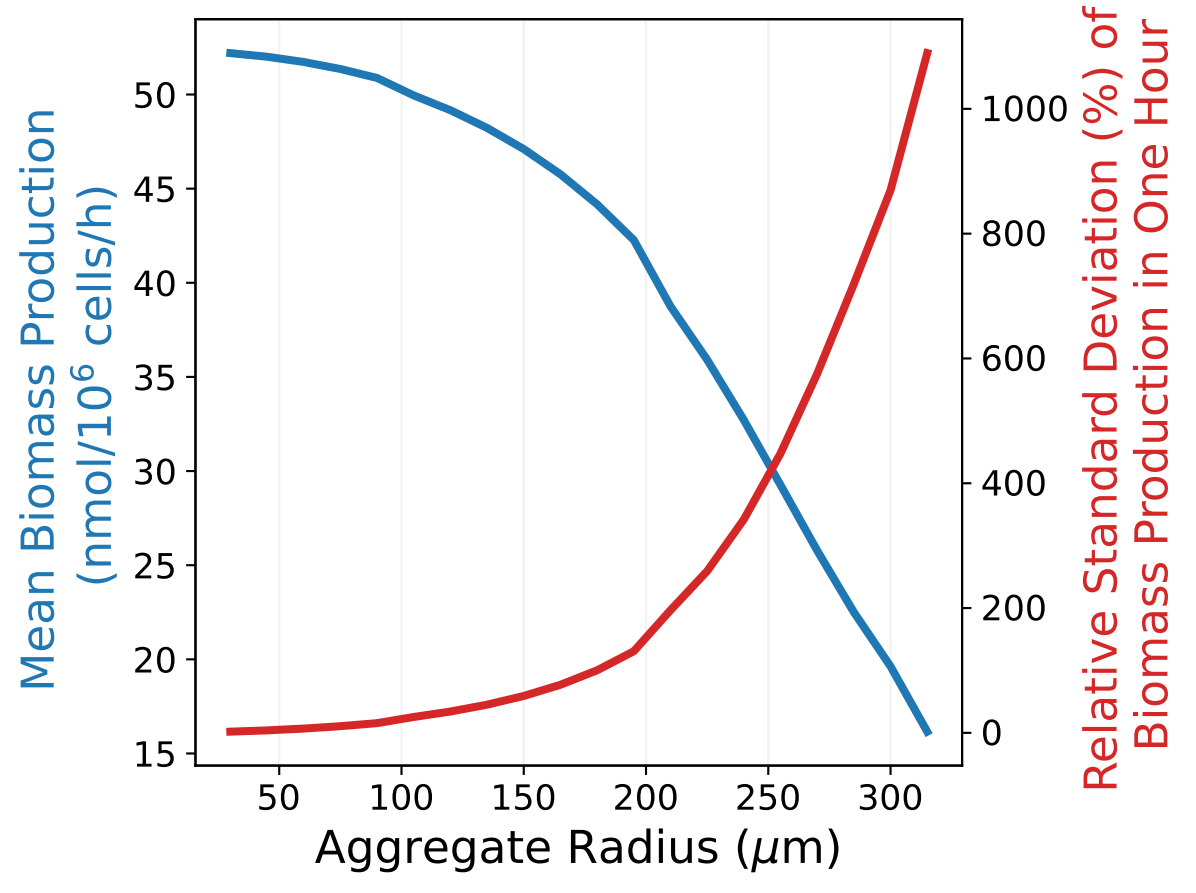

Supplement: Supplementary file 3 — Supplementary Software [file 42003_2023_5653_MOESM3_ESM.zip › MultiScaleModel-master/multi_scale_model/result/variance-component-analysis/large-mean-variance-48.pdf]

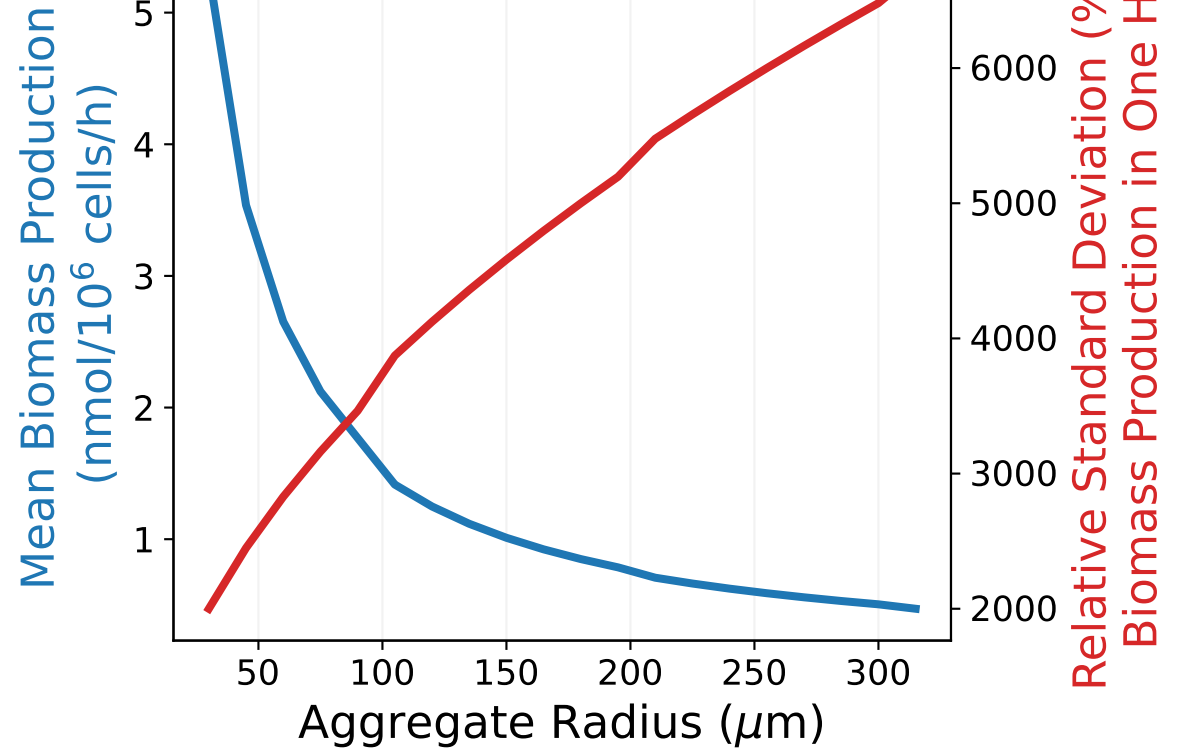

Supplement: Supplementary file 3 — Supplementary Software [file 42003_2023_5653_MOESM3_ESM.zip › MultiScaleModel-master/multi_scale_model/result/variance-component-analysis/large-mean-variance-72.pdf]

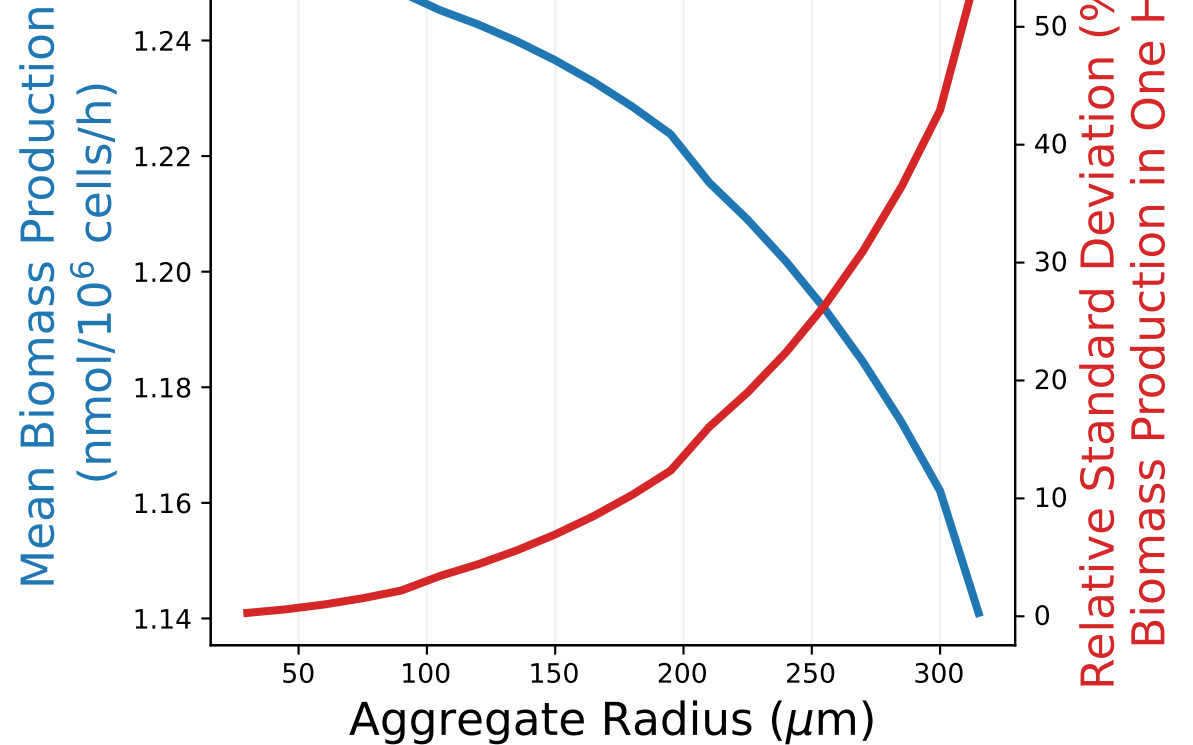

Supplement: Supplementary file 3 — Supplementary Software [file 42003_2023_5653_MOESM3_ESM.zip › MultiScaleModel-master/multi_scale_model/result/variance-component-analysis/mean-variance-0.pdf]

Culture Hour: 12

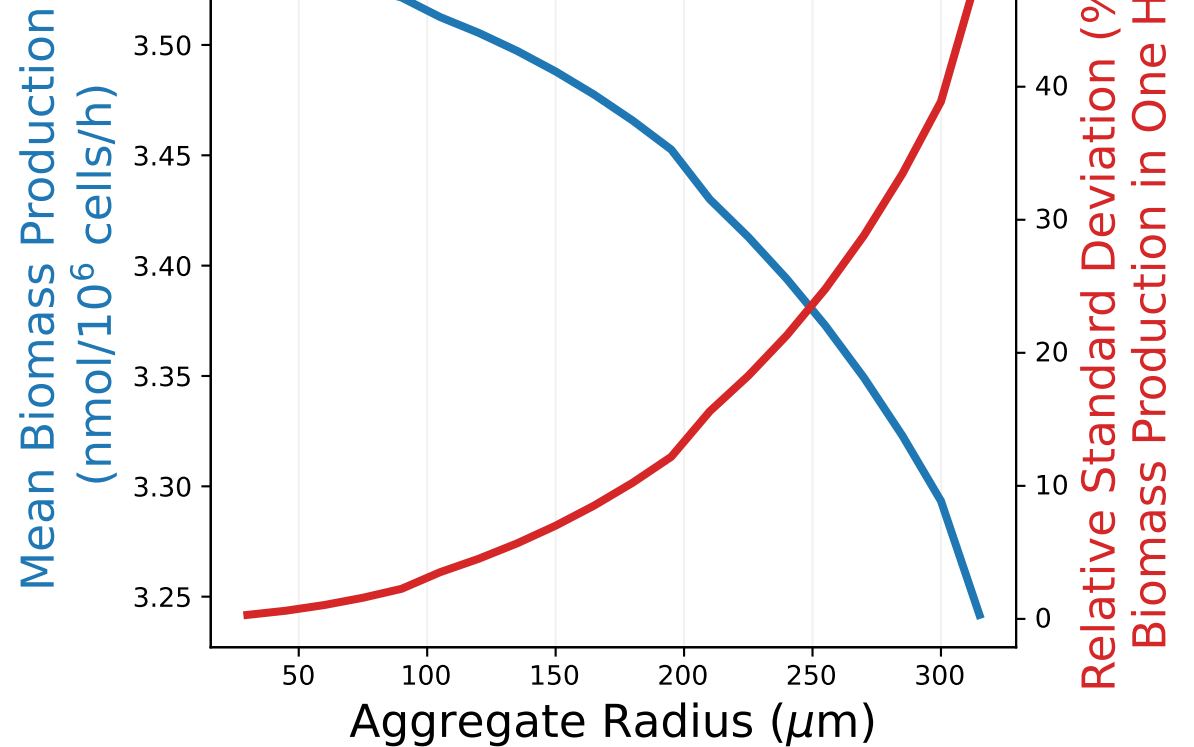

Supplement: Supplementary file 3 — Supplementary Software [file 42003_2023_5653_MOESM3_ESM.zip › MultiScaleModel-master/multi_scale_model/result/variance-component-analysis/mean-variance-12.pdf]
