# Supplementary figures and images for: Stochastic biological system-of-systems modelling for iPSC culture (part 1 of 2)
Source: Commun Biol. 2024 Jan 8;7:39. doi: 10.1038/s42003-023-05653-w (PMC10774284; doi:10.1038/s42003-023-05653-w)

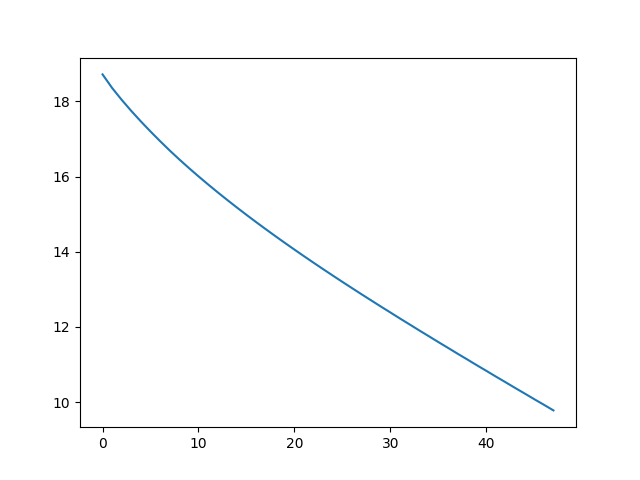

Supplement: Supplementary file 3 — Supplementary Software [file 42003_2023_5653_MOESM3_ESM.zip › MultiScaleModel-master/asset/glc.png]

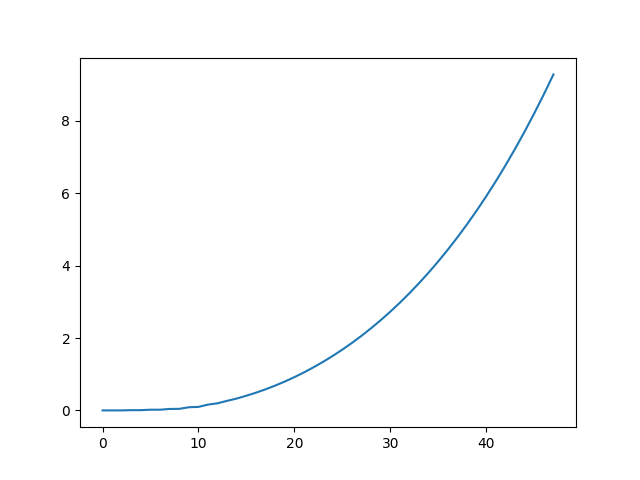

Supplement: Supplementary file 3 — Supplementary Software [file 42003_2023_5653_MOESM3_ESM.zip › MultiScaleModel-master/asset/lac.png]

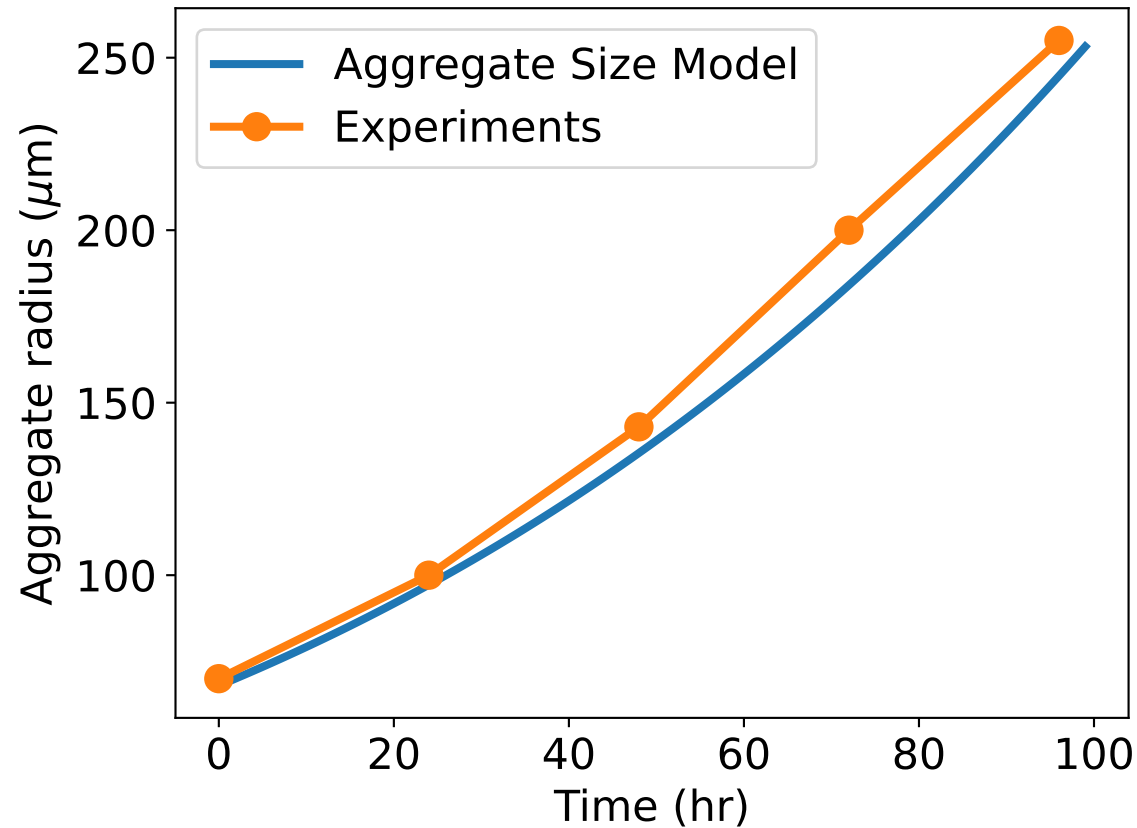

Supplement: Supplementary file 3 — Supplementary Software [file 42003_2023_5653_MOESM3_ESM.zip › MultiScaleModel-master/multi_scale_model/result/PBM/mean_curve.pdf]

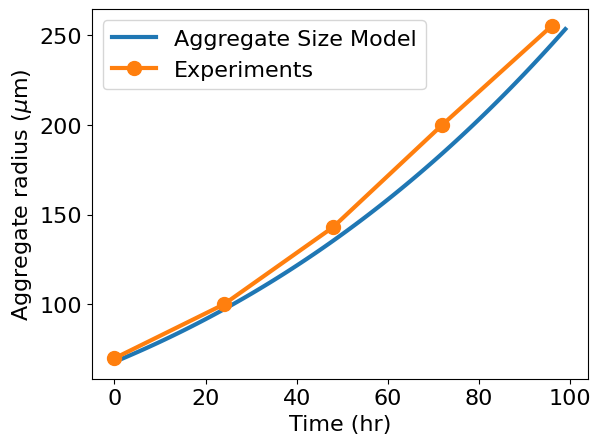

Supplement: Supplementary file 3 — Supplementary Software [file 42003_2023_5653_MOESM3_ESM.zip › MultiScaleModel-master/multi_scale_model/result/PBM/mean_curve.png]

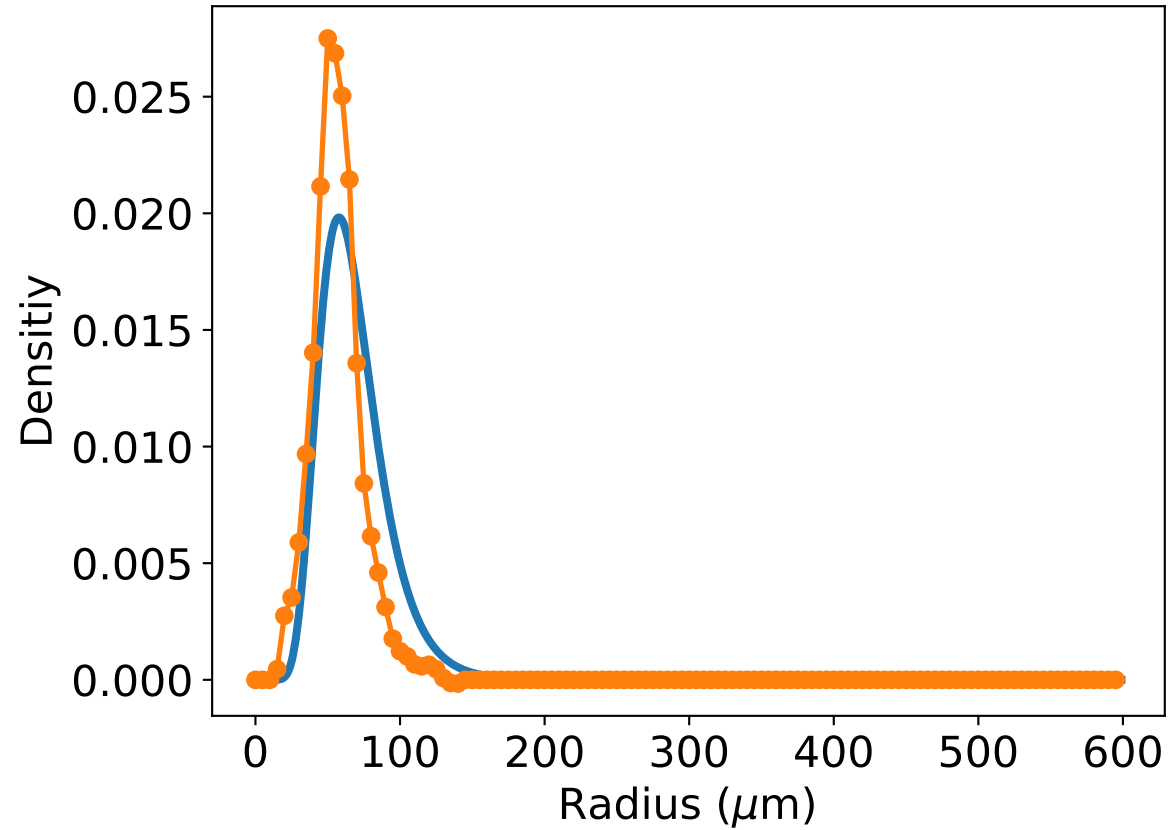

Supplement: Supplementary file 3 — Supplementary Software [file 42003_2023_5653_MOESM3_ESM.zip › MultiScaleModel-master/multi_scale_model/result/PBM/radius-600-hour-0.pdf]

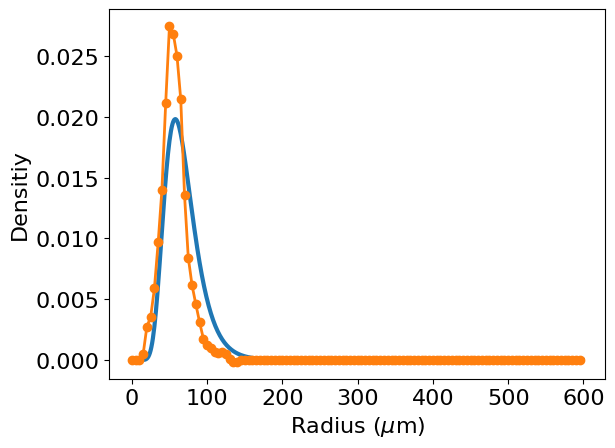

Supplement: Supplementary file 3 — Supplementary Software [file 42003_2023_5653_MOESM3_ESM.zip › MultiScaleModel-master/multi_scale_model/result/PBM/radius-600-hour-0.png]

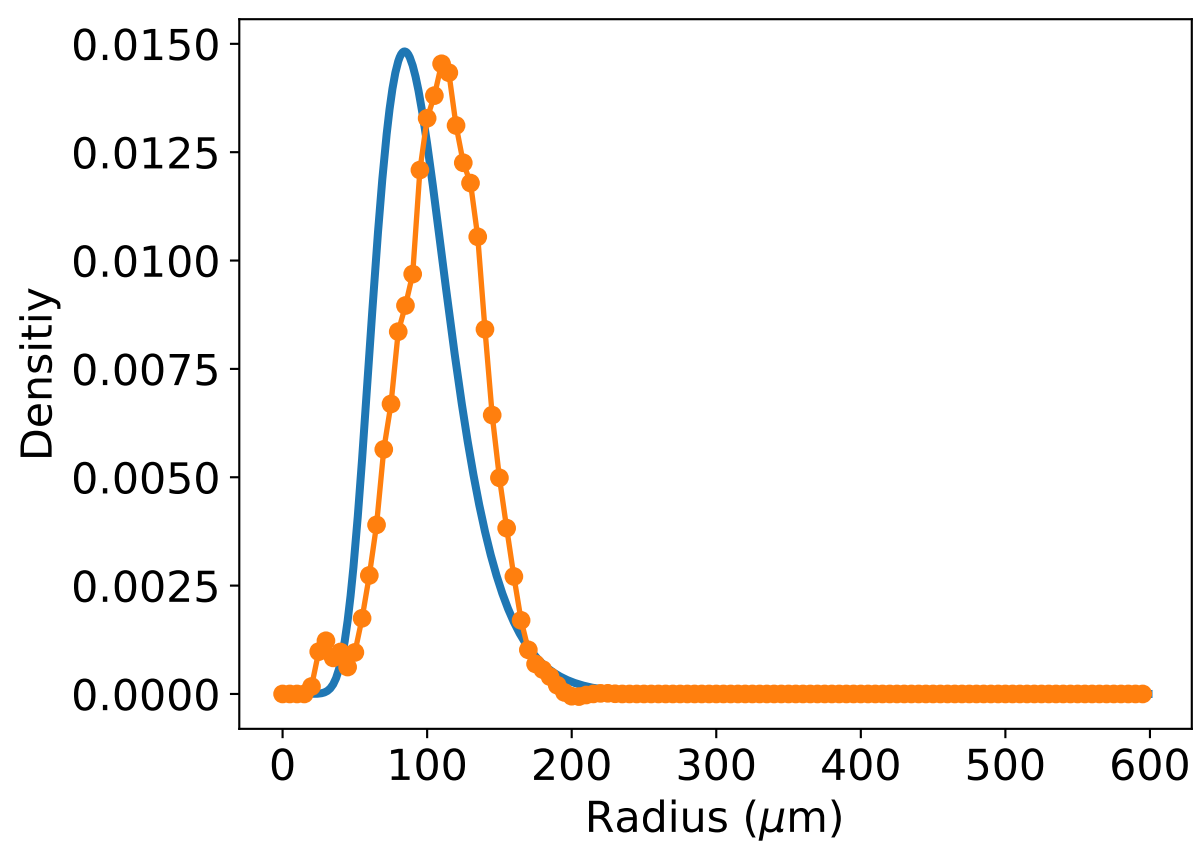

Supplement: Supplementary file 3 — Supplementary Software [file 42003_2023_5653_MOESM3_ESM.zip › MultiScaleModel-master/multi_scale_model/result/PBM/radius-600-hour-24.pdf]

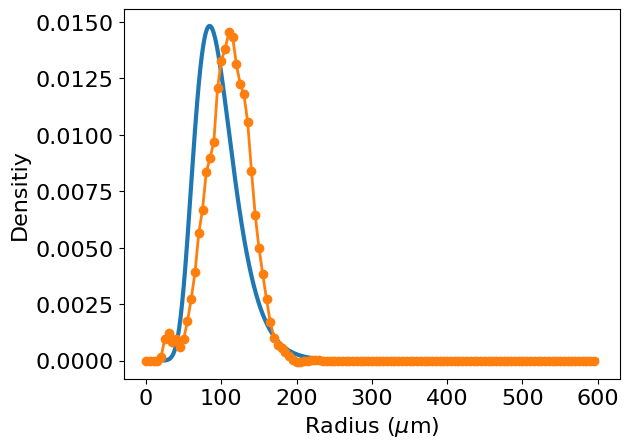

Supplement: Supplementary file 3 — Supplementary Software [file 42003_2023_5653_MOESM3_ESM.zip › MultiScaleModel-master/multi_scale_model/result/PBM/radius-600-hour-24.png]

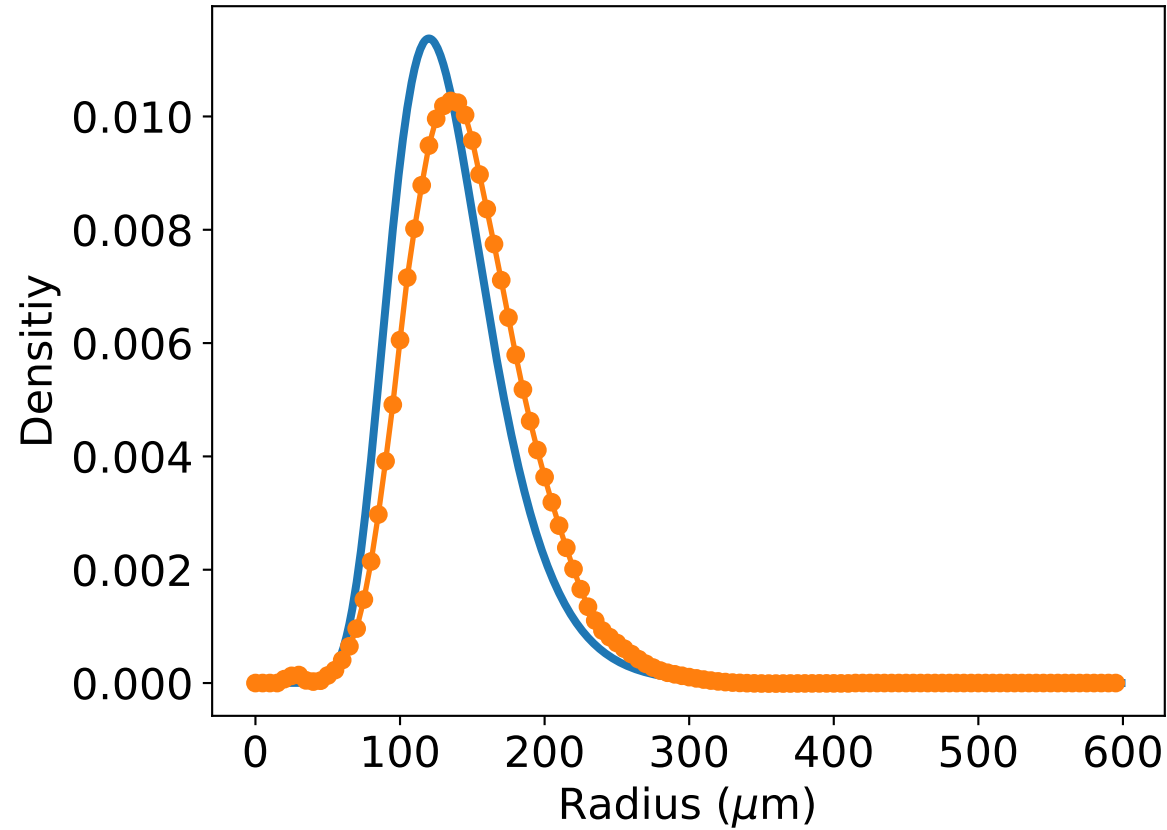

Supplement: Supplementary file 3 — Supplementary Software [file 42003_2023_5653_MOESM3_ESM.zip › MultiScaleModel-master/multi_scale_model/result/PBM/radius-600-hour-48.pdf]

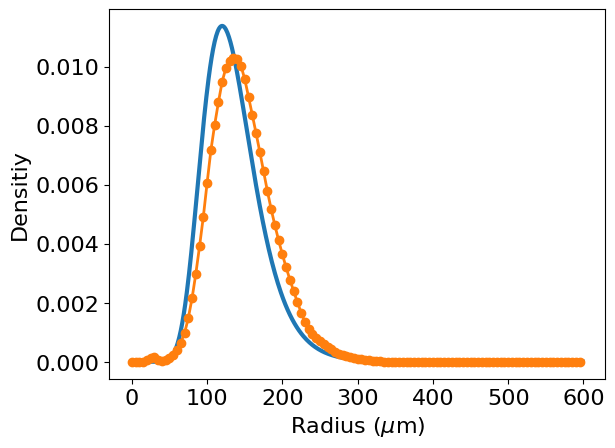

Supplement: Supplementary file 3 — Supplementary Software [file 42003_2023_5653_MOESM3_ESM.zip › MultiScaleModel-master/multi_scale_model/result/PBM/radius-600-hour-48.png]

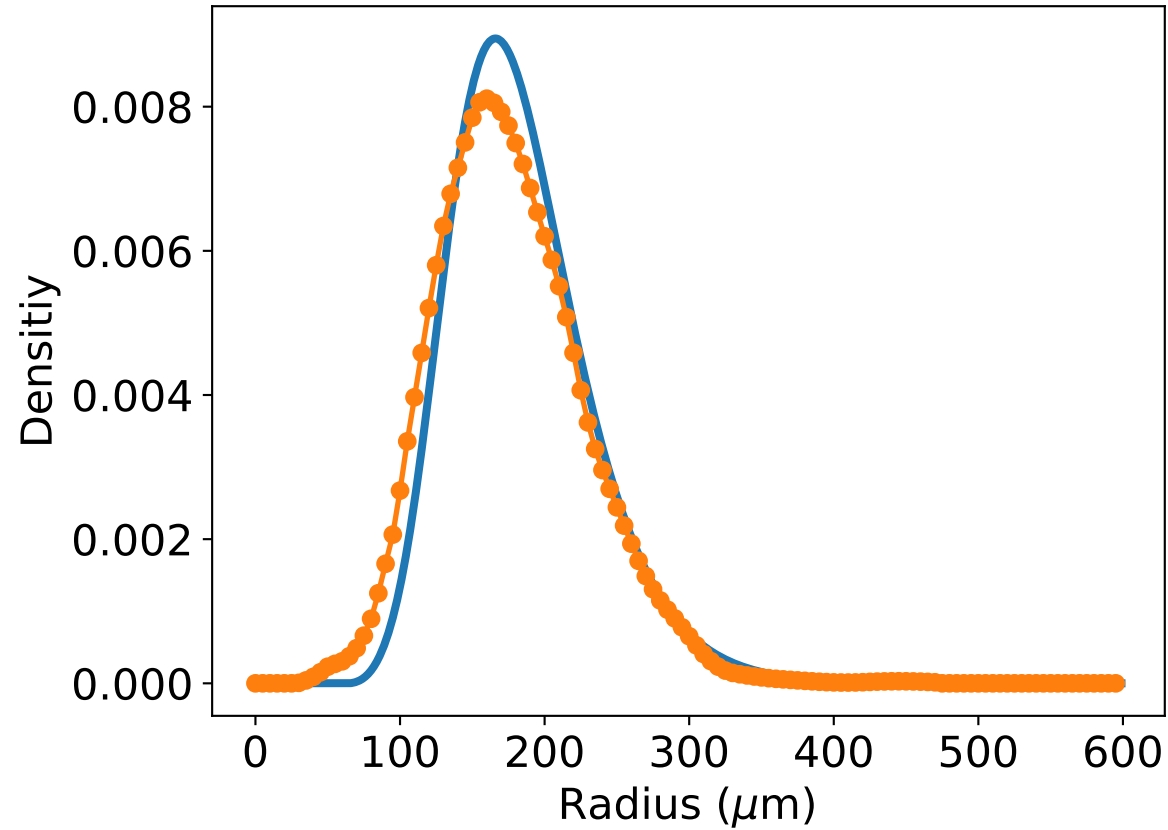

Supplement: Supplementary file 3 — Supplementary Software [file 42003_2023_5653_MOESM3_ESM.zip › MultiScaleModel-master/multi_scale_model/result/PBM/radius-600-hour-72.pdf]

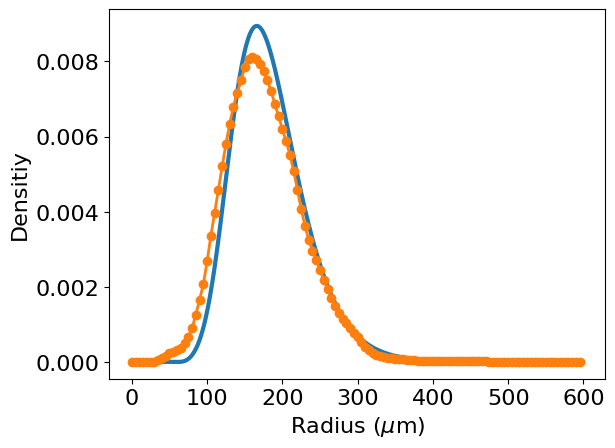

Supplement: Supplementary file 3 — Supplementary Software [file 42003_2023_5653_MOESM3_ESM.zip › MultiScaleModel-master/multi_scale_model/result/PBM/radius-600-hour-72.png]

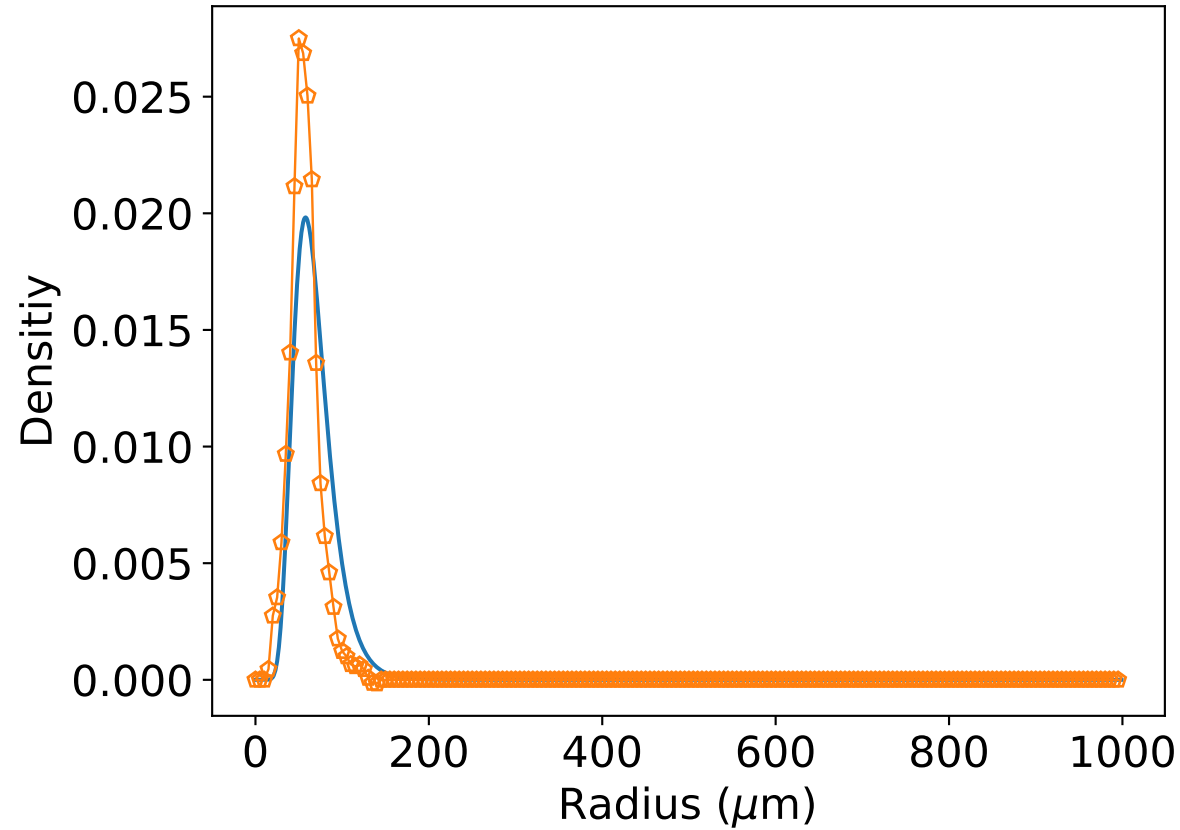

Supplement: Supplementary file 3 — Supplementary Software [file 42003_2023_5653_MOESM3_ESM.zip › MultiScaleModel-master/multi_scale_model/result/hour-0.pdf]

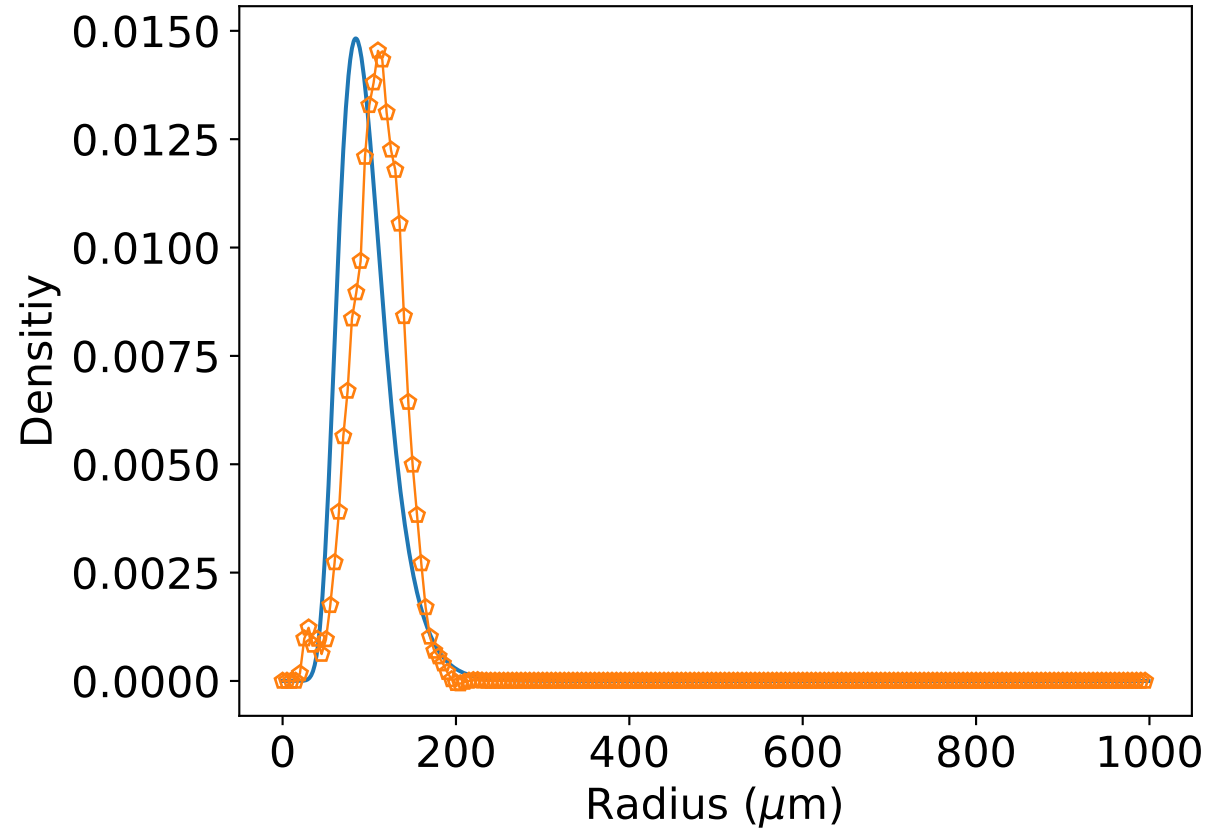

Supplement: Supplementary file 3 — Supplementary Software [file 42003_2023_5653_MOESM3_ESM.zip › MultiScaleModel-master/multi_scale_model/result/hour-24.pdf]

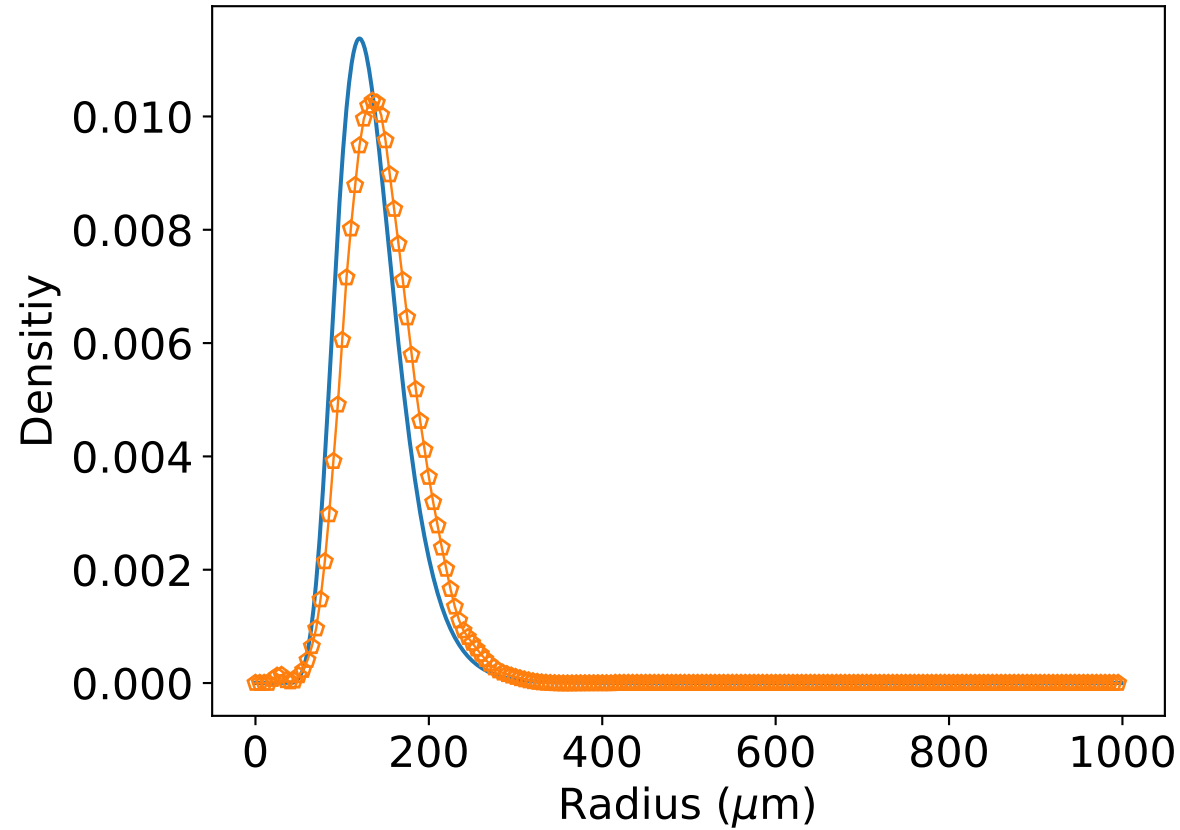

Supplement: Supplementary file 3 — Supplementary Software [file 42003_2023_5653_MOESM3_ESM.zip › MultiScaleModel-master/multi_scale_model/result/hour-48.pdf]

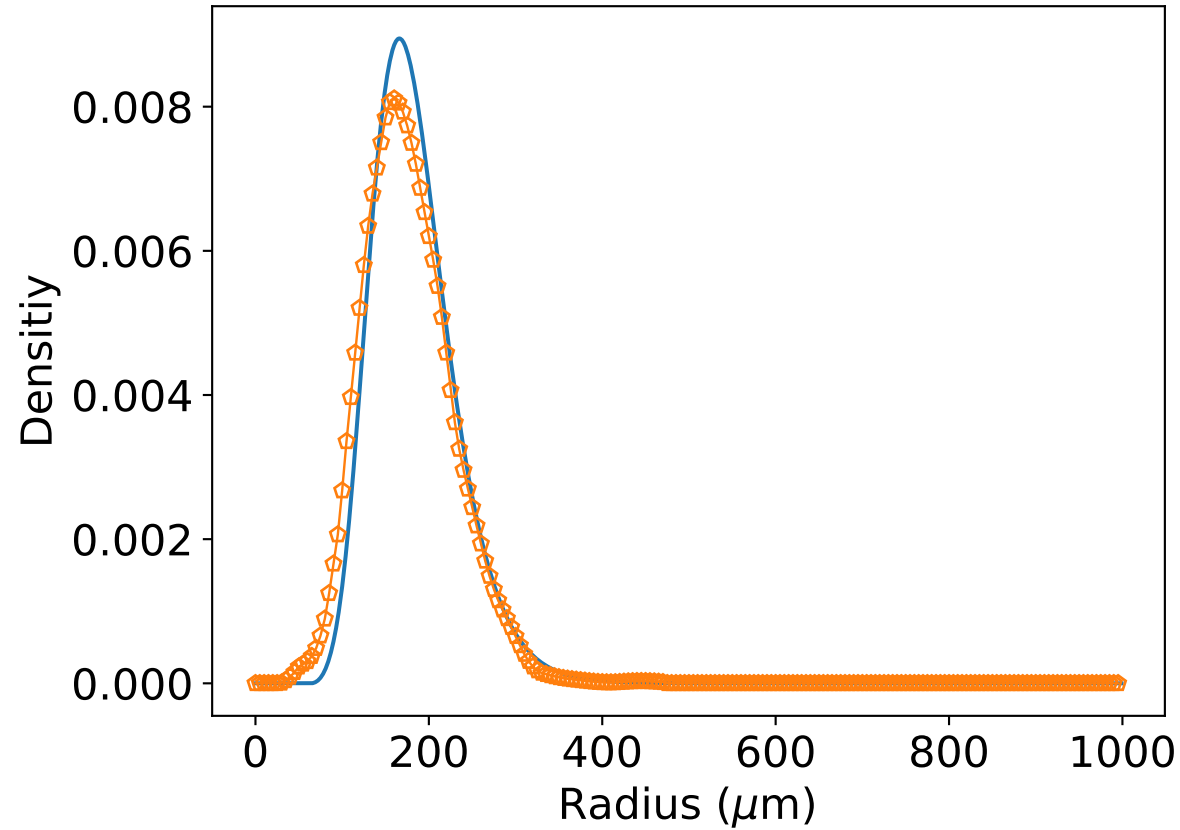

Supplement: Supplementary file 3 — Supplementary Software [file 42003_2023_5653_MOESM3_ESM.zip › MultiScaleModel-master/multi_scale_model/result/hour-72.pdf]

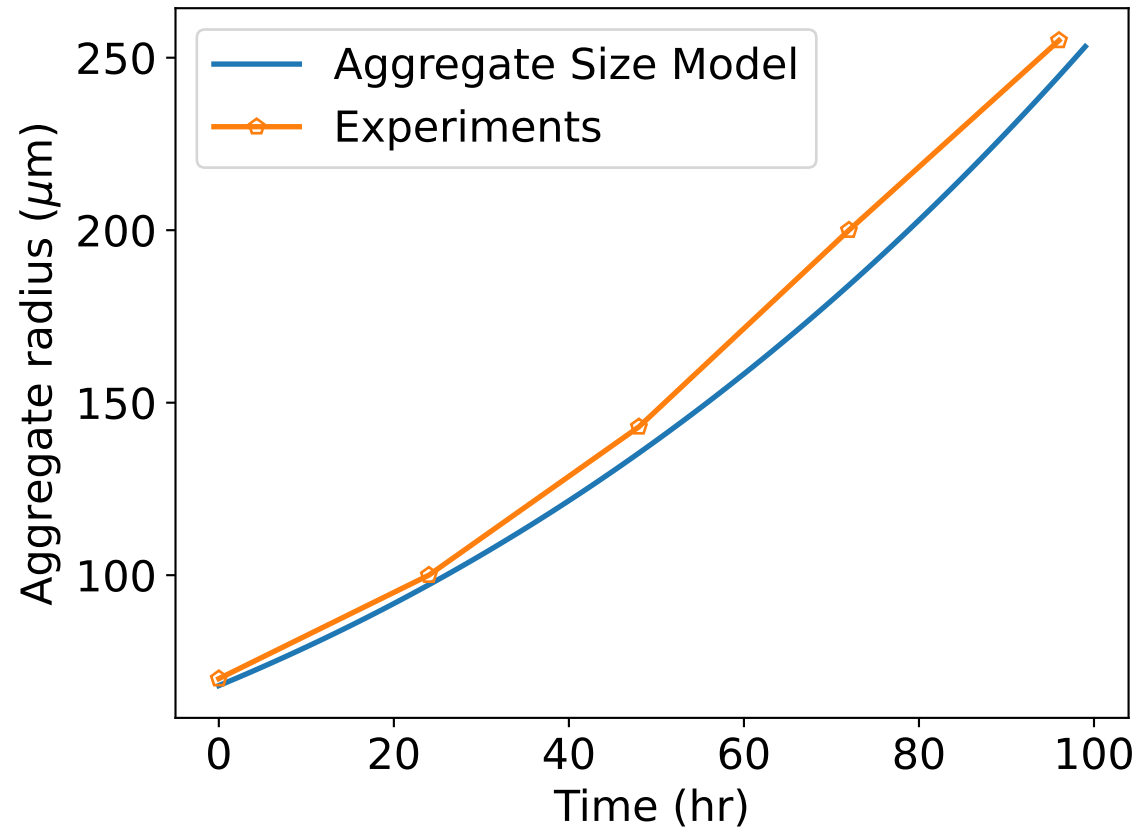

Supplement: Supplementary file 3 — Supplementary Software [file 42003_2023_5653_MOESM3_ESM.zip › MultiScaleModel-master/multi_scale_model/result/mean_curve.pdf]

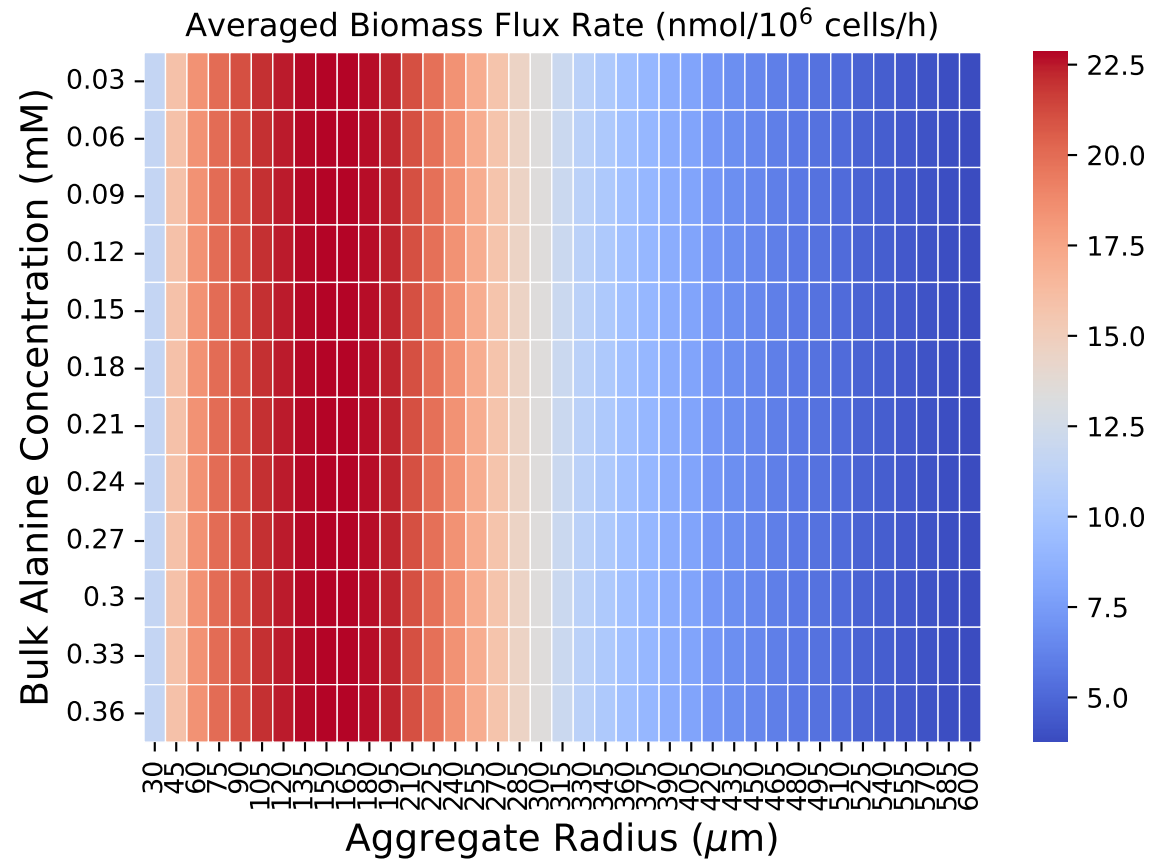

Supplement: Supplementary file 3 — Supplementary Software [file 42003_2023_5653_MOESM3_ESM.zip › MultiScaleModel-master/multi_scale_model/result/optimal_size/Biomass-Alanine.pdf]

Averaged Biomass Flux Rate (nmol/10<sup>6</sup> cells/h)

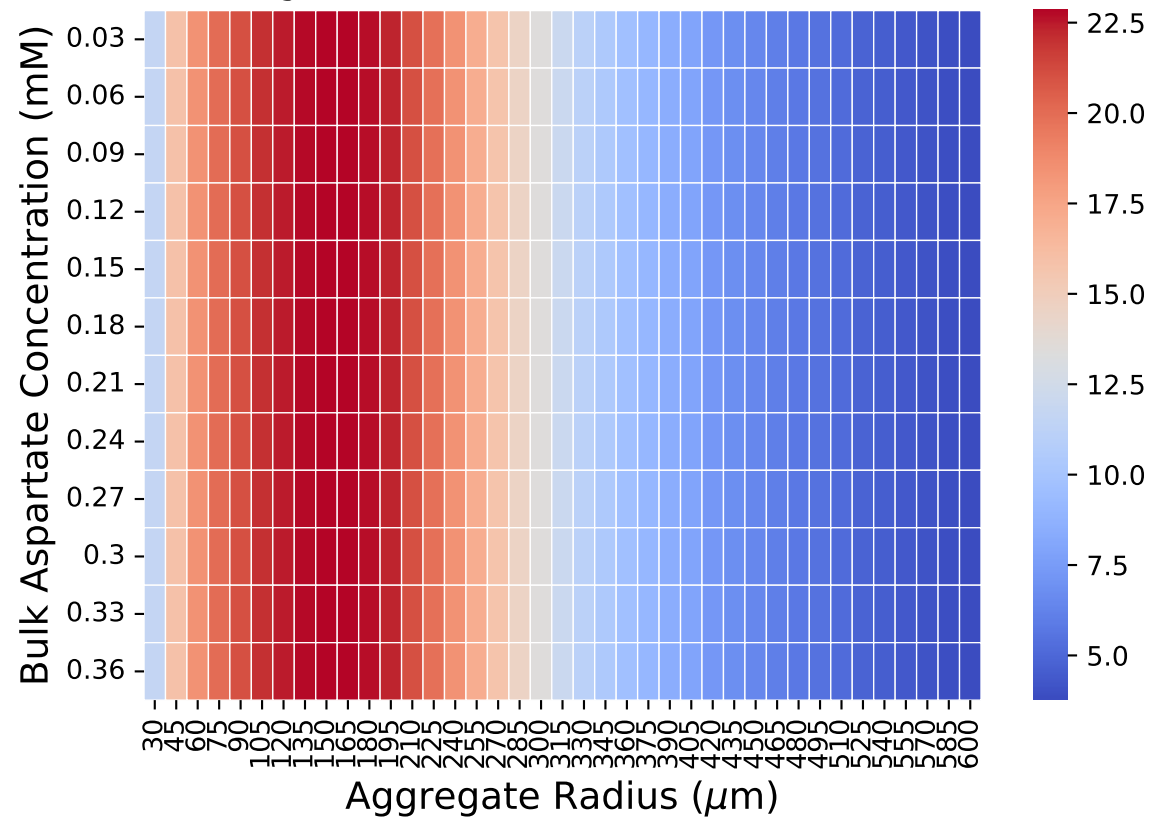

Supplement: Supplementary file 3 — Supplementary Software [file 42003_2023_5653_MOESM3_ESM.zip › MultiScaleModel-master/multi_scale_model/result/optimal_size/Biomass-Aspartate.pdf]

Averaged Extracellular Metabolite Concentrations  
(after standardization)

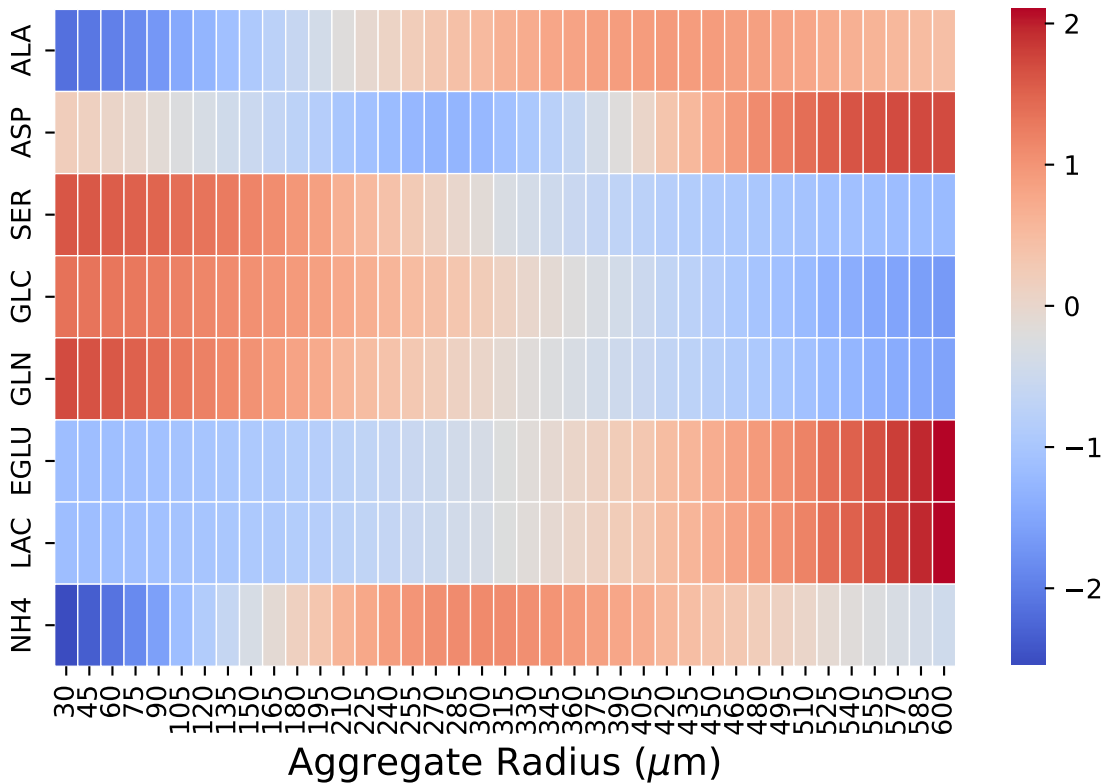

Supplement: Supplementary file 3 — Supplementary Software [file 42003_2023_5653_MOESM3_ESM.zip › MultiScaleModel-master/multi_scale_model/result/optimal_size/Biomass-metabolites-ala-0.05.pdf]

Averaged Extracellular Metabolite Concentrations  
(after standardization)

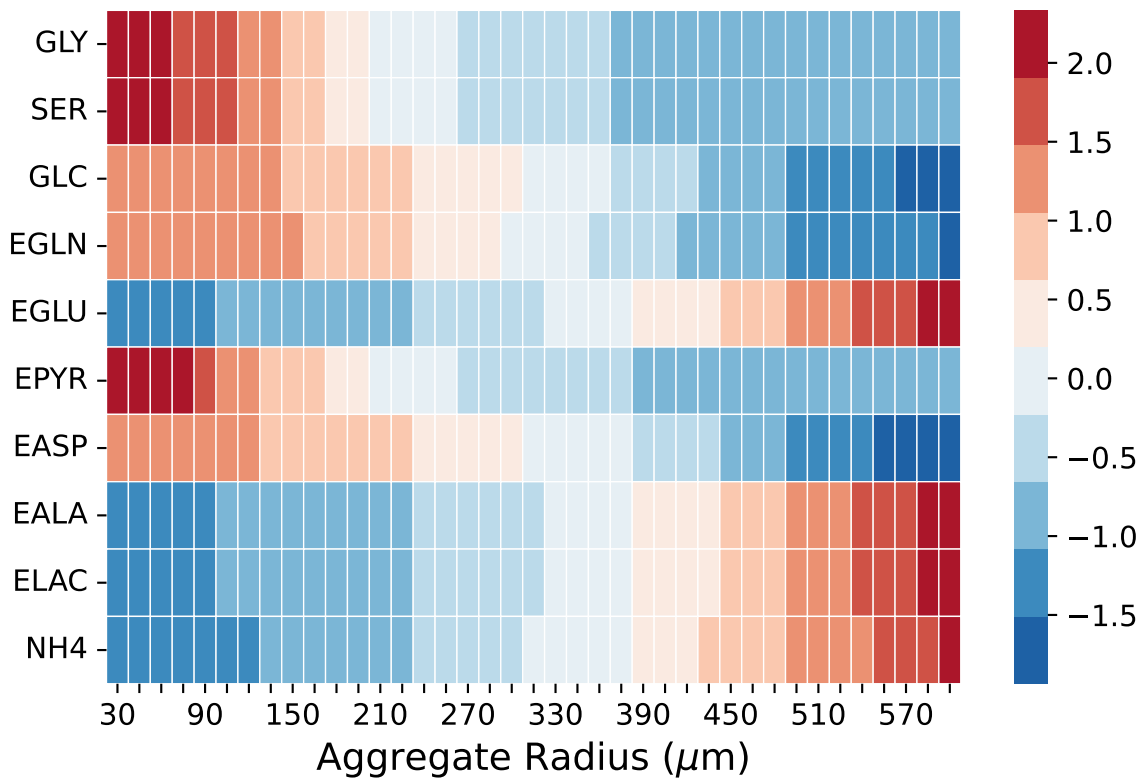

Supplement: Supplementary file 3 — Supplementary Software [file 42003_2023_5653_MOESM3_ESM.zip › MultiScaleModel-master/multi_scale_model/result/optimal_size/Biomass-metabolites-ala-0.1.pdf]

Averaged Extracellular Metabolite Concentrations  
(after standardization)

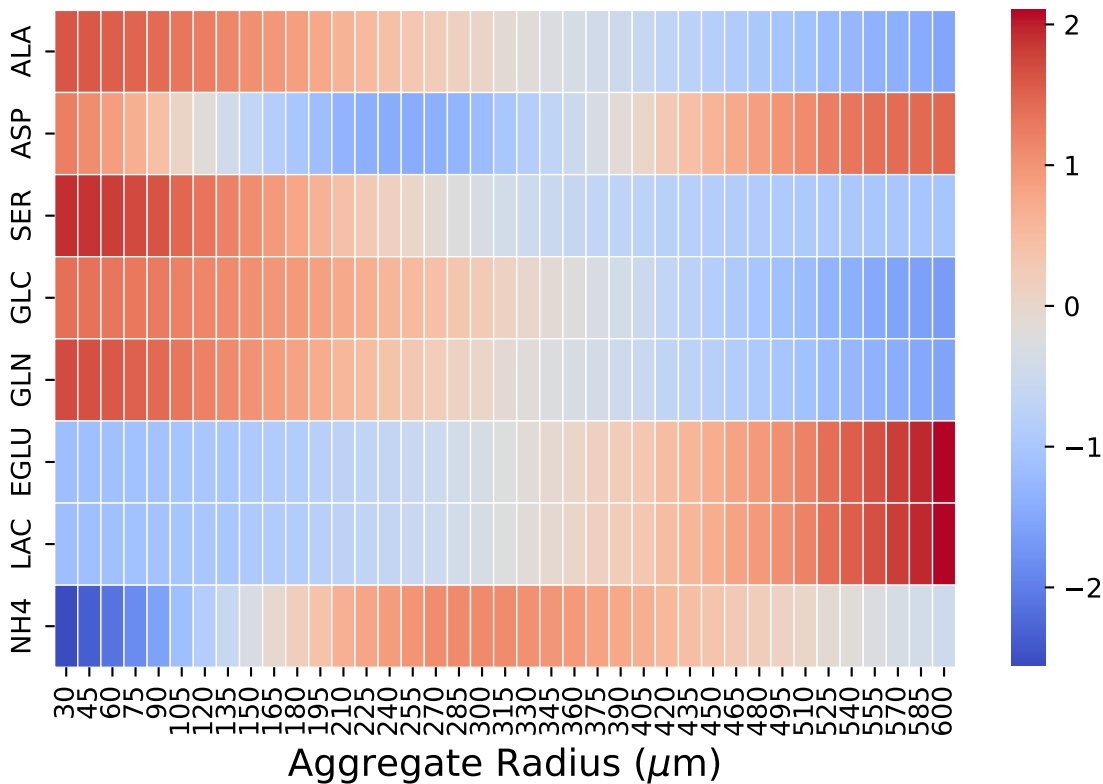

Supplement: Supplementary file 3 — Supplementary Software [file 42003_2023_5653_MOESM3_ESM.zip › MultiScaleModel-master/multi_scale_model/result/optimal_size/Biomass-metabolites-ala-0.5.pdf]

Averaged Extracellular Metabolite Concentrations  
(after standardization)

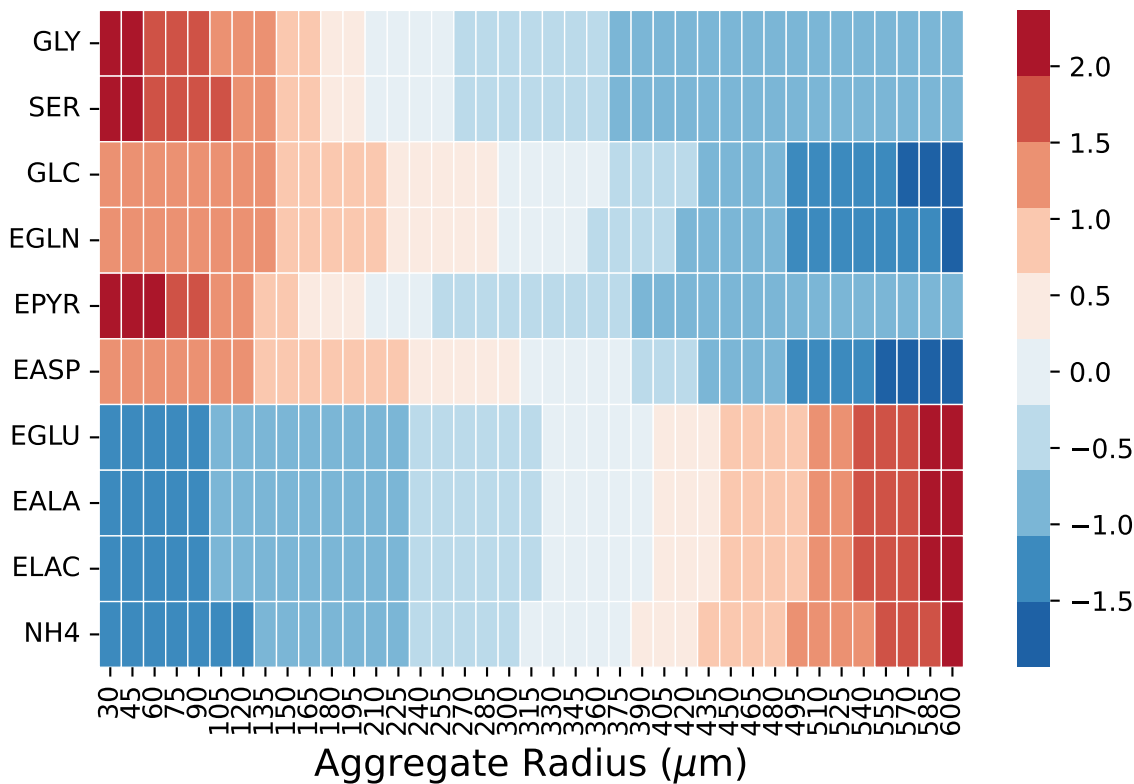

Supplement: Supplementary file 3 — Supplementary Software [file 42003_2023_5653_MOESM3_ESM.zip › MultiScaleModel-master/multi_scale_model/result/optimal_size/Biomass-metabolites-ordered-ala-0.1.pdf]

Average Flux Rates (after standardization)

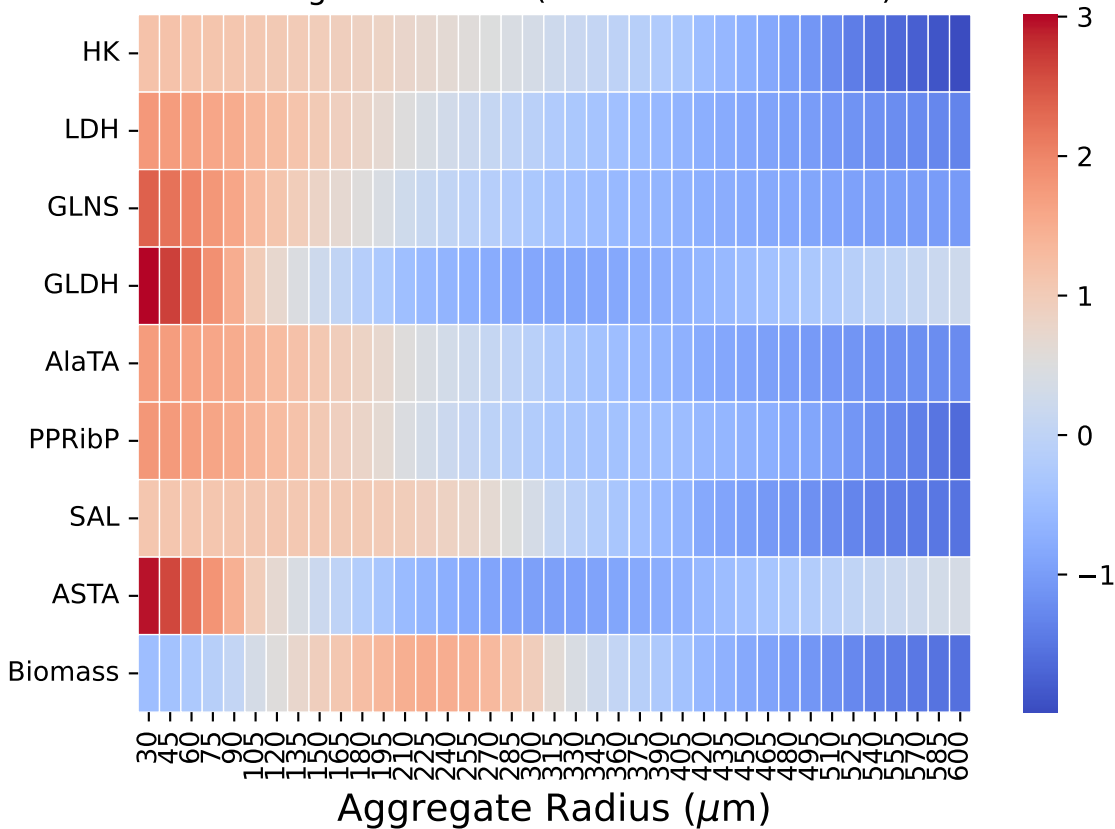

Supplement: Supplementary file 3 — Supplementary Software [file 42003_2023_5653_MOESM3_ESM.zip › MultiScaleModel-master/multi_scale_model/result/optimal_size/Biomass-reaction-ala-0.05.pdf]

### Averaged Flux Rates (after standardization)

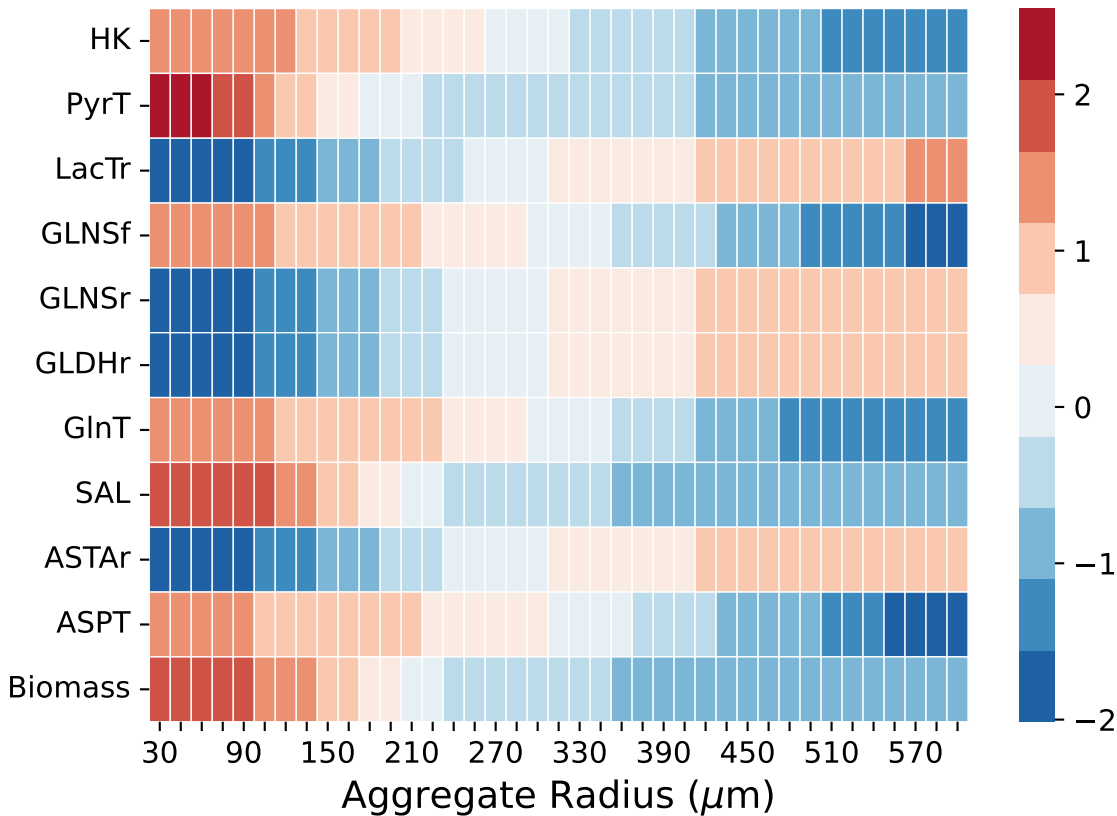

Supplement: Supplementary file 3 — Supplementary Software [file 42003_2023_5653_MOESM3_ESM.zip › MultiScaleModel-master/multi_scale_model/result/optimal_size/Biomass-reaction-ala-0.1.pdf]

Averaged Flux Rates (after standardization)

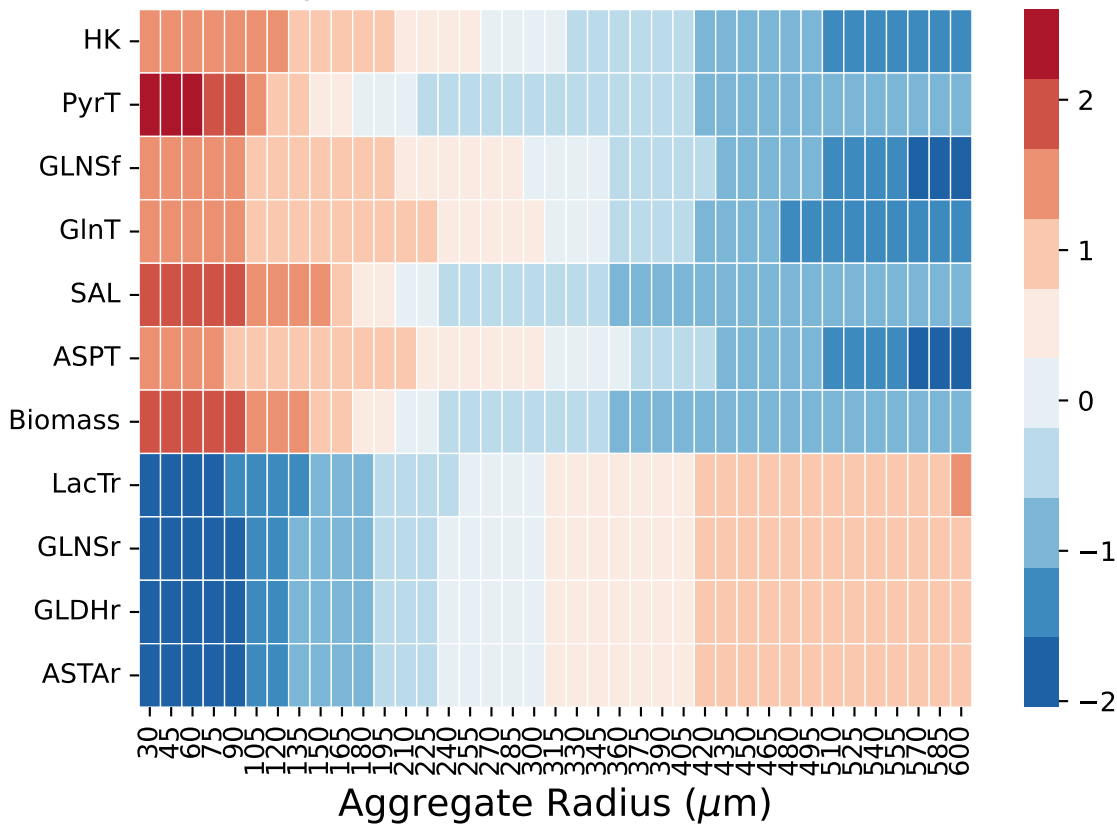

Supplement: Supplementary file 3 — Supplementary Software [file 42003_2023_5653_MOESM3_ESM.zip › MultiScaleModel-master/multi_scale_model/result/optimal_size/Biomass-reaction-ordered-ala-0.1.pdf]

Average Flux Rates (after standardization)

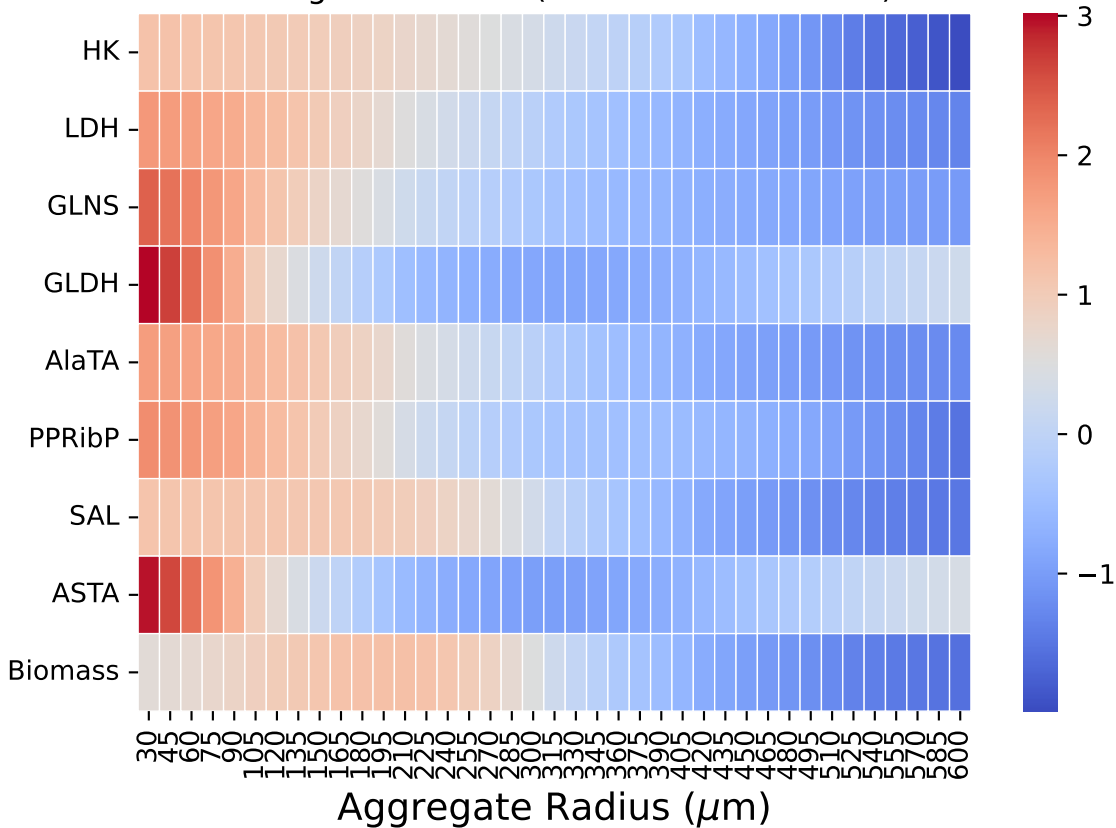

Supplement: Supplementary file 3 — Supplementary Software [file 42003_2023_5653_MOESM3_ESM.zip › MultiScaleModel-master/multi_scale_model/result/optimal_size/Biomass-reaction.pdf]

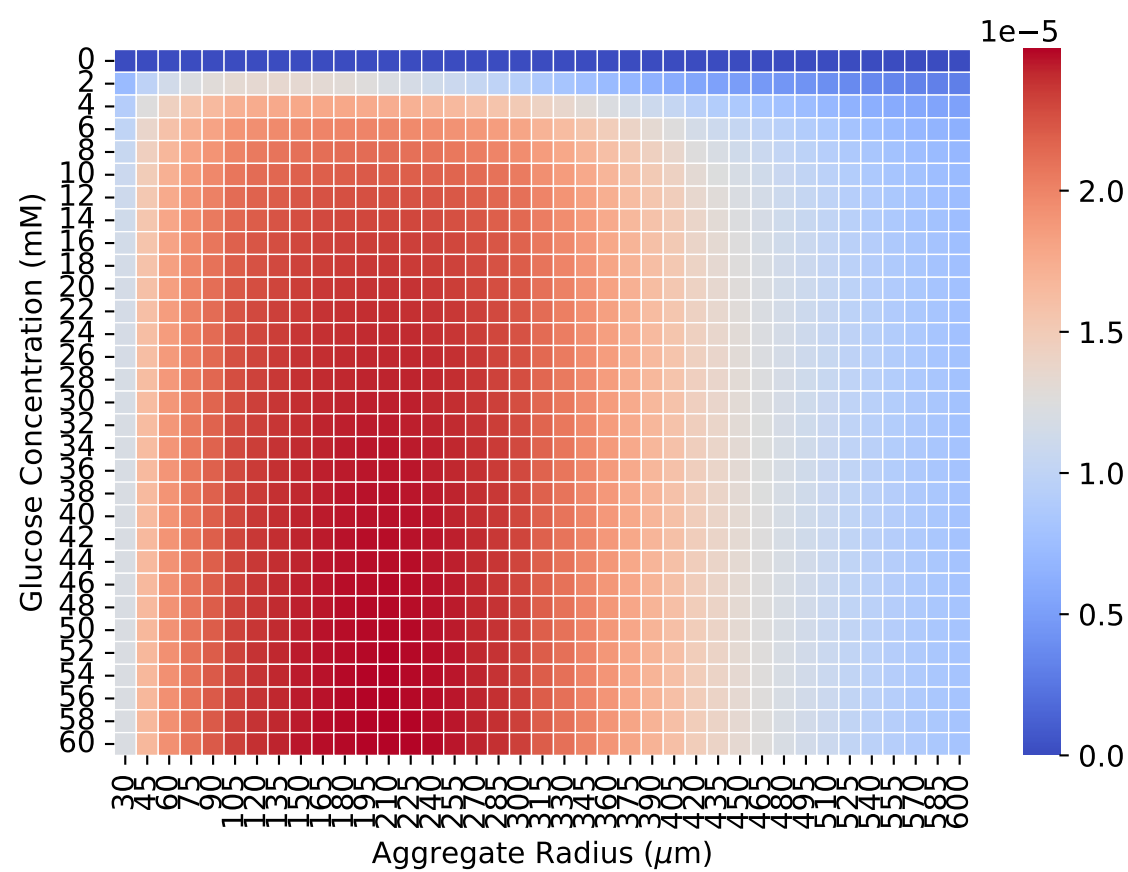

Supplement: Supplementary file 3 — Supplementary Software [file 42003_2023_5653_MOESM3_ESM.zip › MultiScaleModel-master/multi_scale_model/result/optimal_size/Glc-40-2.5.pdf]

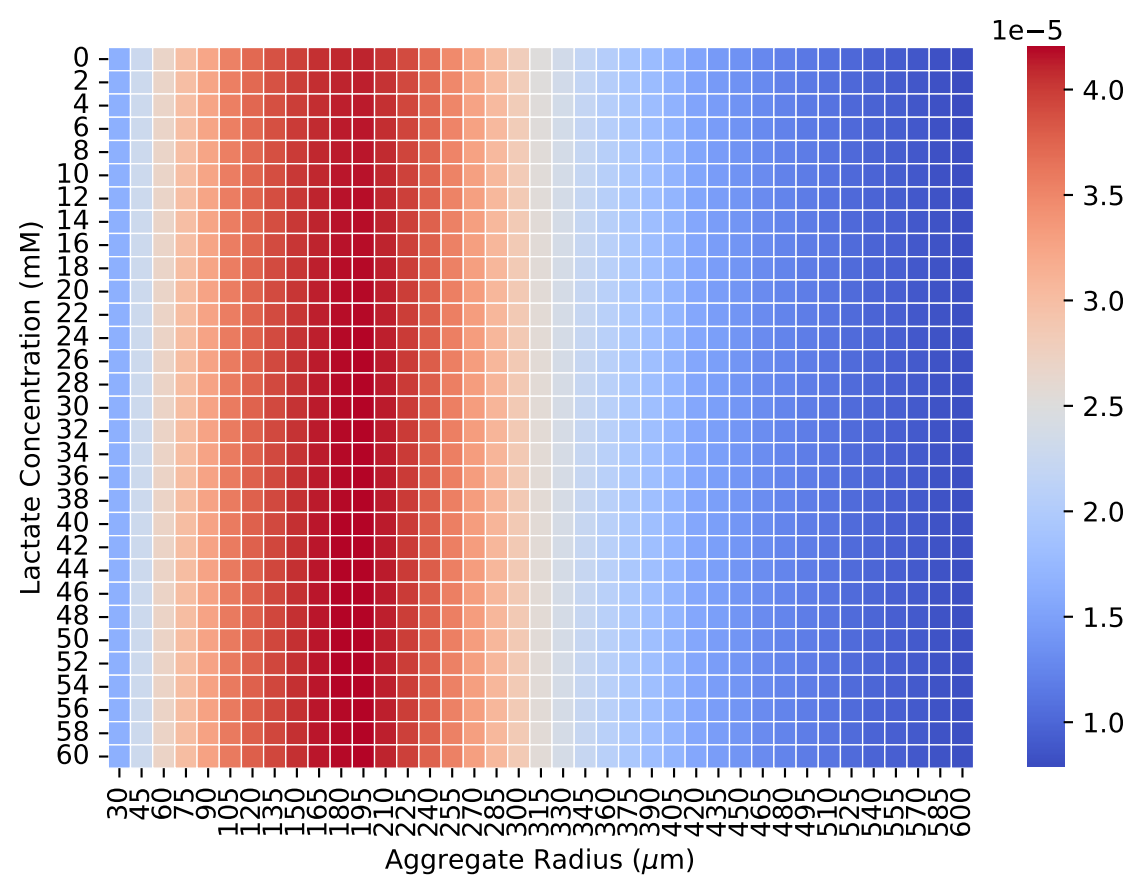

Supplement: Supplementary file 3 — Supplementary Software [file 42003_2023_5653_MOESM3_ESM.zip › MultiScaleModel-master/multi_scale_model/result/optimal_size/Lac-40-2.5.pdf]

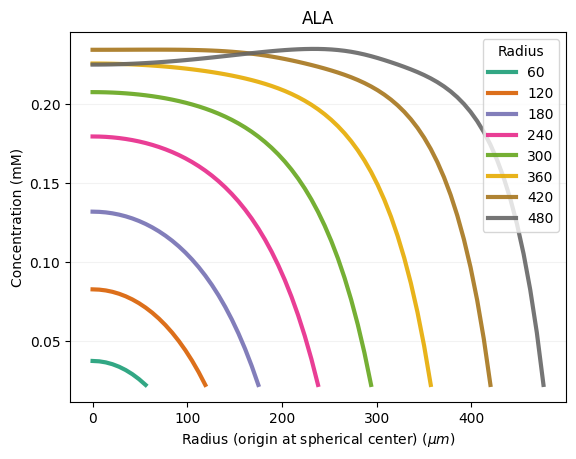

Supplement: Supplementary file 3 — Supplementary Software [file 42003_2023_5653_MOESM3_ESM.zip › MultiScaleModel-master/multi_scale_model/result/reaction-diffusion-2-500/ALA.png]

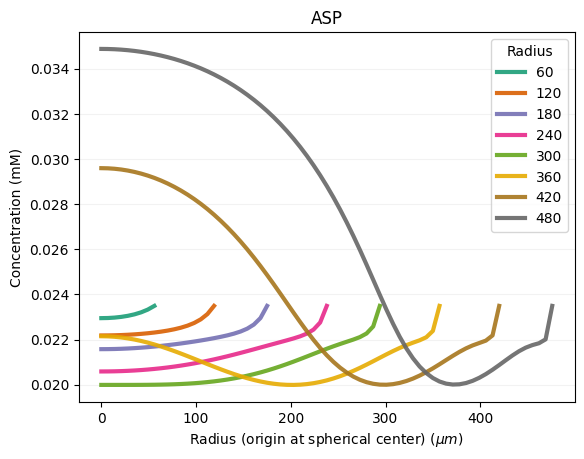

Supplement: Supplementary file 3 — Supplementary Software [file 42003_2023_5653_MOESM3_ESM.zip › MultiScaleModel-master/multi_scale_model/result/reaction-diffusion-2-500/ASP.png]

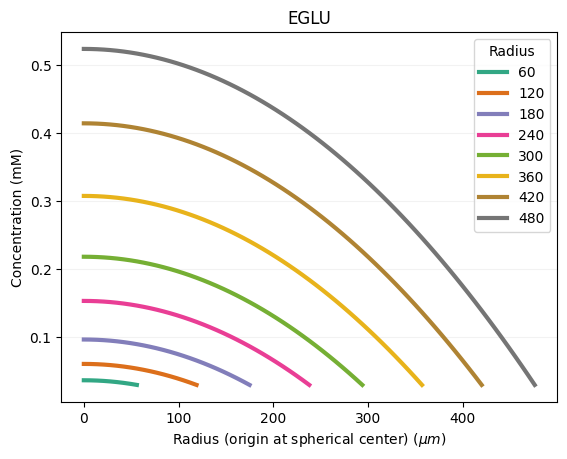

Supplement: Supplementary file 3 — Supplementary Software [file 42003_2023_5653_MOESM3_ESM.zip › MultiScaleModel-master/multi_scale_model/result/reaction-diffusion-2-500/EGLU.png]

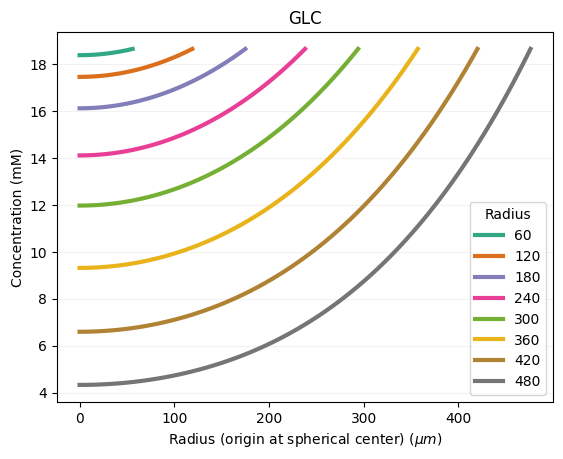

Supplement: Supplementary file 3 — Supplementary Software [file 42003_2023_5653_MOESM3_ESM.zip › MultiScaleModel-master/multi_scale_model/result/reaction-diffusion-2-500/GLC.png]

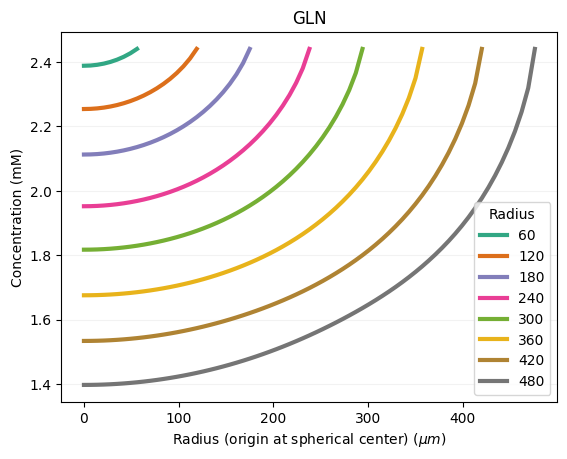

Supplement: Supplementary file 3 — Supplementary Software [file 42003_2023_5653_MOESM3_ESM.zip › MultiScaleModel-master/multi_scale_model/result/reaction-diffusion-2-500/GLN.png]

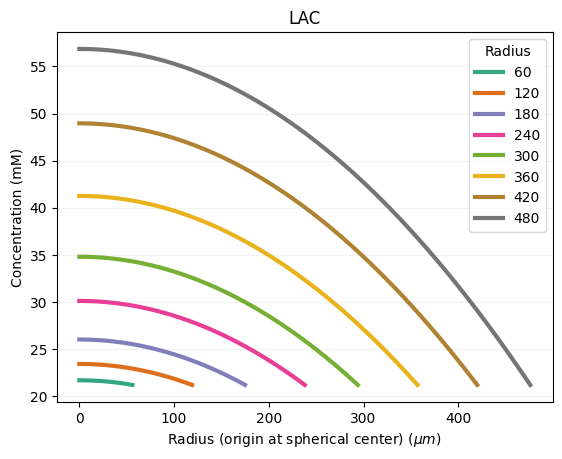

Supplement: Supplementary file 3 — Supplementary Software [file 42003_2023_5653_MOESM3_ESM.zip › MultiScaleModel-master/multi_scale_model/result/reaction-diffusion-2-500/LAC.png]

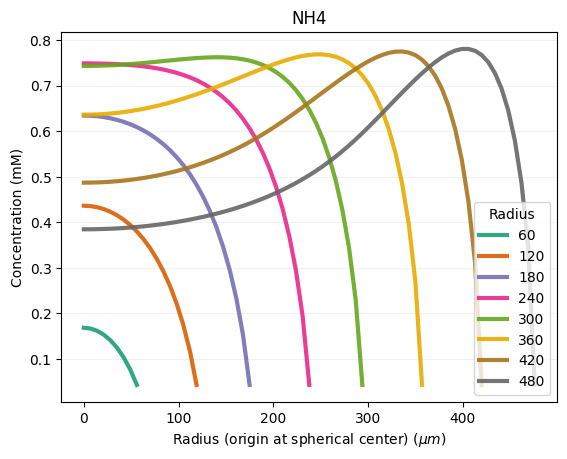

Supplement: Supplementary file 3 — Supplementary Software [file 42003_2023_5653_MOESM3_ESM.zip › MultiScaleModel-master/multi_scale_model/result/reaction-diffusion-2-500/NH4.png]

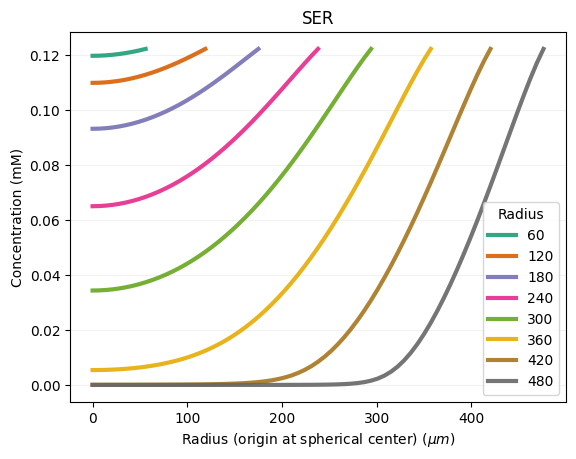

Supplement: Supplementary file 3 — Supplementary Software [file 42003_2023_5653_MOESM3_ESM.zip › MultiScaleModel-master/multi_scale_model/result/reaction-diffusion-2-500/SER.png]

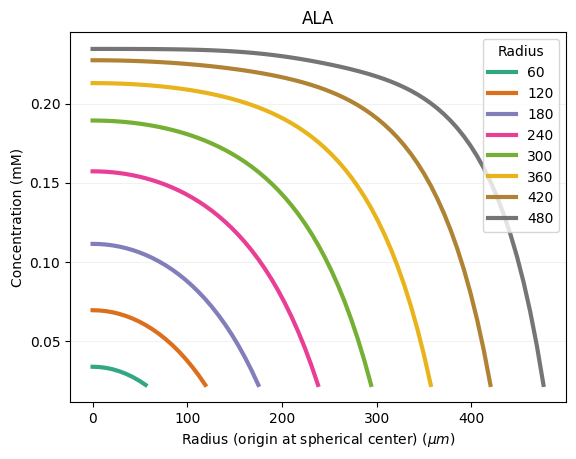

Supplement: Supplementary file 3 — Supplementary Software [file 42003_2023_5653_MOESM3_ESM.zip › MultiScaleModel-master/multi_scale_model/result/reaction-diffusion-3-1000/ALA.png]

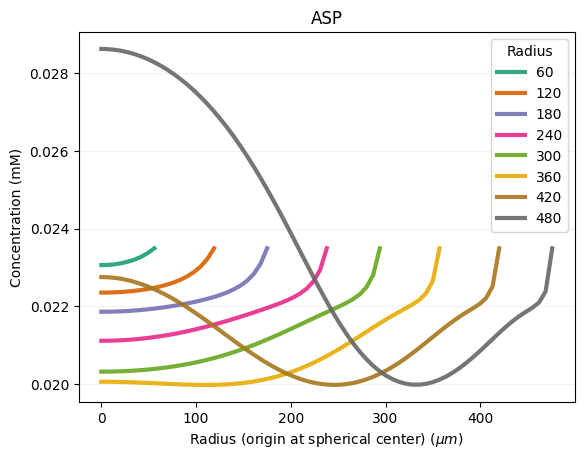

Supplement: Supplementary file 3 — Supplementary Software [file 42003_2023_5653_MOESM3_ESM.zip › MultiScaleModel-master/multi_scale_model/result/reaction-diffusion-3-1000/ASP.png]

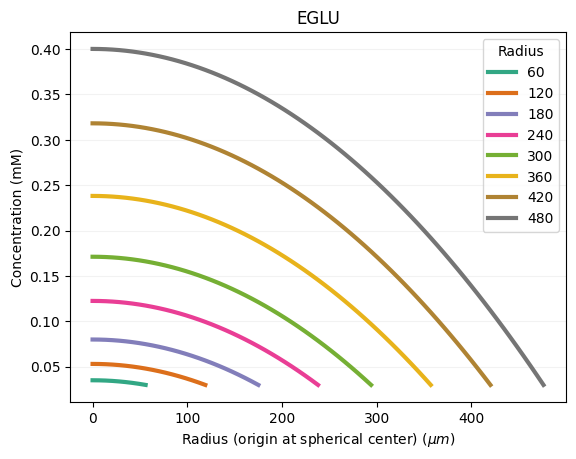

Supplement: Supplementary file 3 — Supplementary Software [file 42003_2023_5653_MOESM3_ESM.zip › MultiScaleModel-master/multi_scale_model/result/reaction-diffusion-3-1000/EGLU.png]

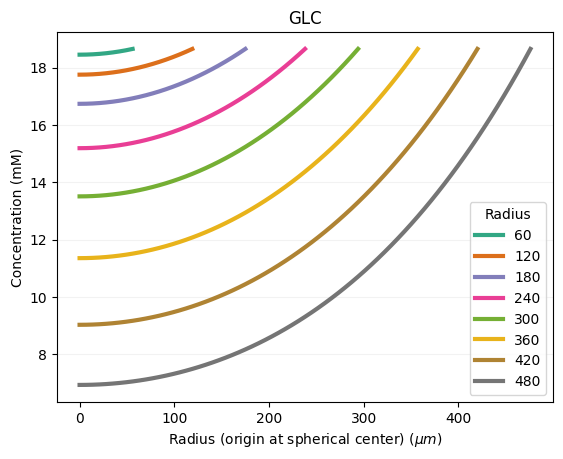

Supplement: Supplementary file 3 — Supplementary Software [file 42003_2023_5653_MOESM3_ESM.zip › MultiScaleModel-master/multi_scale_model/result/reaction-diffusion-3-1000/GLC.png]

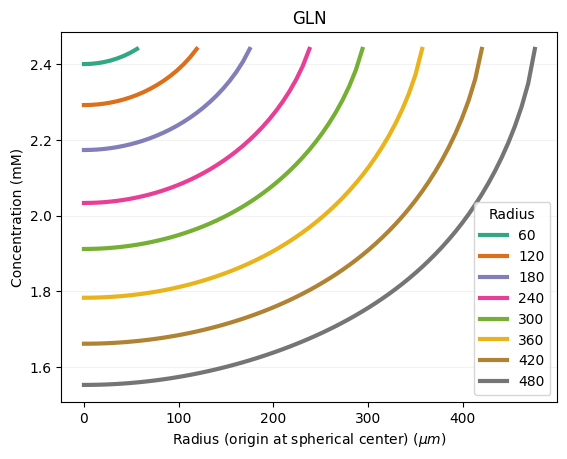

Supplement: Supplementary file 3 — Supplementary Software [file 42003_2023_5653_MOESM3_ESM.zip › MultiScaleModel-master/multi_scale_model/result/reaction-diffusion-3-1000/GLN.png]

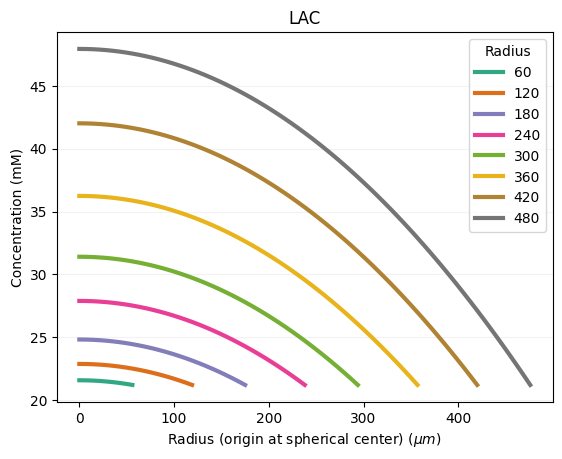

Supplement: Supplementary file 3 — Supplementary Software [file 42003_2023_5653_MOESM3_ESM.zip › MultiScaleModel-master/multi_scale_model/result/reaction-diffusion-3-1000/LAC.png]

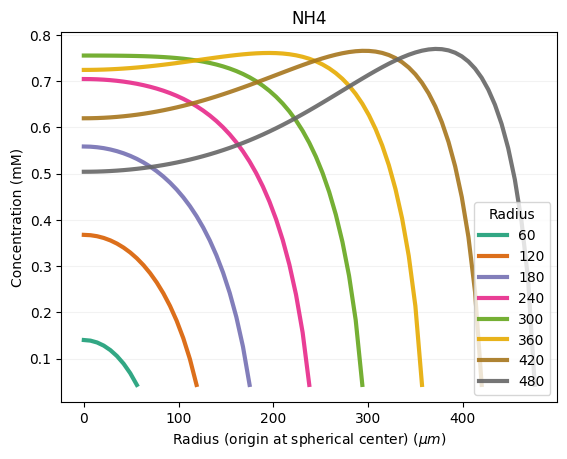

Supplement: Supplementary file 3 — Supplementary Software [file 42003_2023_5653_MOESM3_ESM.zip › MultiScaleModel-master/multi_scale_model/result/reaction-diffusion-3-1000/NH4.png]

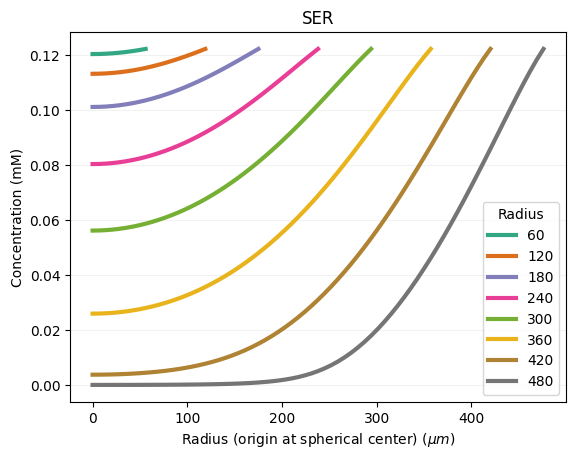

Supplement: Supplementary file 3 — Supplementary Software [file 42003_2023_5653_MOESM3_ESM.zip › MultiScaleModel-master/multi_scale_model/result/reaction-diffusion-3-1000/SER.png]

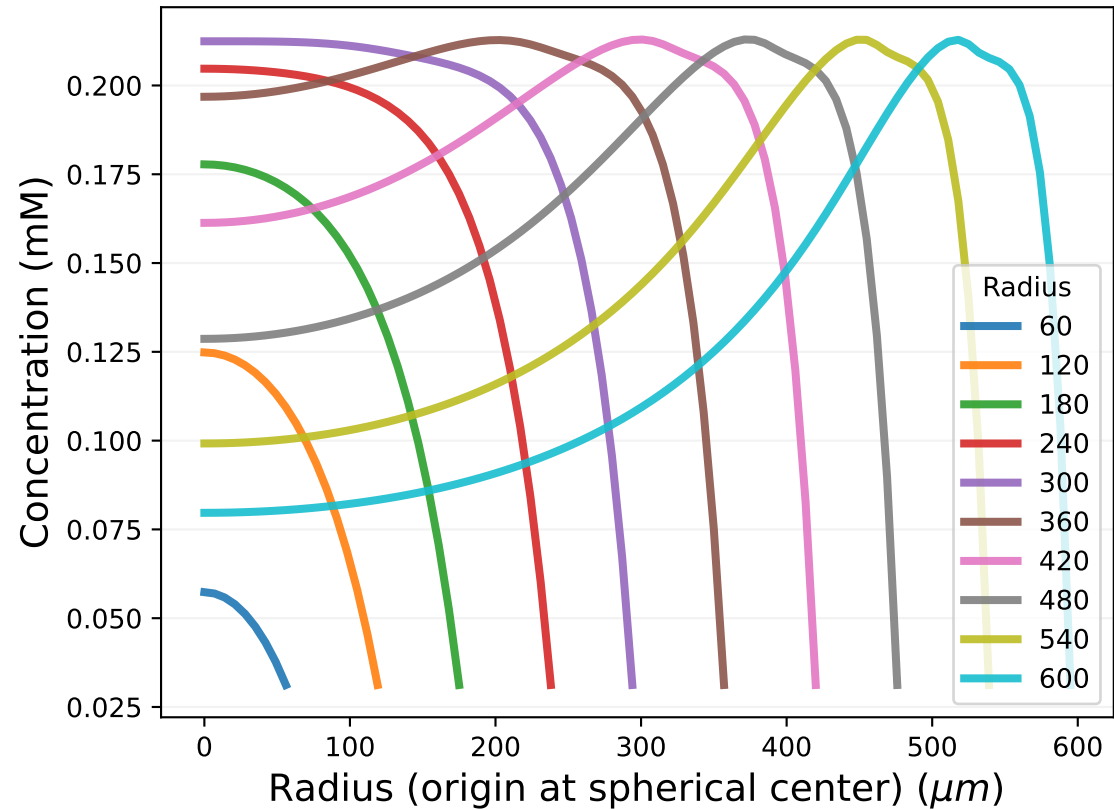

Supplement: Supplementary file 3 — Supplementary Software [file 42003_2023_5653_MOESM3_ESM.zip › MultiScaleModel-master/multi_scale_model/result/reaction-diffusion/ALA.pdf]

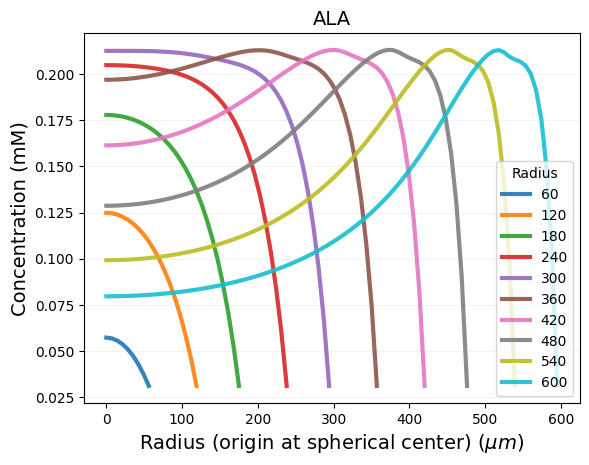

Supplement: Supplementary file 3 — Supplementary Software [file 42003_2023_5653_MOESM3_ESM.zip › MultiScaleModel-master/multi_scale_model/result/reaction-diffusion/ALA.png]

# ASP

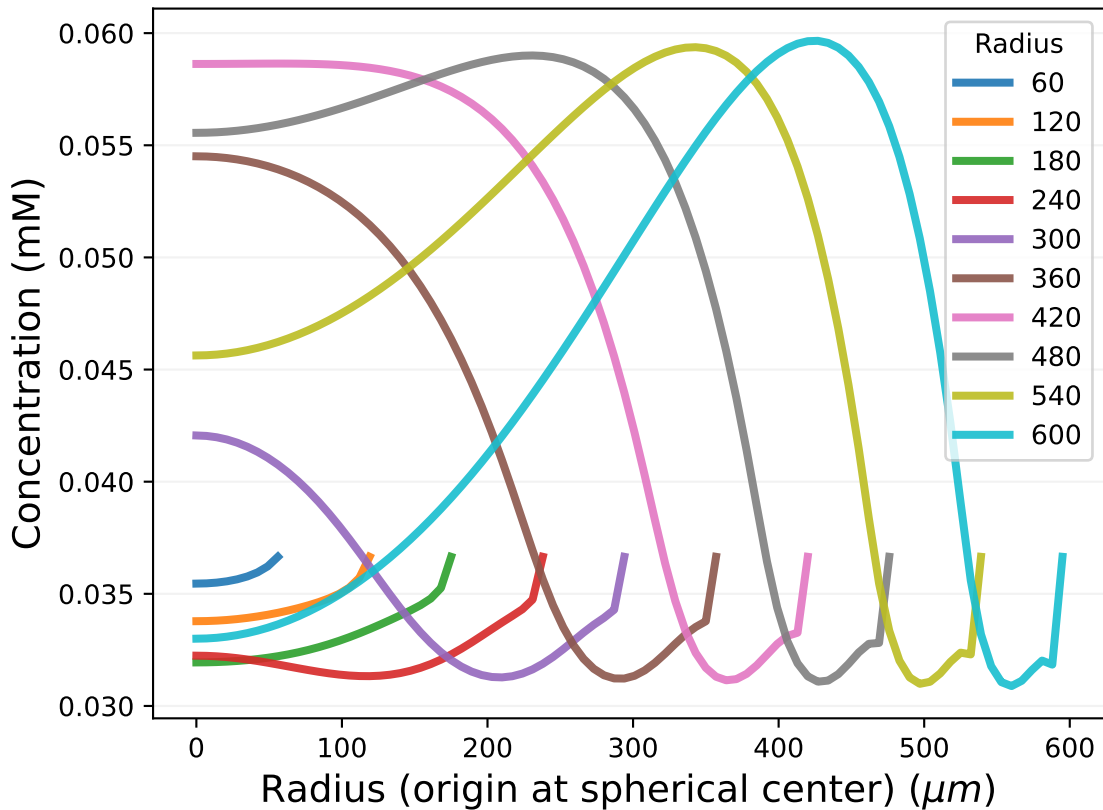

Supplement: Supplementary file 3 — Supplementary Software [file 42003_2023_5653_MOESM3_ESM.zip › MultiScaleModel-master/multi_scale_model/result/reaction-diffusion/ASP]

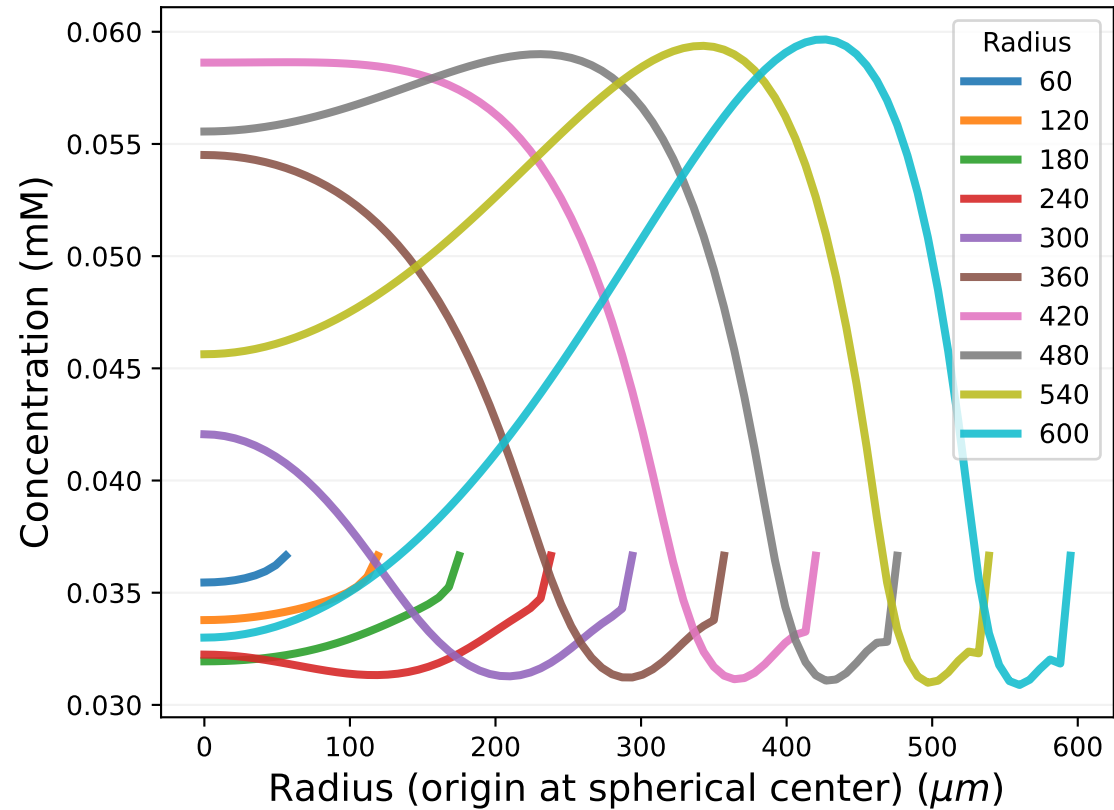

Supplement: Supplementary file 3 — Supplementary Software [file 42003_2023_5653_MOESM3_ESM.zip › MultiScaleModel-master/multi_scale_model/result/reaction-diffusion/ASP.pdf]

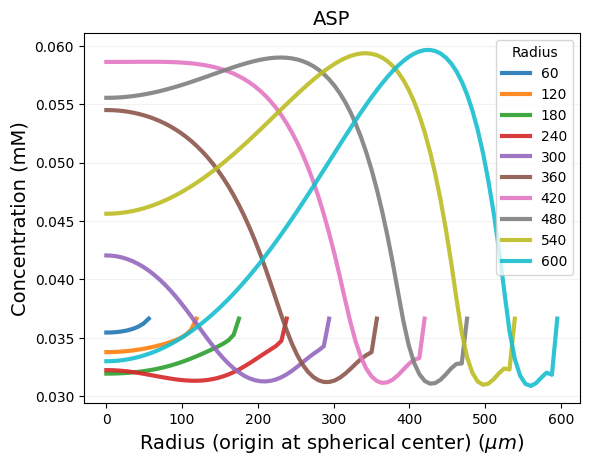

Supplement: Supplementary file 3 — Supplementary Software [file 42003_2023_5653_MOESM3_ESM.zip › MultiScaleModel-master/multi_scale_model/result/reaction-diffusion/ASP.png]

# EGLU

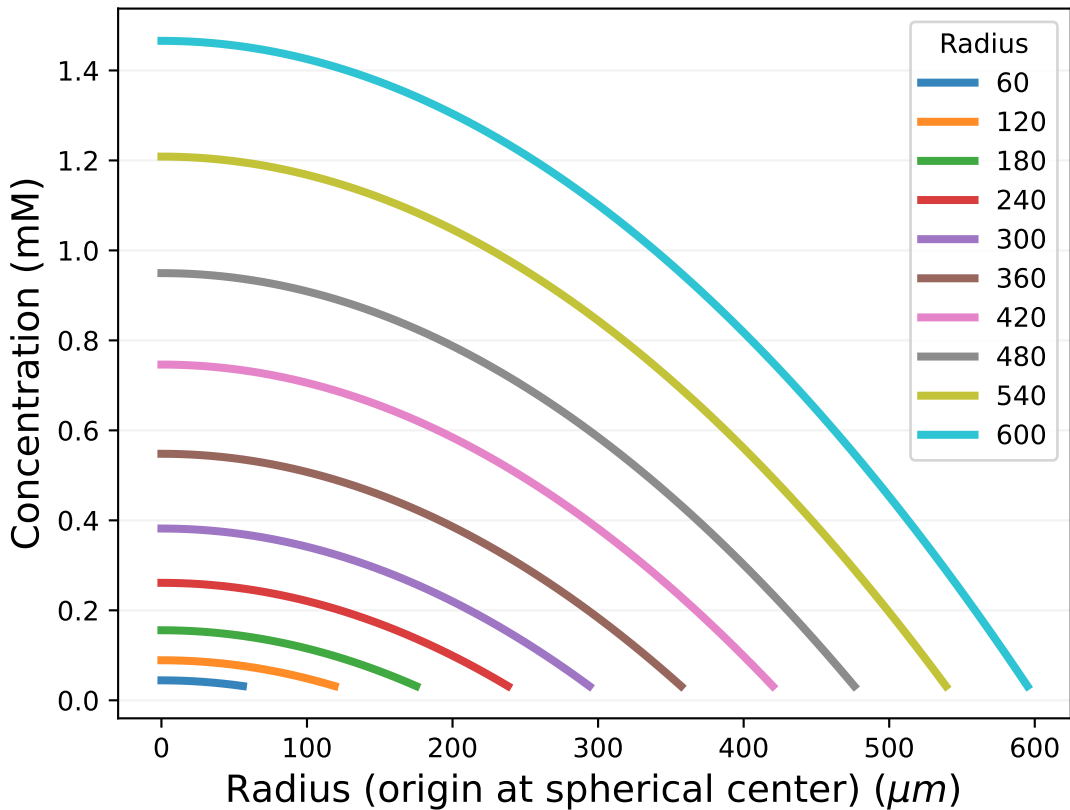

Supplement: Supplementary file 3 — Supplementary Software [file 42003_2023_5653_MOESM3_ESM.zip › MultiScaleModel-master/multi_scale_model/result/reaction-diffusion/EGLU]

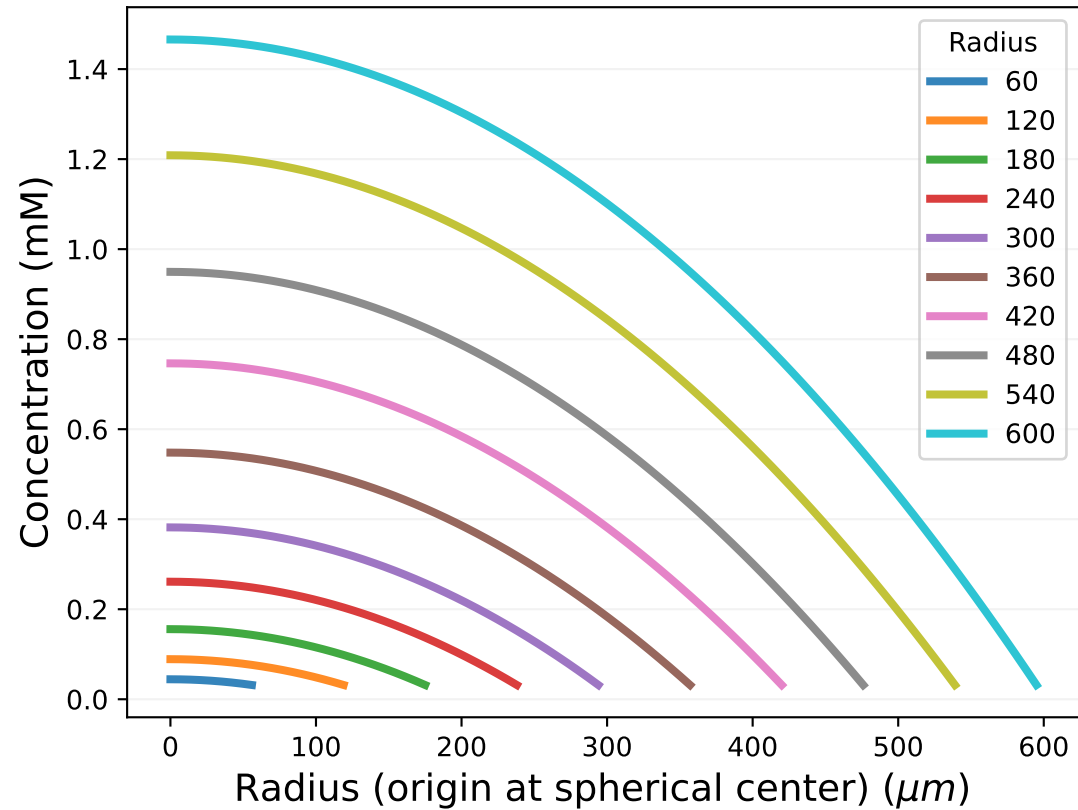

Supplement: Supplementary file 3 — Supplementary Software [file 42003_2023_5653_MOESM3_ESM.zip › MultiScaleModel-master/multi_scale_model/result/reaction-diffusion/EGLU.pdf]

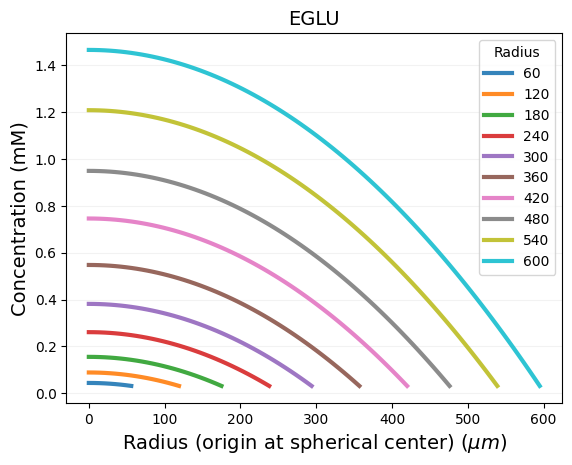

Supplement: Supplementary file 3 — Supplementary Software [file 42003_2023_5653_MOESM3_ESM.zip › MultiScaleModel-master/multi_scale_model/result/reaction-diffusion/EGLU.png]

## GLC

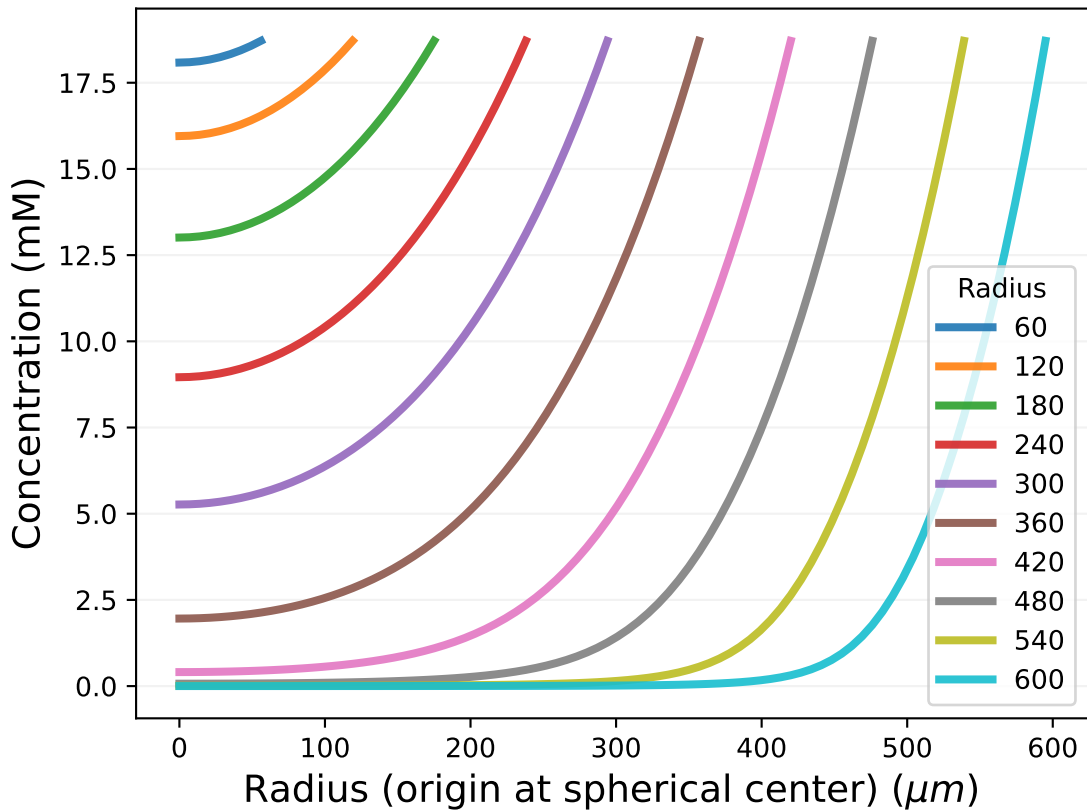

Supplement: Supplementary file 3 — Supplementary Software [file 42003_2023_5653_MOESM3_ESM.zip › MultiScaleModel-master/multi_scale_model/result/reaction-diffusion/GLC]

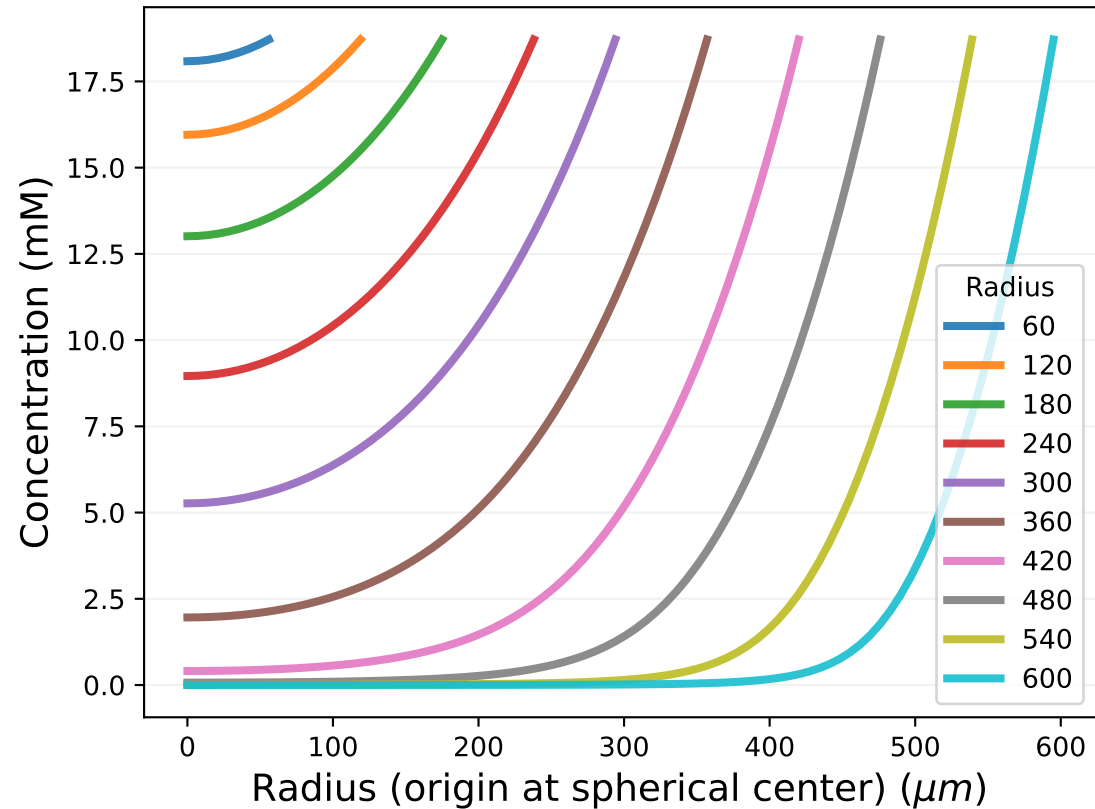

Supplement: Supplementary file 3 — Supplementary Software [file 42003_2023_5653_MOESM3_ESM.zip › MultiScaleModel-master/multi_scale_model/result/reaction-diffusion/GLC.pdf]

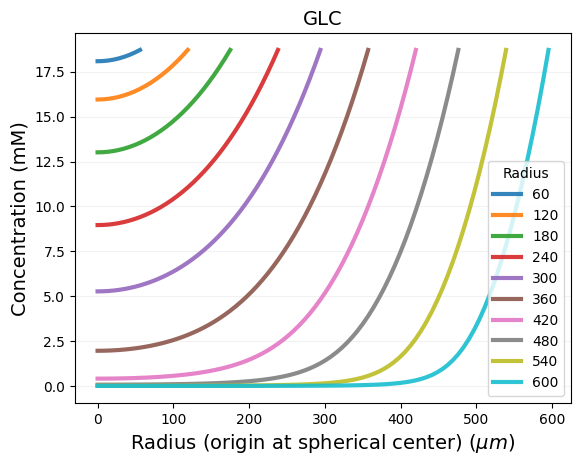

Supplement: Supplementary file 3 — Supplementary Software [file 42003_2023_5653_MOESM3_ESM.zip › MultiScaleModel-master/multi_scale_model/result/reaction-diffusion/GLC.png]

# GLN

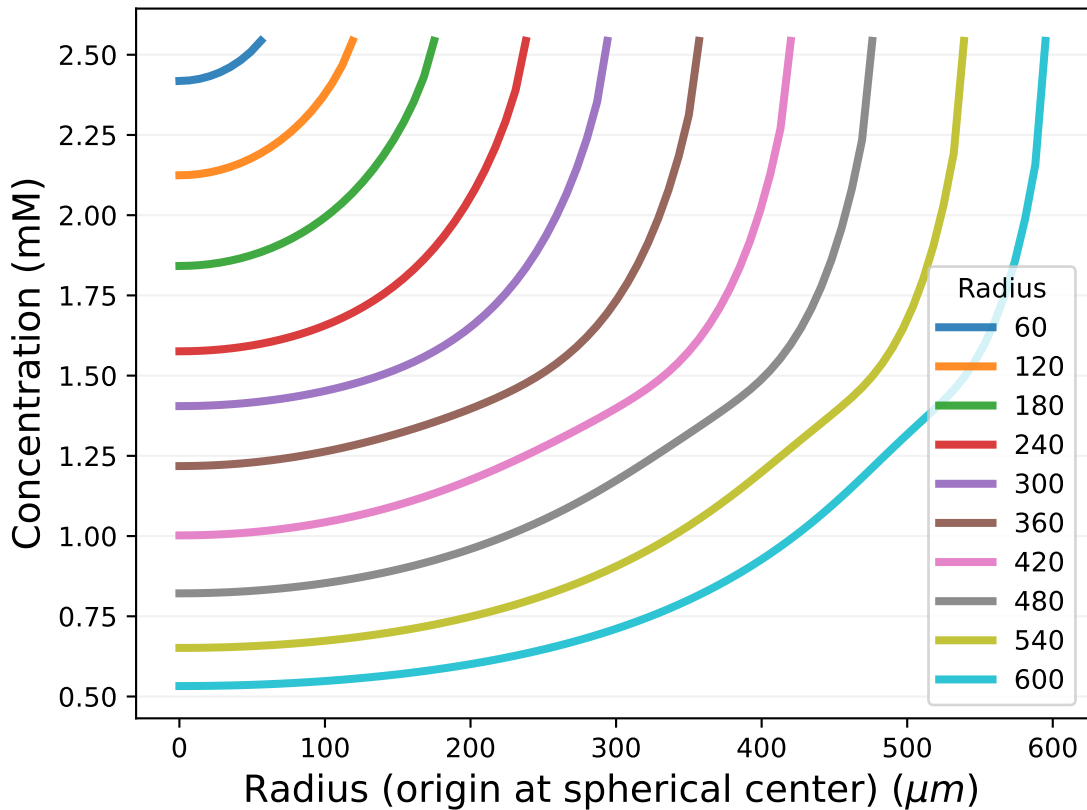

Supplement: Supplementary file 3 — Supplementary Software [file 42003_2023_5653_MOESM3_ESM.zip › MultiScaleModel-master/multi_scale_model/result/reaction-diffusion/GLN]

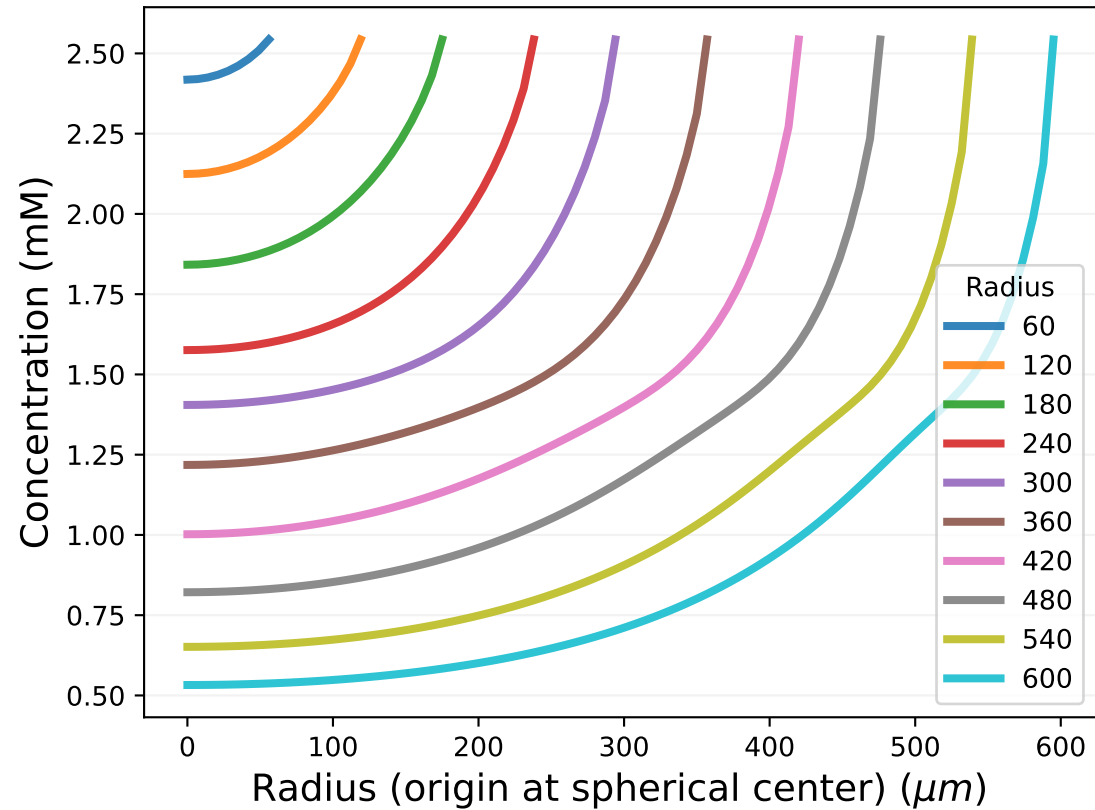

Supplement: Supplementary file 3 — Supplementary Software [file 42003_2023_5653_MOESM3_ESM.zip › MultiScaleModel-master/multi_scale_model/result/reaction-diffusion/GLN.pdf]

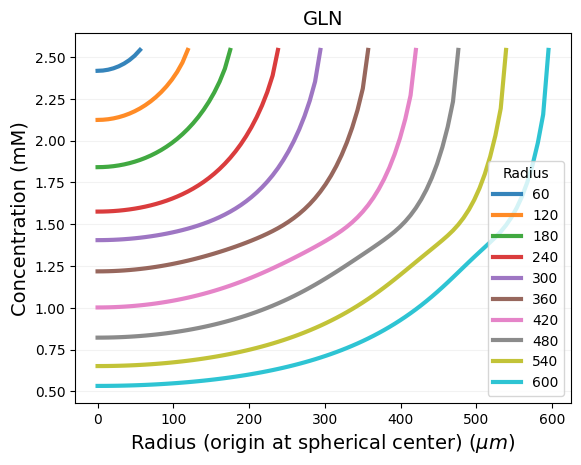

Supplement: Supplementary file 3 — Supplementary Software [file 42003_2023_5653_MOESM3_ESM.zip › MultiScaleModel-master/multi_scale_model/result/reaction-diffusion/GLN.png]

# GLU

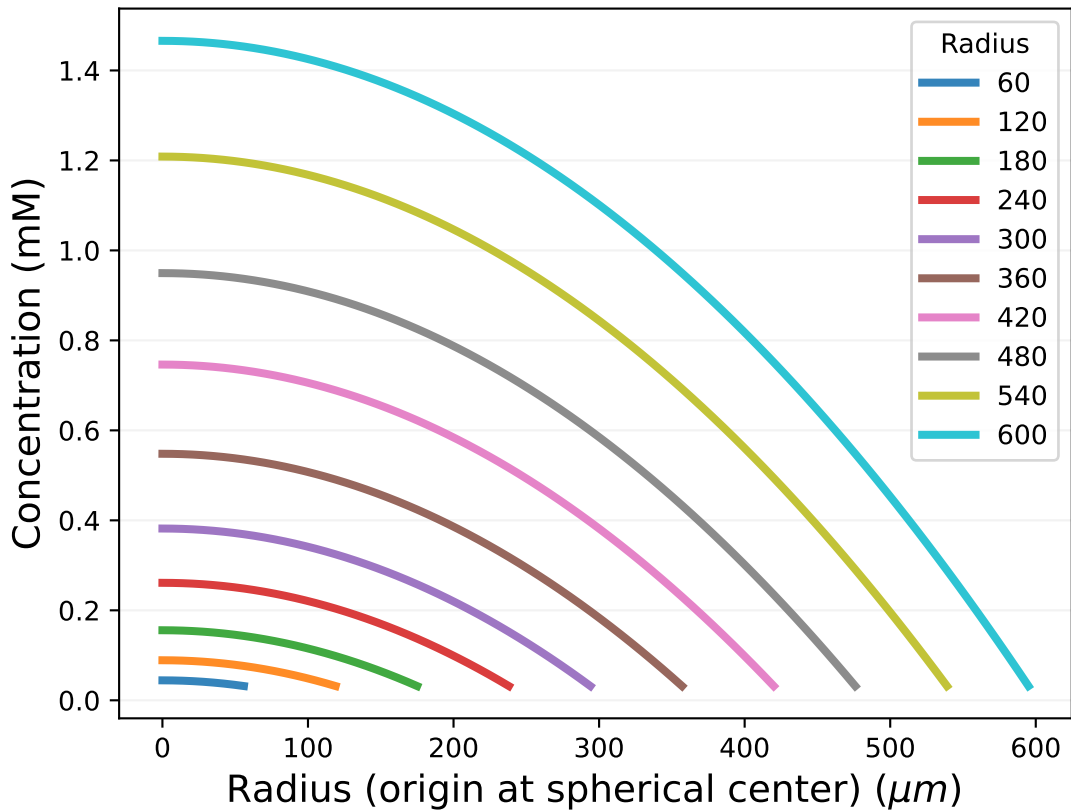

Supplement: Supplementary file 3 — Supplementary Software [file 42003_2023_5653_MOESM3_ESM.zip › MultiScaleModel-master/multi_scale_model/result/reaction-diffusion/GLU.pdf]

# LAC

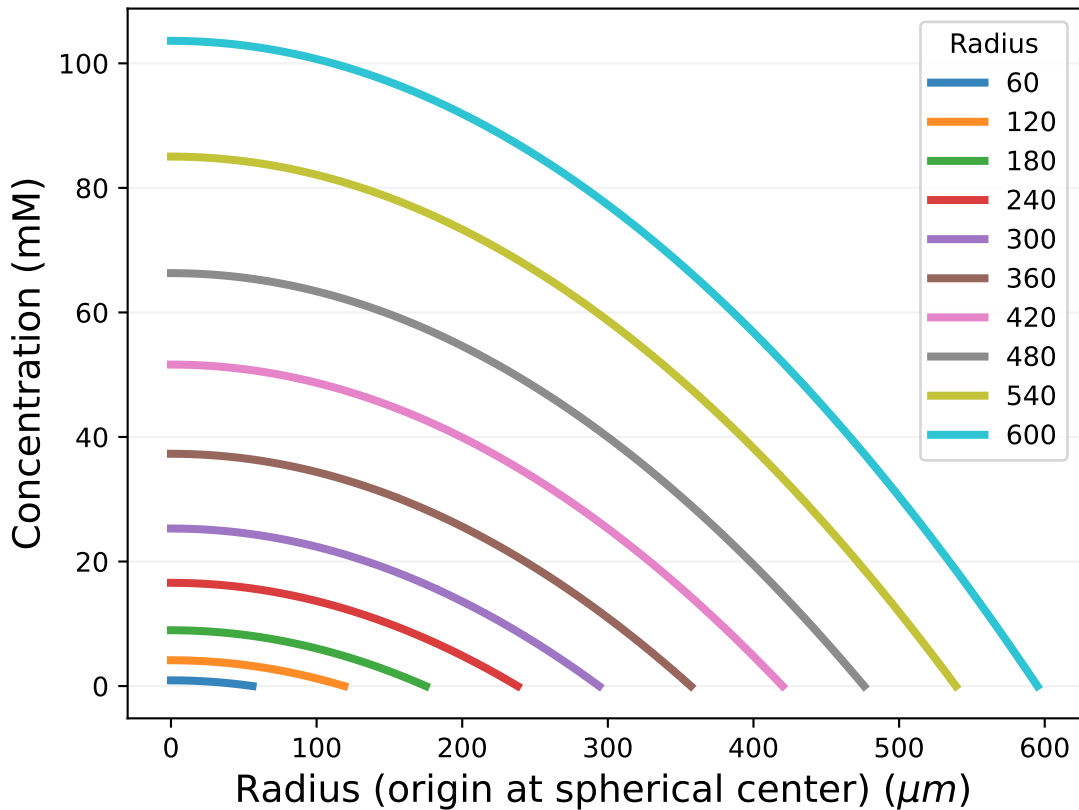

Supplement: Supplementary file 3 — Supplementary Software [file 42003_2023_5653_MOESM3_ESM.zip › MultiScaleModel-master/multi_scale_model/result/reaction-diffusion/LAC]

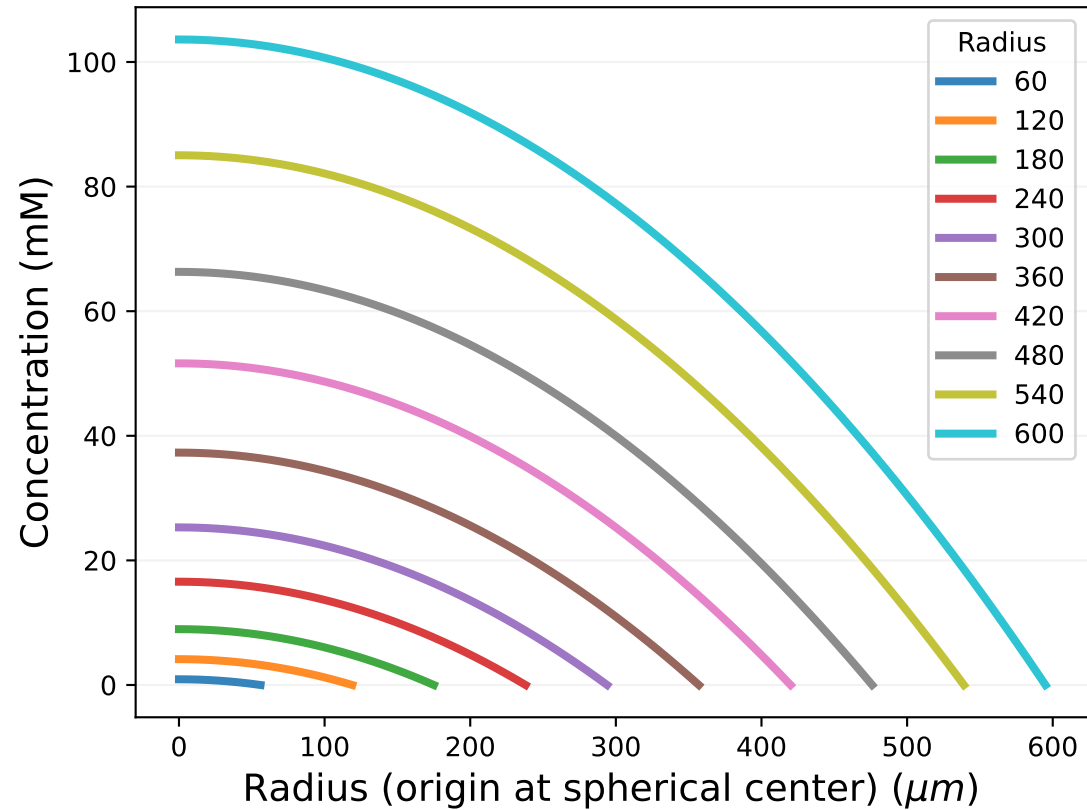

Supplement: Supplementary file 3 — Supplementary Software [file 42003_2023_5653_MOESM3_ESM.zip › MultiScaleModel-master/multi_scale_model/result/reaction-diffusion/LAC.pdf]

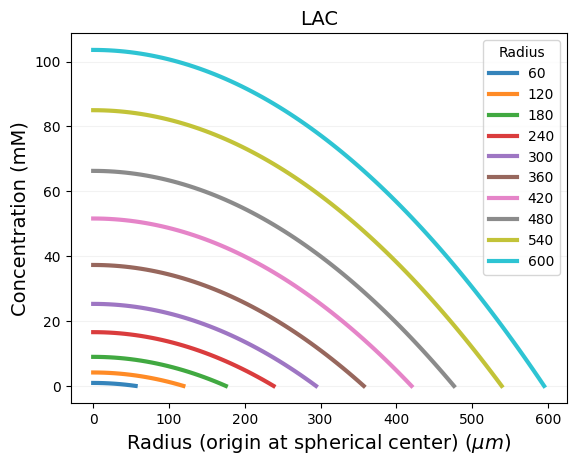

Supplement: Supplementary file 3 — Supplementary Software [file 42003_2023_5653_MOESM3_ESM.zip › MultiScaleModel-master/multi_scale_model/result/reaction-diffusion/LAC.png]

NH<sub>4</sub>

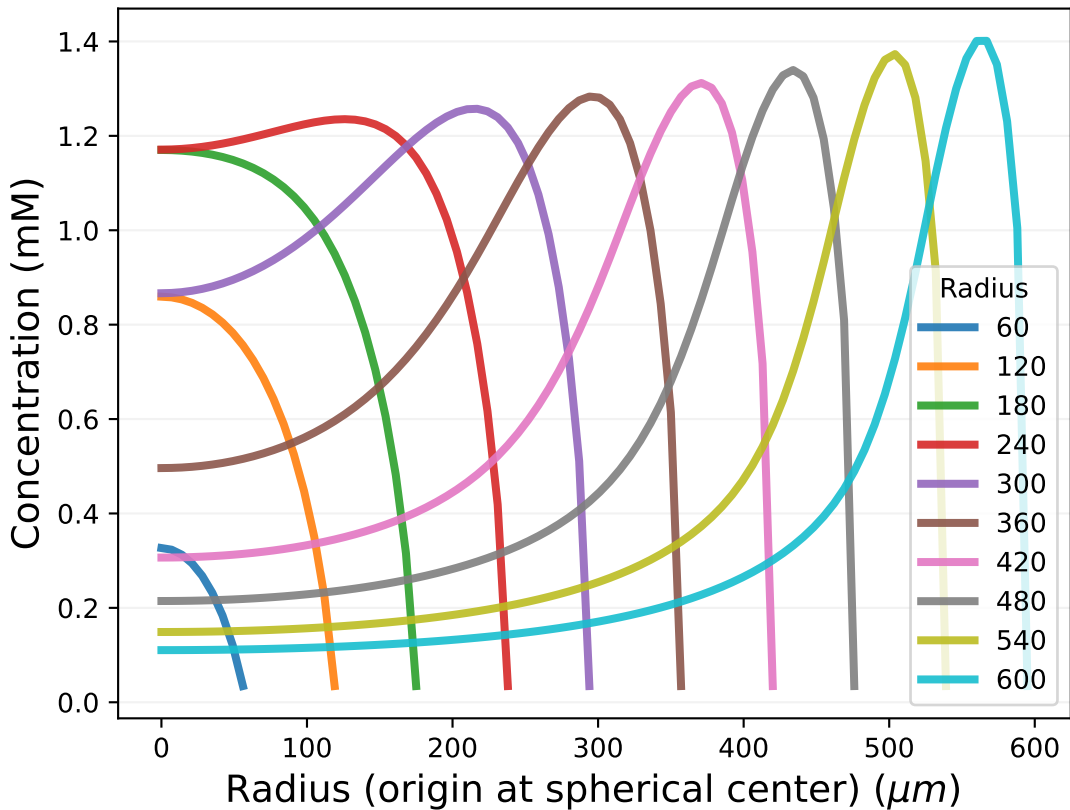

Supplement: Supplementary file 3 — Supplementary Software [file 42003_2023_5653_MOESM3_ESM.zip › MultiScaleModel-master/multi_scale_model/result/reaction-diffusion/NH4]

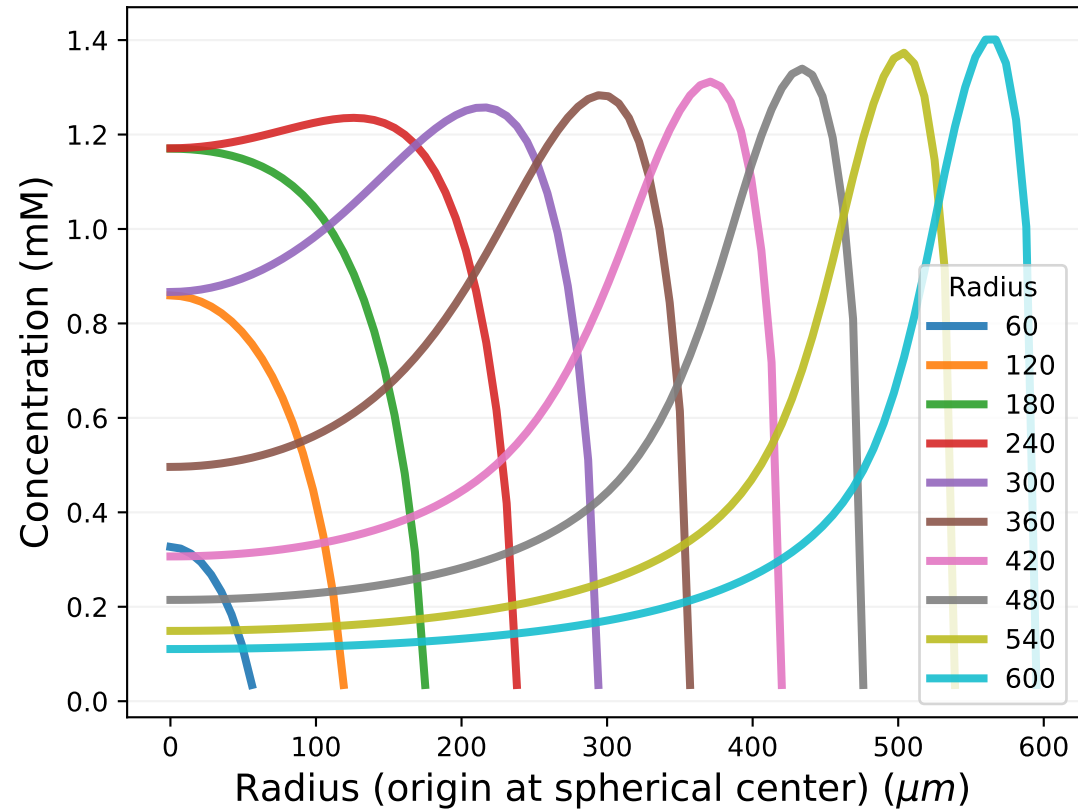

Supplement: Supplementary file 3 — Supplementary Software [file 42003_2023_5653_MOESM3_ESM.zip › MultiScaleModel-master/multi_scale_model/result/reaction-diffusion/NH4.pdf]

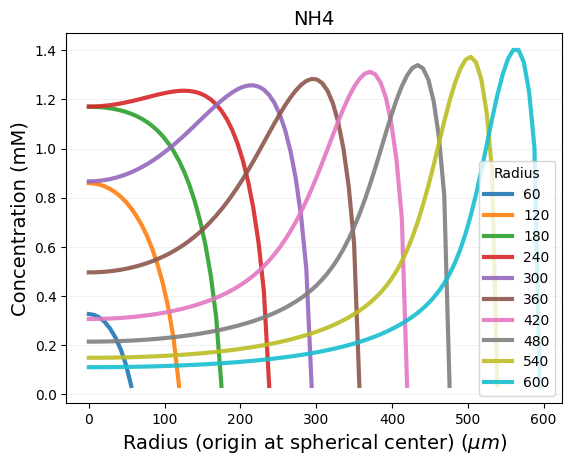

Supplement: Supplementary file 3 — Supplementary Software [file 42003_2023_5653_MOESM3_ESM.zip › MultiScaleModel-master/multi_scale_model/result/reaction-diffusion/NH4.png]

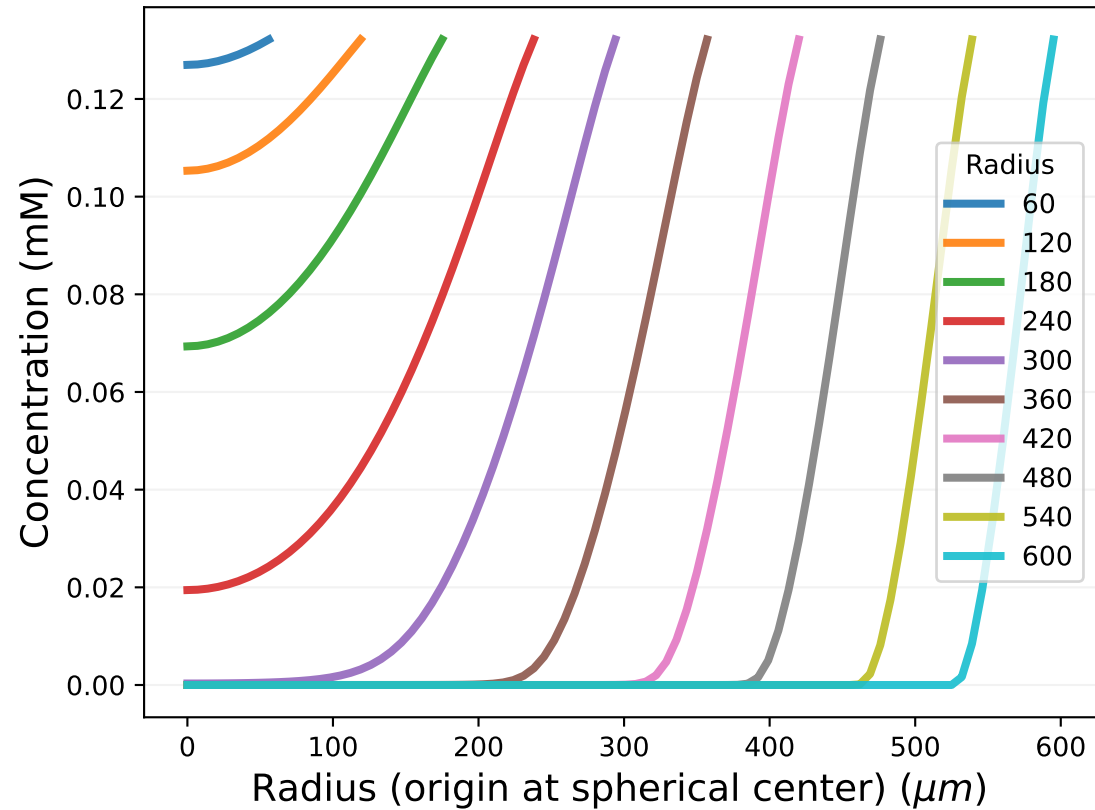

Supplement: Supplementary file 3 — Supplementary Software [file 42003_2023_5653_MOESM3_ESM.zip › MultiScaleModel-master/multi_scale_model/result/reaction-diffusion/SER.pdf]

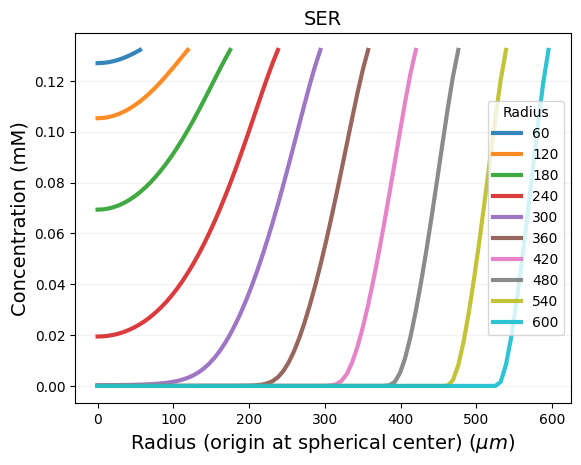

Supplement: Supplementary file 3 — Supplementary Software [file 42003_2023_5653_MOESM3_ESM.zip › MultiScaleModel-master/multi_scale_model/result/reaction-diffusion/SER.png]

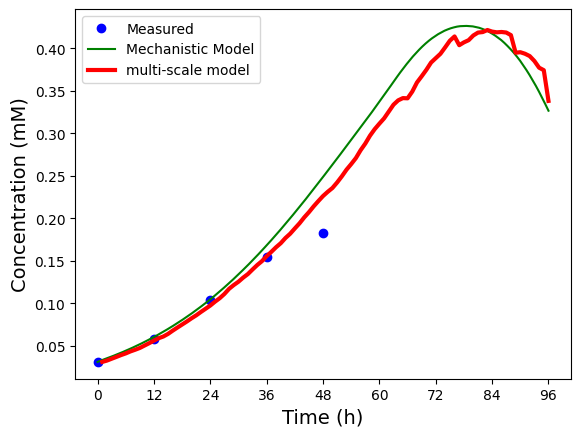

Supplement: Supplementary file 3 — Supplementary Software [file 42003_2023_5653_MOESM3_ESM.zip › MultiScaleModel-master/multi_scale_model/result/simulation-agg/HGLL-28.png]

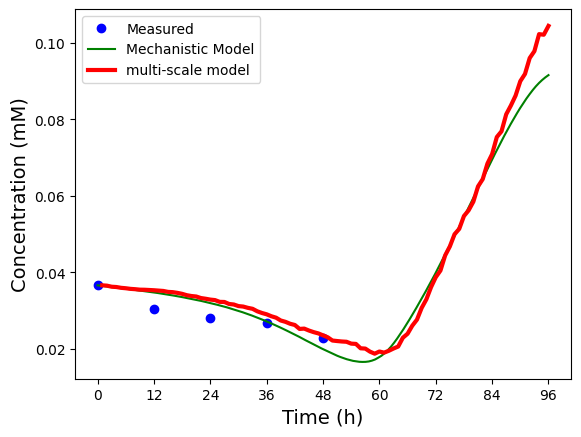

Supplement: Supplementary file 3 — Supplementary Software [file 42003_2023_5653_MOESM3_ESM.zip › MultiScaleModel-master/multi_scale_model/result/simulation-agg/HGLL-29.png]

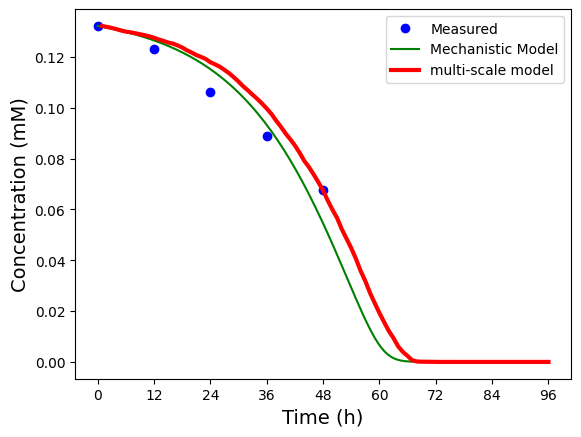

Supplement: Supplementary file 3 — Supplementary Software [file 42003_2023_5653_MOESM3_ESM.zip › MultiScaleModel-master/multi_scale_model/result/simulation-agg/HGLL-36.png]

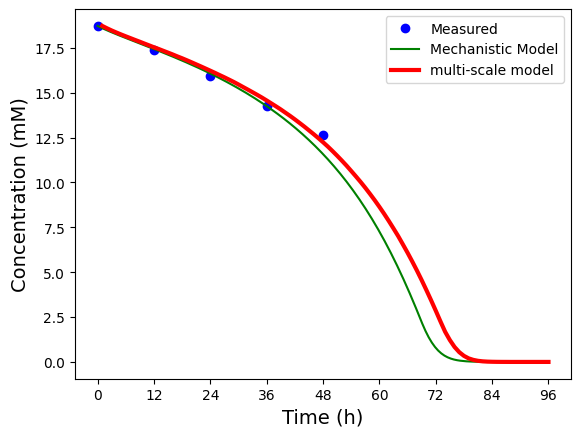

Supplement: Supplementary file 3 — Supplementary Software [file 42003_2023_5653_MOESM3_ESM.zip › MultiScaleModel-master/multi_scale_model/result/simulation-agg/HGLL-39.png]

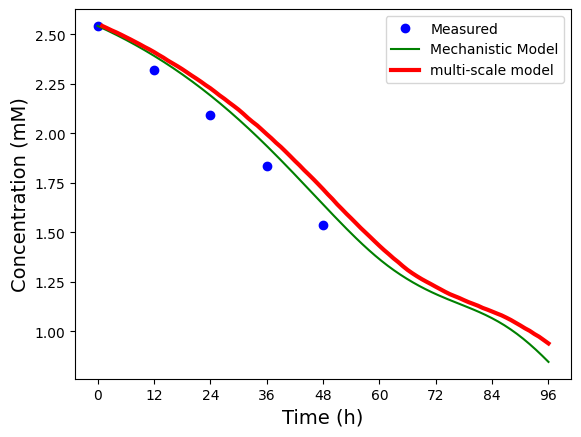

Supplement: Supplementary file 3 — Supplementary Software [file 42003_2023_5653_MOESM3_ESM.zip › MultiScaleModel-master/multi_scale_model/result/simulation-agg/HGLL-40.png]

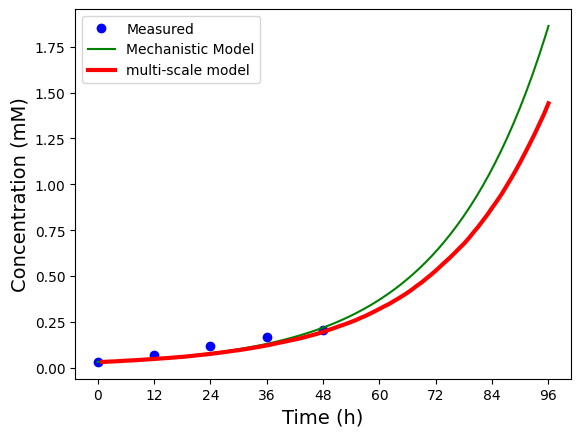

Supplement: Supplementary file 3 — Supplementary Software [file 42003_2023_5653_MOESM3_ESM.zip › MultiScaleModel-master/multi_scale_model/result/simulation-agg/HGLL-41.png]

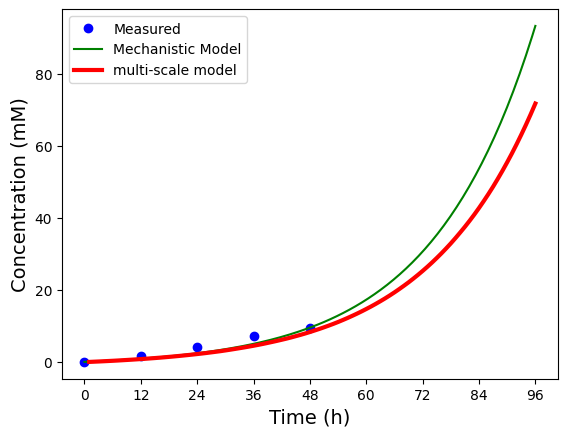

Supplement: Supplementary file 3 — Supplementary Software [file 42003_2023_5653_MOESM3_ESM.zip › MultiScaleModel-master/multi_scale_model/result/simulation-agg/HGLL-42.png]

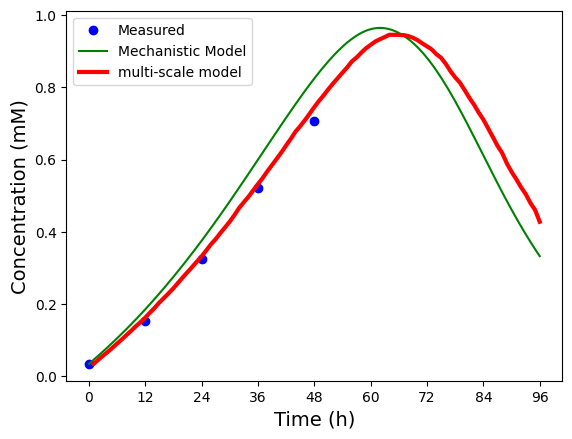

Supplement: Supplementary file 3 — Supplementary Software [file 42003_2023_5653_MOESM3_ESM.zip › MultiScaleModel-master/multi_scale_model/result/simulation-agg/HGLL-43.png]

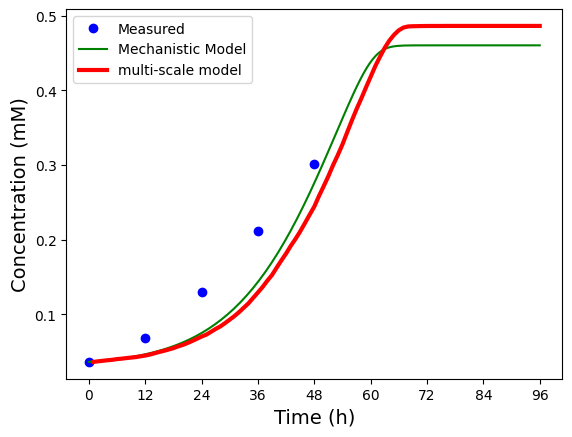

Supplement: Supplementary file 3 — Supplementary Software [file 42003_2023_5653_MOESM3_ESM.zip › MultiScaleModel-master/multi_scale_model/result/simulation-agg/HGLL-44.png]

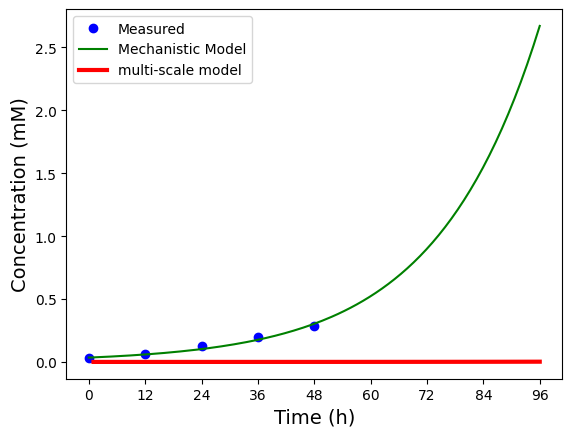

Supplement: Supplementary file 3 — Supplementary Software [file 42003_2023_5653_MOESM3_ESM.zip › MultiScaleModel-master/multi_scale_model/result/simulation-agg/HGLL-45.png]

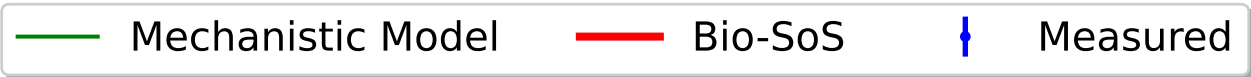

Concentration (mM)

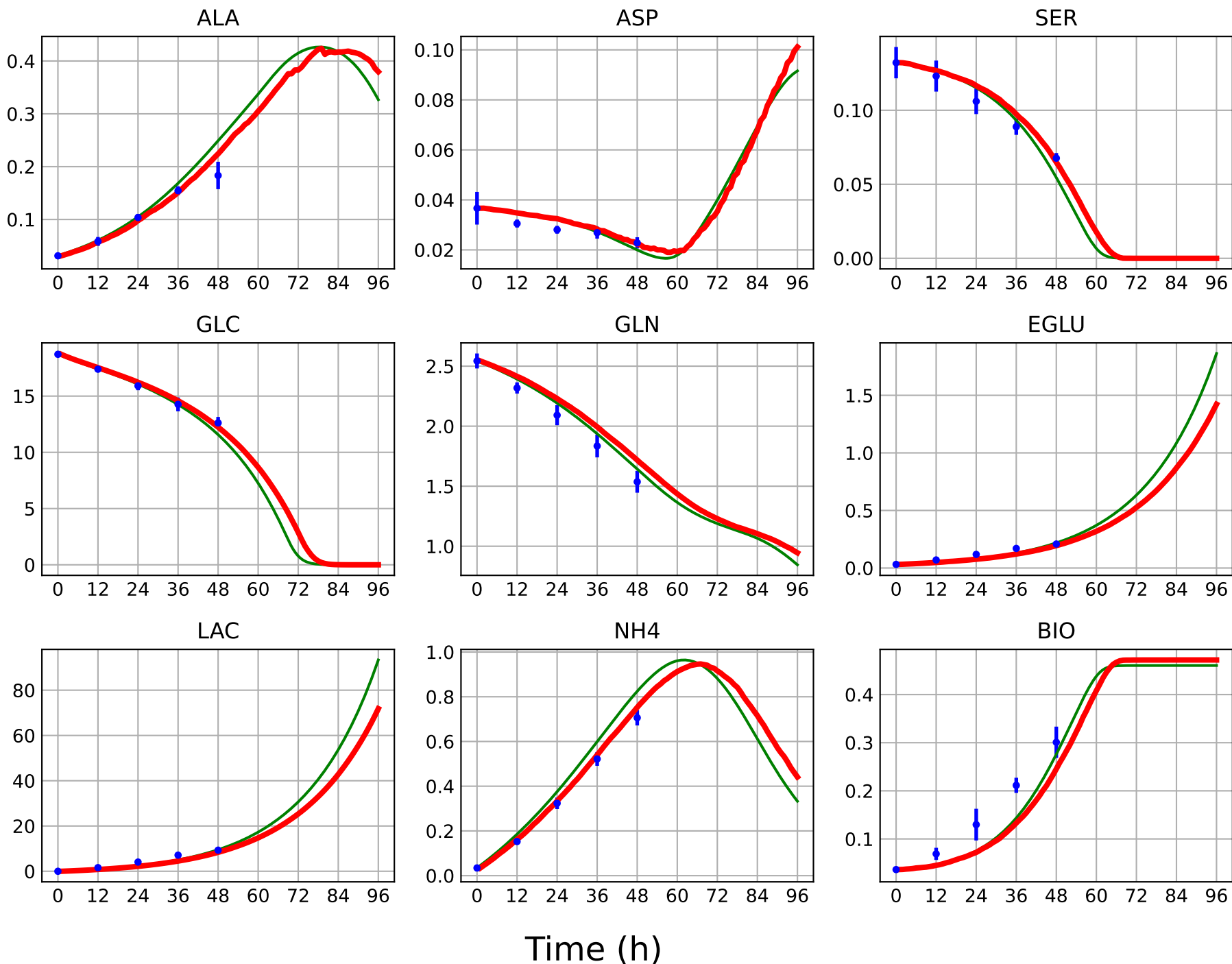

Supplement: Supplementary file 3 — Supplementary Software [file 42003_2023_5653_MOESM3_ESM.zip › MultiScaleModel-master/multi_scale_model/result/simulation-agg/HGLL-BIO.pdf]

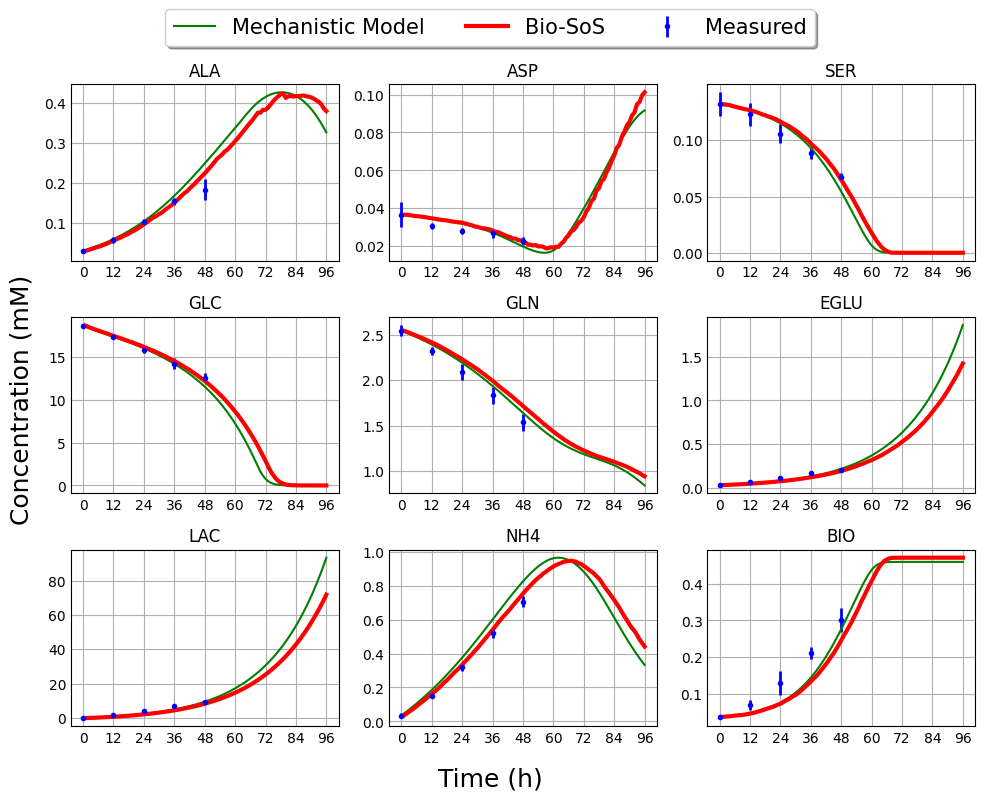

Supplement: Supplementary file 3 — Supplementary Software [file 42003_2023_5653_MOESM3_ESM.zip › MultiScaleModel-master/multi_scale_model/result/simulation-agg/HGLL-BIO.png]

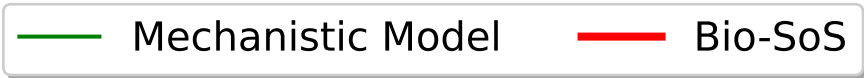

Concentration (mM)

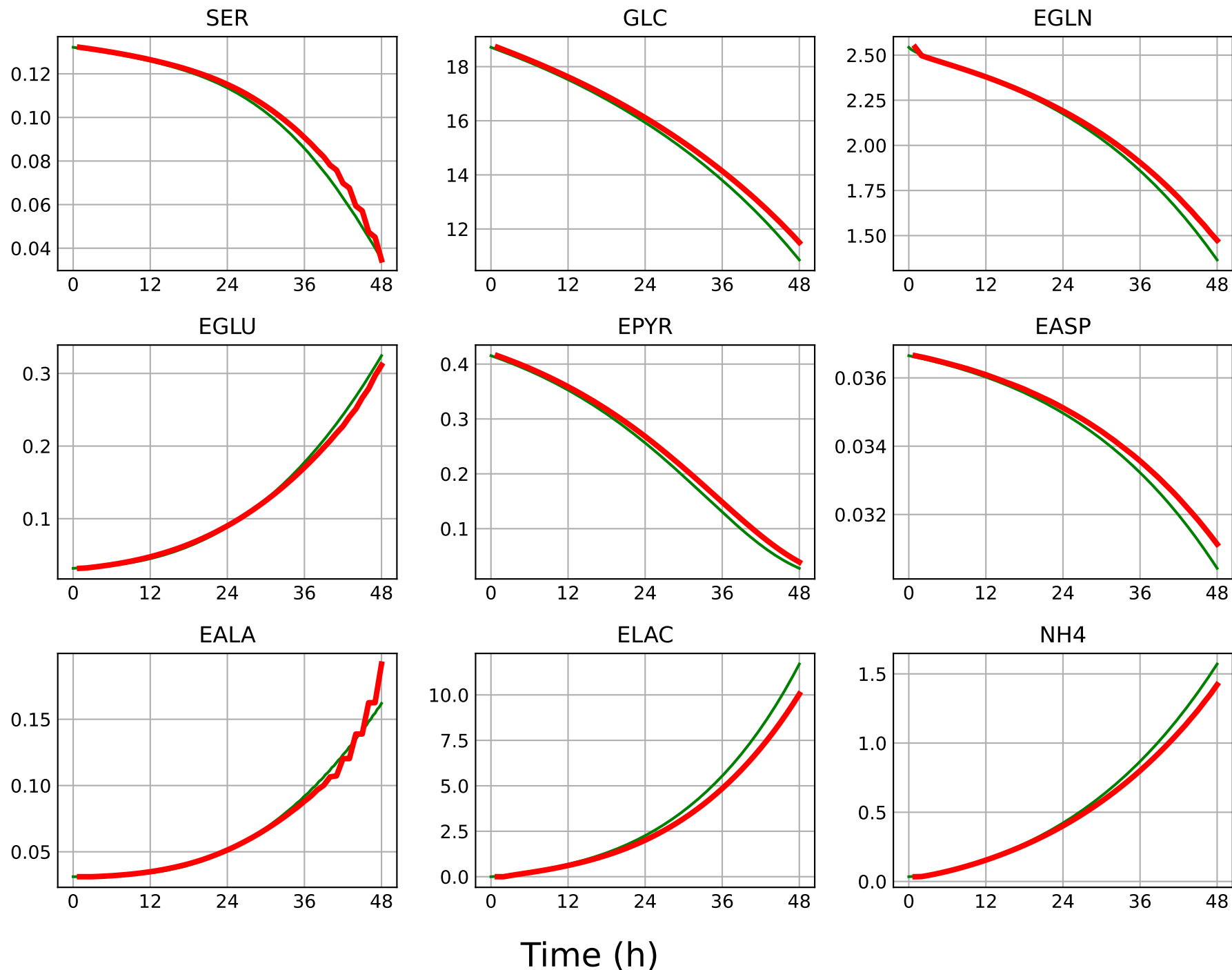

Supplement: Supplementary file 3 — Supplementary Software [file 42003_2023_5653_MOESM3_ESM.zip › MultiScaleModel-master/multi_scale_model/result/simulation-agg/HGLL-NH4-draw_measurement-False.pdf]

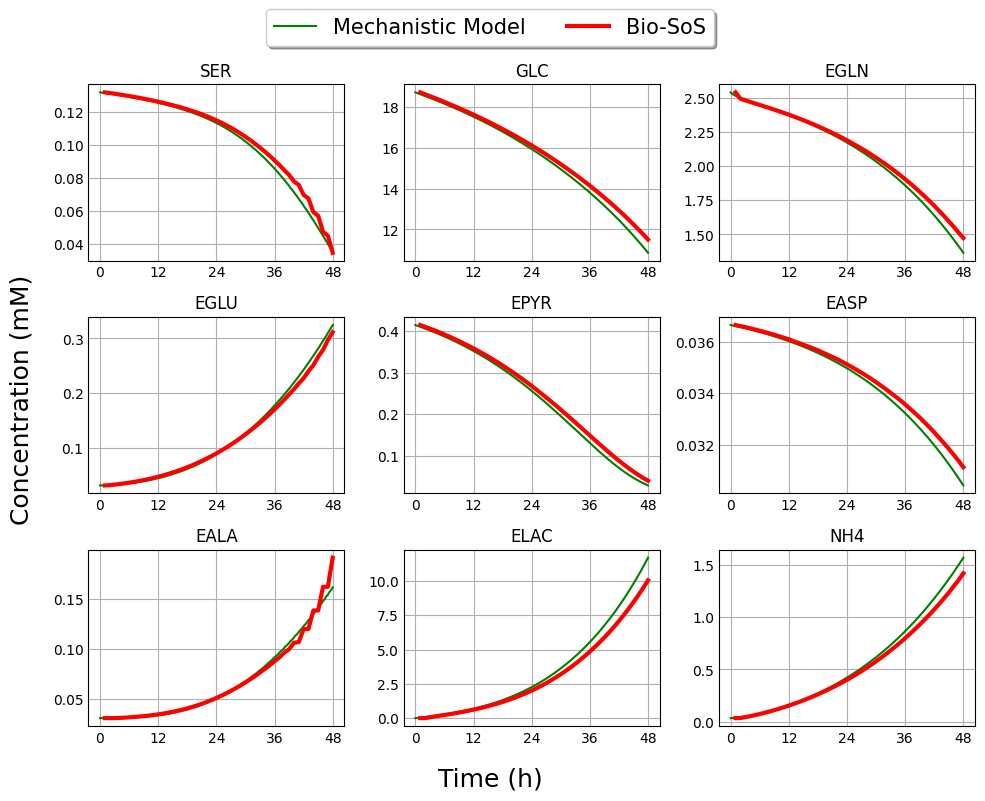

Supplement: Supplementary file 3 — Supplementary Software [file 42003_2023_5653_MOESM3_ESM.zip › MultiScaleModel-master/multi_scale_model/result/simulation-agg/HGLL-NH4-draw_measurement-False.png]

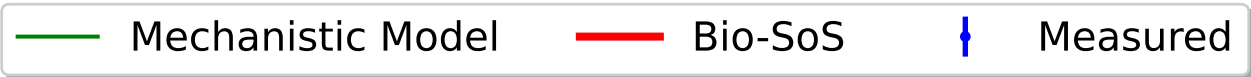

Concentration (mM)

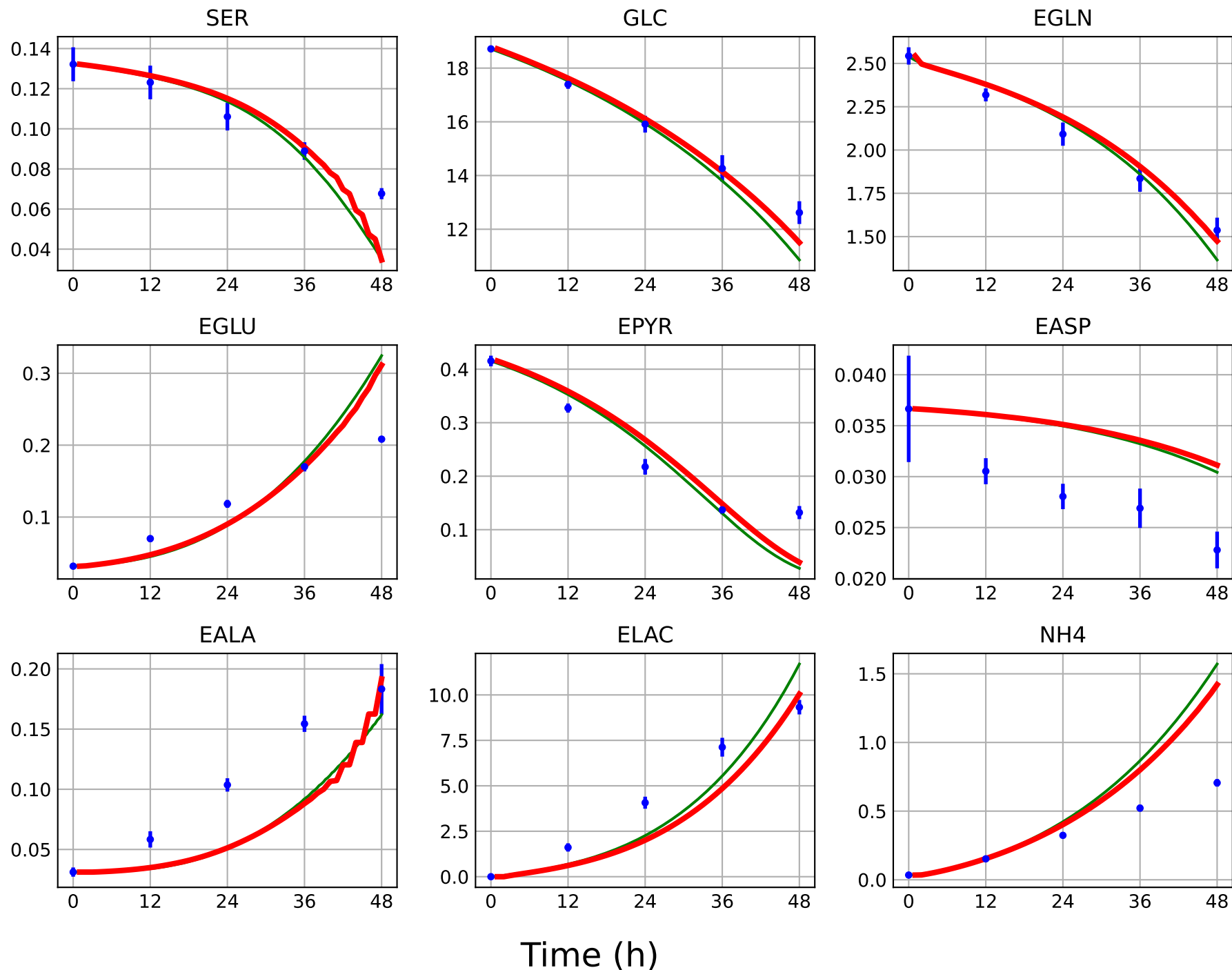

Supplement: Supplementary file 3 — Supplementary Software [file 42003_2023_5653_MOESM3_ESM.zip › MultiScaleModel-master/multi_scale_model/result/simulation-agg/HGLL-NH4-draw_measurement-True.pdf]

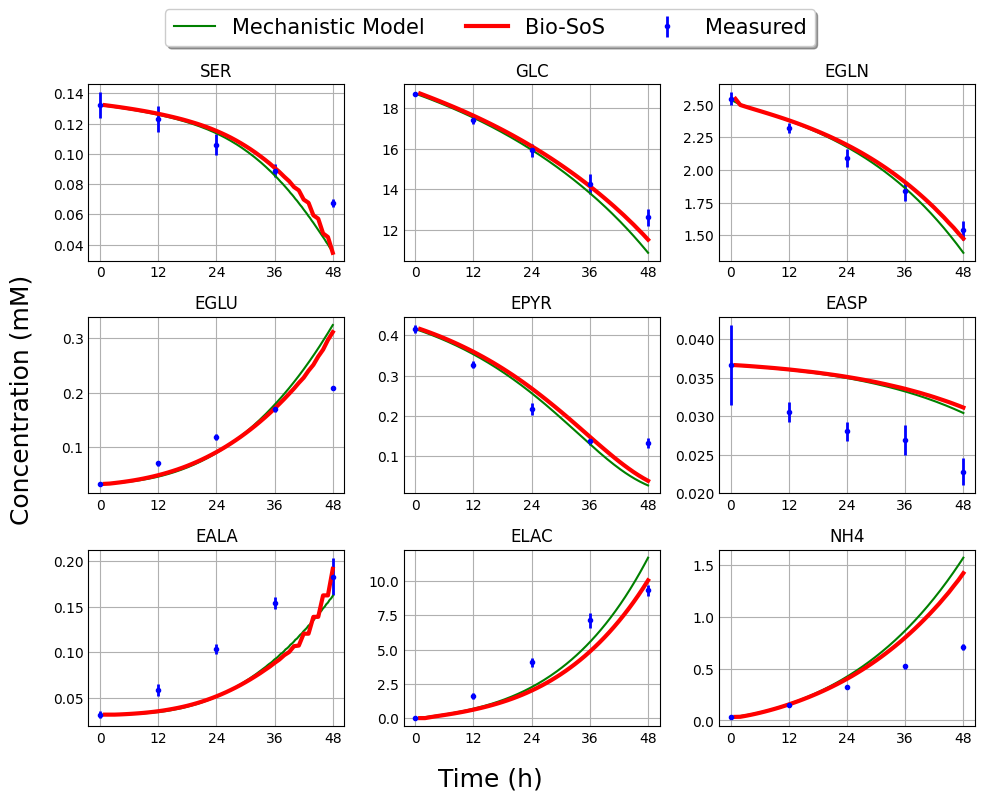

Supplement: Supplementary file 3 — Supplementary Software [file 42003_2023_5653_MOESM3_ESM.zip › MultiScaleModel-master/multi_scale_model/result/simulation-agg/HGLL-NH4-draw_measurement-True.png]

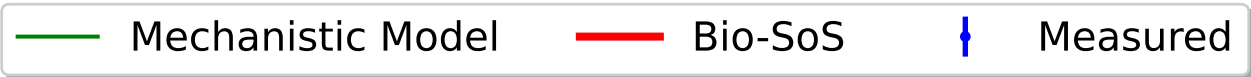

Concentration (mM)

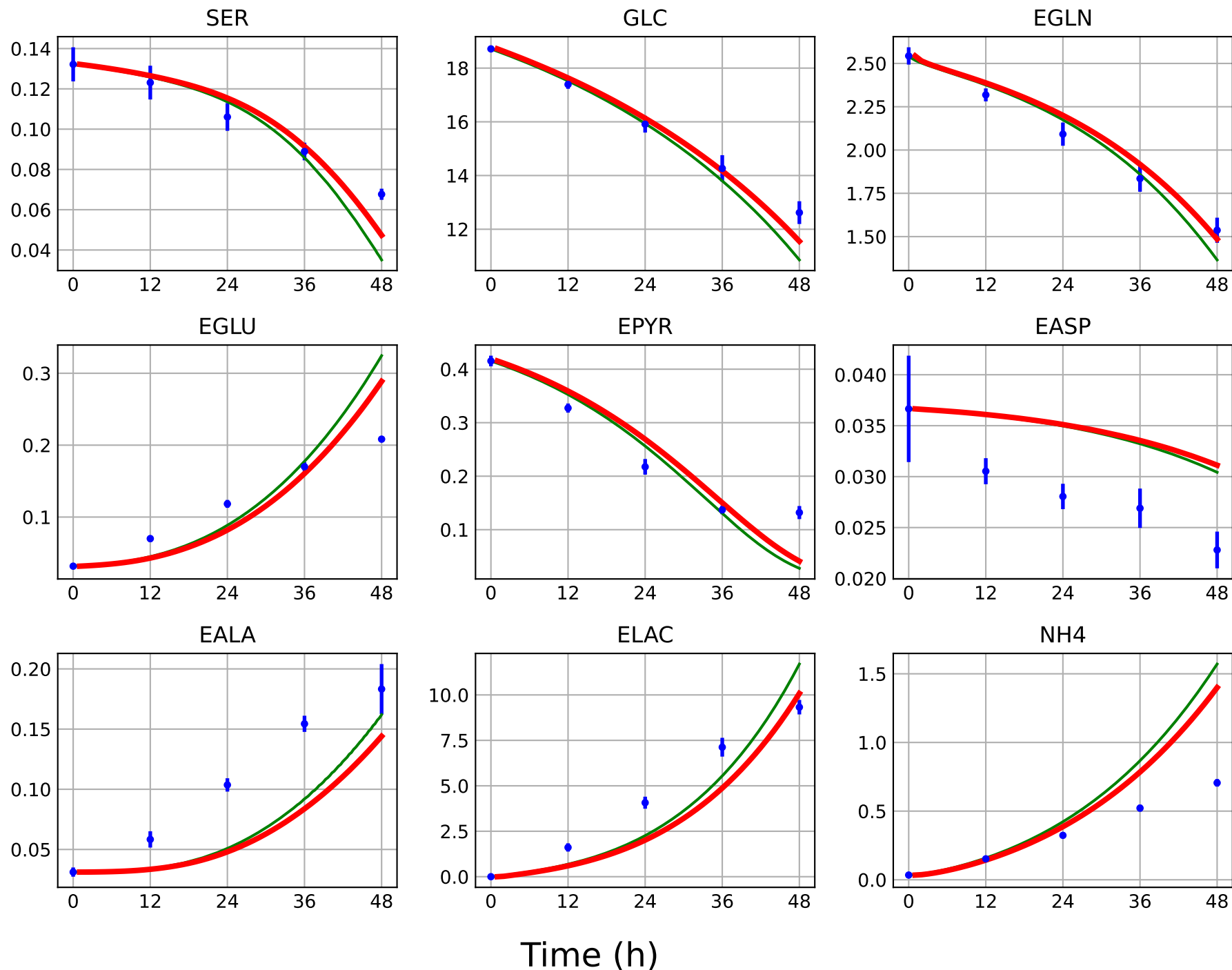

Supplement: Supplementary file 3 — Supplementary Software [file 42003_2023_5653_MOESM3_ESM.zip › MultiScaleModel-master/multi_scale_model/result/simulation-agg/HGLL-NH4.pdf]

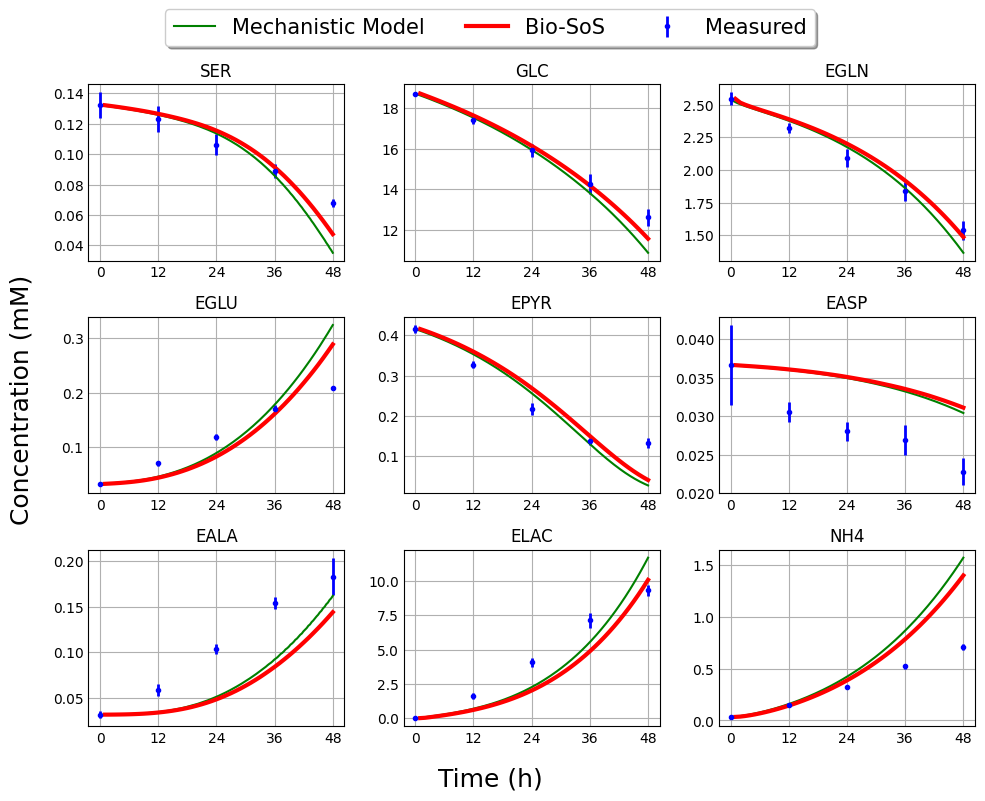

Supplement: Supplementary file 3 — Supplementary Software [file 42003_2023_5653_MOESM3_ESM.zip › MultiScaleModel-master/multi_scale_model/result/simulation-agg/HGLL-NH4.png]

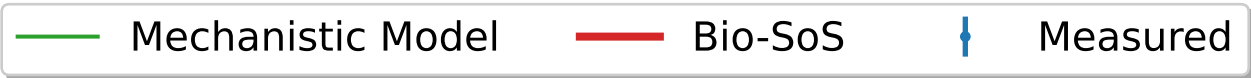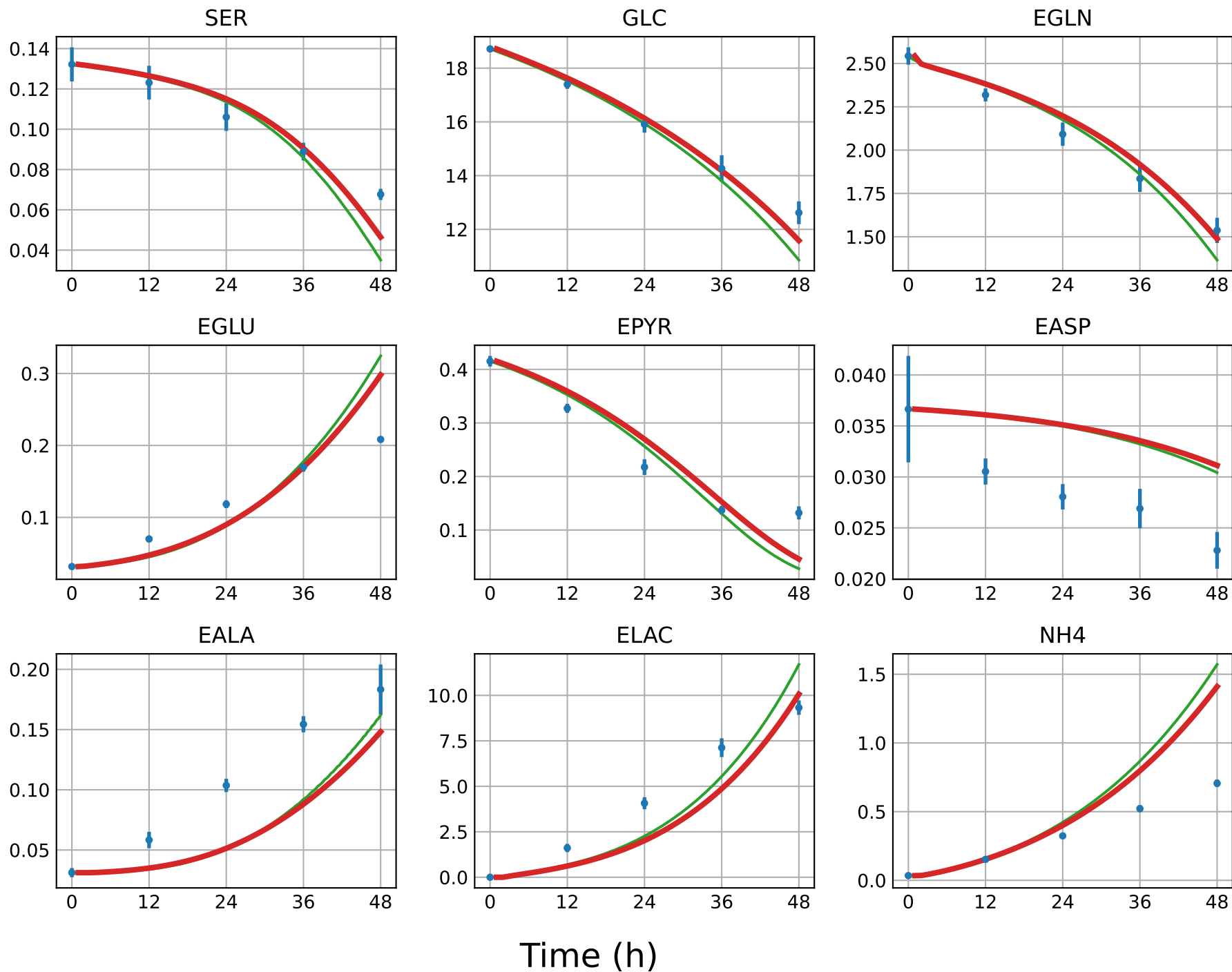

Supplement: Supplementary file 3 — Supplementary Software [file 42003_2023_5653_MOESM3_ESM.zip › MultiScaleModel-master/multi_scale_model/result/simulation-with-agg/HGLL-NH4-draw_measurement-True.pdf]

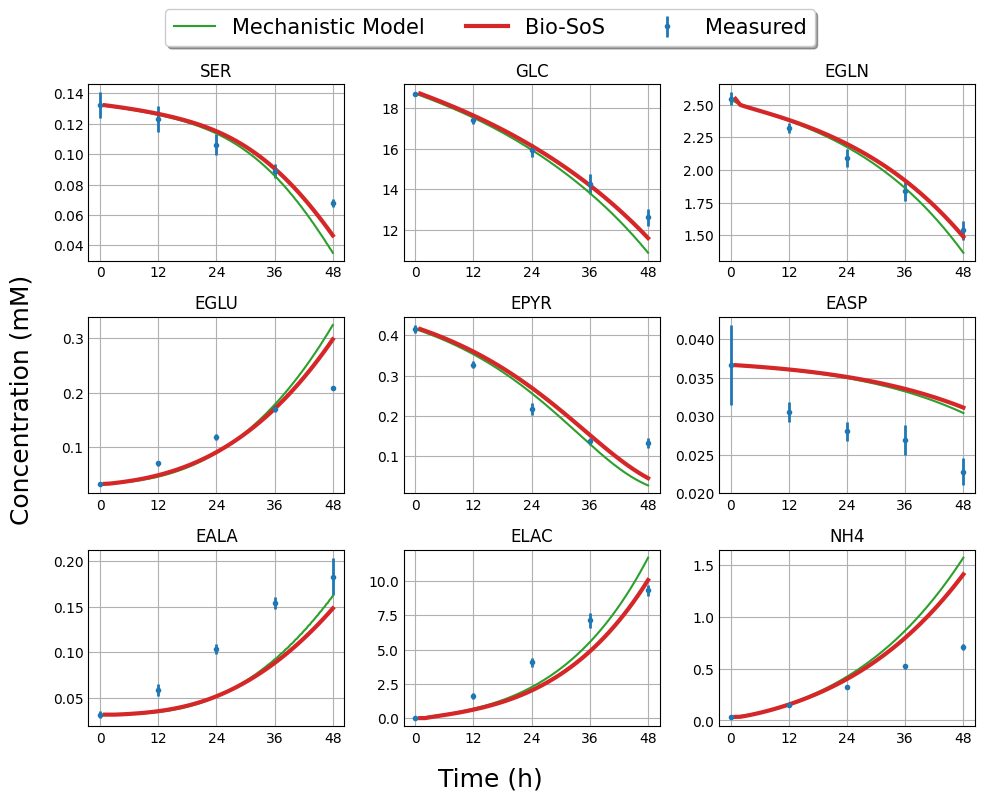

Supplement: Supplementary file 3 — Supplementary Software [file 42003_2023_5653_MOESM3_ESM.zip › MultiScaleModel-master/multi_scale_model/result/simulation-with-agg/HGLL-NH4-draw_measurement-True.png]

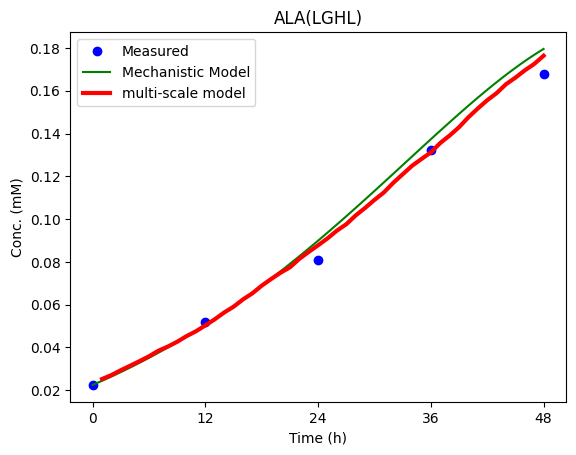

Supplement: Supplementary file 3 — Supplementary Software [file 42003_2023_5653_MOESM3_ESM.zip › MultiScaleModel-master/multi_scale_model/result/simulation/HGHL-28.png]

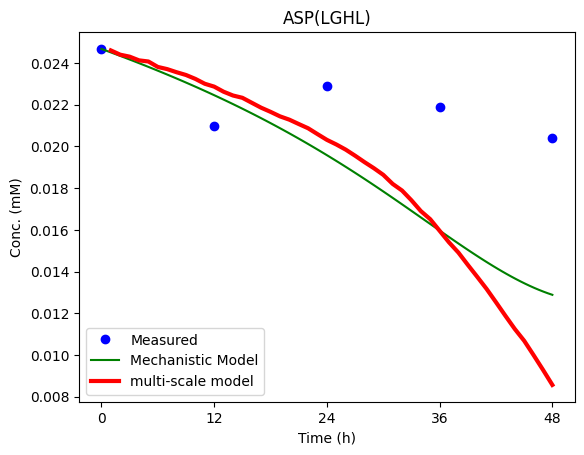

Supplement: Supplementary file 3 — Supplementary Software [file 42003_2023_5653_MOESM3_ESM.zip › MultiScaleModel-master/multi_scale_model/result/simulation/HGHL-29.png]

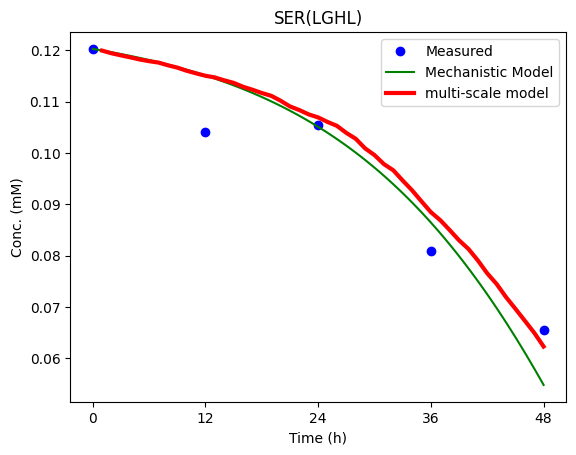

Supplement: Supplementary file 3 — Supplementary Software [file 42003_2023_5653_MOESM3_ESM.zip › MultiScaleModel-master/multi_scale_model/result/simulation/HGHL-36.png]

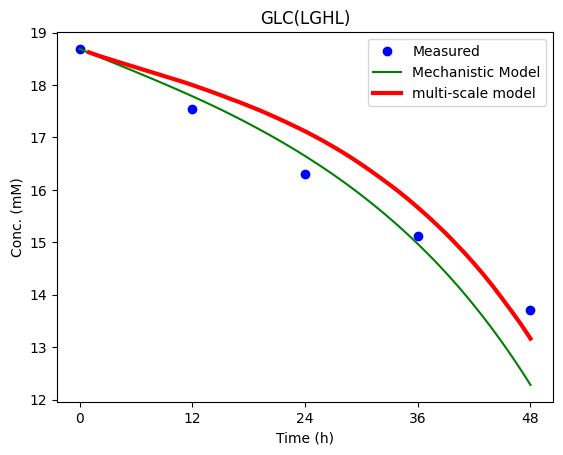

Supplement: Supplementary file 3 — Supplementary Software [file 42003_2023_5653_MOESM3_ESM.zip › MultiScaleModel-master/multi_scale_model/result/simulation/HGHL-39.png]

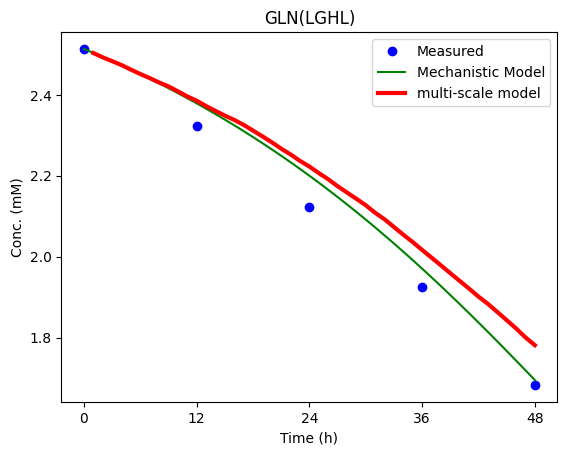

Supplement: Supplementary file 3 — Supplementary Software [file 42003_2023_5653_MOESM3_ESM.zip › MultiScaleModel-master/multi_scale_model/result/simulation/HGHL-40.png]

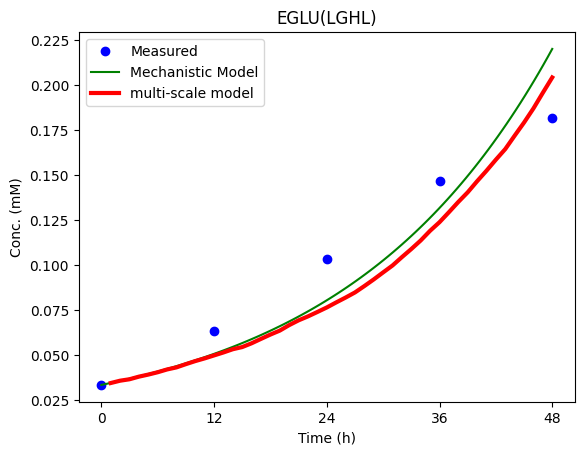

Supplement: Supplementary file 3 — Supplementary Software [file 42003_2023_5653_MOESM3_ESM.zip › MultiScaleModel-master/multi_scale_model/result/simulation/HGHL-41.png]

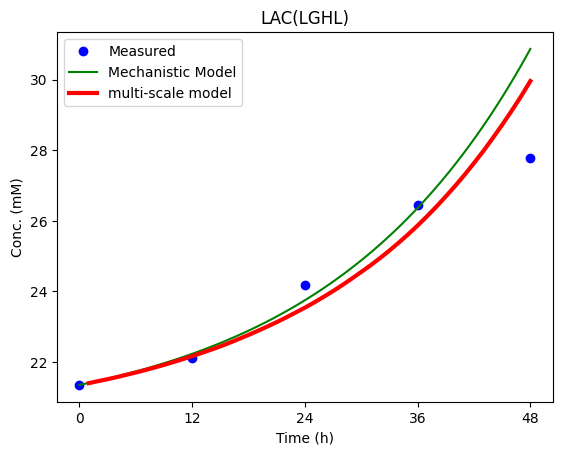

Supplement: Supplementary file 3 — Supplementary Software [file 42003_2023_5653_MOESM3_ESM.zip › MultiScaleModel-master/multi_scale_model/result/simulation/HGHL-42.png]

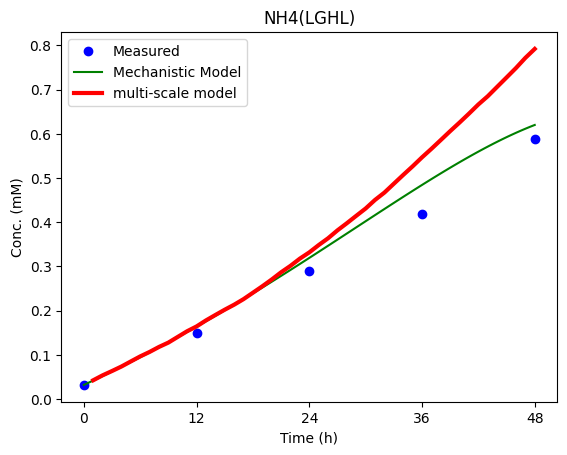

Supplement: Supplementary file 3 — Supplementary Software [file 42003_2023_5653_MOESM3_ESM.zip › MultiScaleModel-master/multi_scale_model/result/simulation/HGHL-43.png]

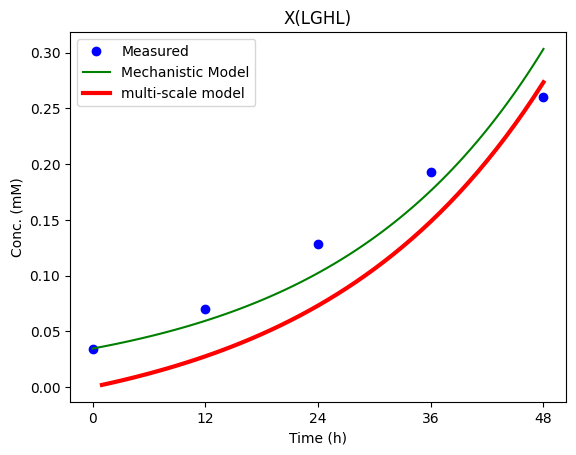

Supplement: Supplementary file 3 — Supplementary Software [file 42003_2023_5653_MOESM3_ESM.zip › MultiScaleModel-master/multi_scale_model/result/simulation/HGHL-45.png]

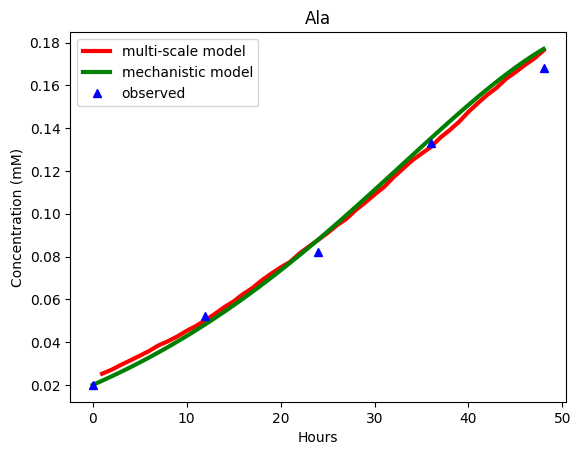

Supplement: Supplementary file 3 — Supplementary Software [file 42003_2023_5653_MOESM3_ESM.zip › MultiScaleModel-master/multi_scale_model/result/simulation/HGHL-Ala.png]

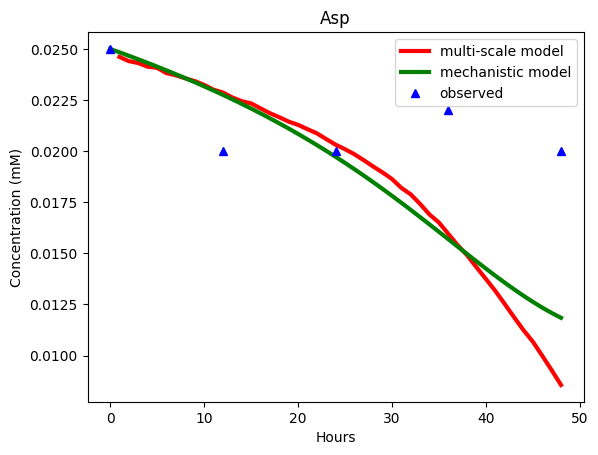

Supplement: Supplementary file 3 — Supplementary Software [file 42003_2023_5653_MOESM3_ESM.zip › MultiScaleModel-master/multi_scale_model/result/simulation/HGHL-Asp.png]

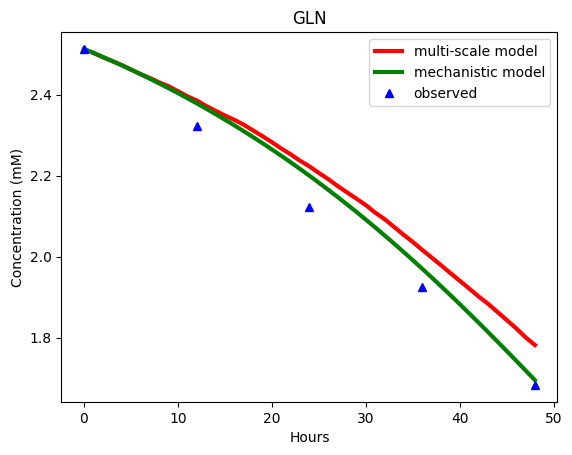

Supplement: Supplementary file 3 — Supplementary Software [file 42003_2023_5653_MOESM3_ESM.zip › MultiScaleModel-master/multi_scale_model/result/simulation/HGHL-GLN.png]
