# Supplementary material for: Stochastic biological system-of-systems modelling for iPSC culture
Source: Commun Biol. 2024 Jan 8;7:39. doi: 10.1038/s42003-023-05653-w (PMC10774284; doi:10.1038/s42003-023-05653-w)
Supplement: Supplementary file 4 — Reporting Summary [file 42003_2023_5653_MOESM4_ESM.pdf]

nature portfolio

Corresponding author(s): Wei Xie, Sarah W. Harcum

Last updated by author(s): Nov 23, 2023

Reporting Summary

Nature Portfolio wishes to improve the reproducibility of the work that we publish. This form provides structure for consistency and transparency in reporting. For further information on Nature Portfolio policies, see our Editorial Policies and the Editorial Policy Checklist.

Statistics

For all statistical analyses, confirm that the following items are present in the figure legend, table legend, main text, or Methods section.

n/a	Confirmed	
☐	☒	The exact sample size (n) for each experimental group/condition, given as a discrete number and unit of measurement
☐	☒	A statement on whether measurements were taken from distinct samples or whether the same sample was measured repeatedly
☒	☐	The statistical test(s) used AND whether they are one- or two-sided<br>*Only common tests should be described solely by name; describe more complex techniques in the Methods section.*
☒	☐	A description of all covariates tested
☒	☐	A description of any assumptions or corrections, such as tests of normality and adjustment for multiple comparisons
☐	☒	A full description of the statistical parameters including central tendency (e.g. means) or other basic estimates (e.g. regression coefficient) AND variation (e.g. standard deviation) or associated estimates of uncertainty (e.g. confidence intervals)
☒	☐	For null hypothesis testing, the test statistic (e.g. F, t, r) with confidence intervals, effect sizes, degrees of freedom and P value noted<br>*Give P values as exact values whenever suitable.*
☒	☐	For Bayesian analysis, information on the choice of priors and Markov chain Monte Carlo settings
☒	☐	For hierarchical and complex designs, identification of the appropriate level for tests and full reporting of outcomes
☒	☐	Estimates of effect sizes (e.g. Cohen's d, Pearson's r), indicating how they were calculated

Our web collection on statistics for biologists contains articles on many of the points above.

Software and code

Policy information about availability of computer code

Data collection	The data used in this work are collected from open-access databases. The monolayer iPSC cell culture data are in Supplementary Data [11]. The bioreactor iPSC culture data are from Kwok et al. [7]. The parameters of population balance model are adapted from Wu et al. [14]. The diffusion coefficients are from multiple published sources [49, 50, 51, 52, 53, 54, 55].
Data analysis	The simulation and data analyses were implemented using Python computer language and open-sourced libraries including Numpy, Pandas, Matplotlib and Scipy. All codes have been packaged. The code generating the main results in this work is available at GitHub repository (https://github.com/zhenghuazx/MultiScaleModel). The computer simulation and analysis have been made available to public so that all results and plots can be easily reproduced by running the commands described in README.md file.

For manuscripts utilizing custom algorithms or software that are central to the research but not yet described in published literature, software must be made available to editors and reviewers. We strongly encourage code deposition in a community repository (e.g. GitHub). See the Nature Portfolio guidelines for submitting code & software for further information.

Data

Policy information about availability of data

All manuscripts must include a data availability statement. This statement should provide the following information, where applicable:

- Accession codes, unique identifiers, or web links for publicly available datasets
- A description of any restrictions on data availability
- For clinical datasets or third party data, please ensure that the statement adheres to our policy

The data used in this work are collected from open-access databases. The monolayer iPSC cell culture data are in Supplementary Data [11]. The bioreactor iPSC culture data are from Kwok et al. [7]. The parameters of population balance model are adapted from Wu et al. [14]. The diffusion coefficients are from multiple published sources [49, 50, 51, 52, 53, 54, 55].

Research involving human participants, their data, or biological material

Policy information about studies with human participants or human data. See also policy information about sex, gender (identity/presentation), and sexual orientation and race, ethnicity and racism.

Reporting on sex and gender	Not applied
Reporting on race, ethnicity, or other socially relevant groupings	Not applied
Population characteristics	Not applied
Recruitment	Not applied
Ethics oversight	Not applied

Note that full information on the approval of the study protocol must also be provided in the manuscript.

Field-specific reporting

Please select the one below that is the best fit for your research. If you are not sure, read the appropriate sections before making your selection.

☒ Life sciences ☐ Behavioural & social sciences ☐ Ecological, evolutionary & environmental sciences

For a reference copy of the document with all sections, see nature.com/documents/nr-reporting-summary-flat.pdf

Life sciences study design

All studies must disclose on these points even when the disclosure is negative.

Sample size	Experiment data are adapted from open-source databases or literatures with samples sizes determined in the corresponding experiments.
Data exclusions	No data are excluded from the analyses.
Replication	The reproductivity of the results was verified by running the simulations multiple times. The simple command lines have been provided to guarantee the reproduction of the result in this paper.
Randomization	Not applied
Blinding	Not applied

Reporting for specific materials, systems and methods

We require information from authors about some types of materials, experimental systems and methods used in many studies. Here, indicate whether each material, system or method listed is relevant to your study. If you are not sure if a list item applies to your research, read the appropriate section before selecting a response.

Materials & experimental systems

n/a	Involved in the study
☒	☐ Antibodies
☒	☐ Eukaryotic cell lines
☒	☐ Palaeontology and archaeology
☒	☐ Animals and other organisms
☒	☐ Clinical data
☒	☐ Dual use research of concern
☒	☐ Plants

Methods

n/a	Involved in the study
☒	☐ ChIP-seq
☒	☐ Flow cytometry
☒	☐ MRI-based neuroimaging
