# Supplementary material for: Stochastic biological system-of-systems modelling for iPSC culture
Source: Commun Biol. 2024 Jan 8;7:39. doi: 10.1038/s42003-023-05653-w (PMC10774284; doi:10.1038/s42003-023-05653-w)
Supplement: Supplementary file 3 — Supplementary Software [file Biomass-metabolites-ala-0.15.pdf › MultiScaleModel-master/multi_scale_model/result/optimal_size/Biomass-metabolites-ala-0.15.pdf]

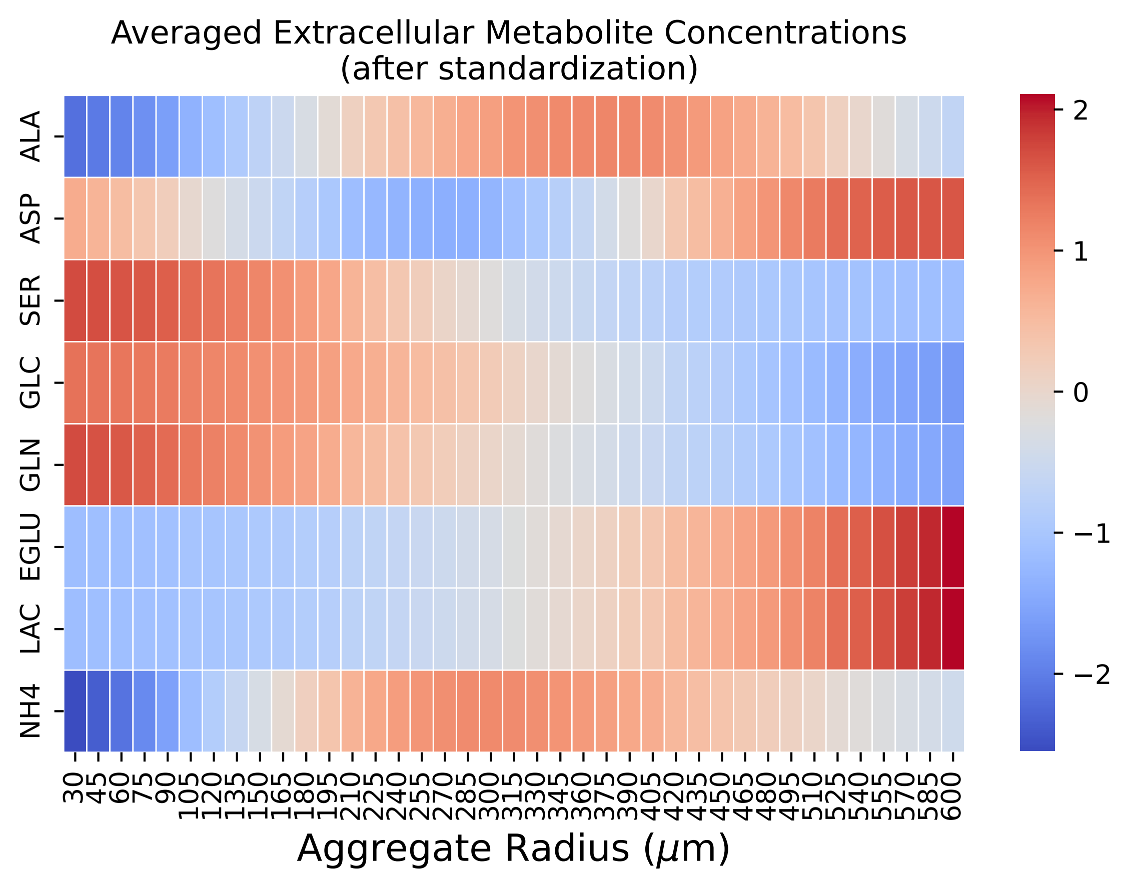

Averaged Extracellular Metabolite Concentrations
(after standardization)
ALA
ASP
SER
GLC
GLN
EGLU
LAC
NH4
30
45
60
75
90
105
120
135
150
165
180
195
210
225
240
255
270
285
300
315
330
345
360
375
390
405
420
435
450
465
480
495
510
525
540
555
570
585
600
Aggregate Radius (μm)
2
1
0
−1
−2
