# Supplementary material for: Stochastic biological system-of-systems modelling for iPSC culture
Source: Commun Biol. 2024 Jan 8;7:39. doi: 10.1038/s42003-023-05653-w (PMC10774284; doi:10.1038/s42003-023-05653-w)
Supplement: Supplementary file 3 — Supplementary Software [file 42003_2023_5653_MOESM3_ESM.zip › MultiScaleModel-master/multi_scale_model/result/variance-component-analysis/mean-variance-24.pdf]

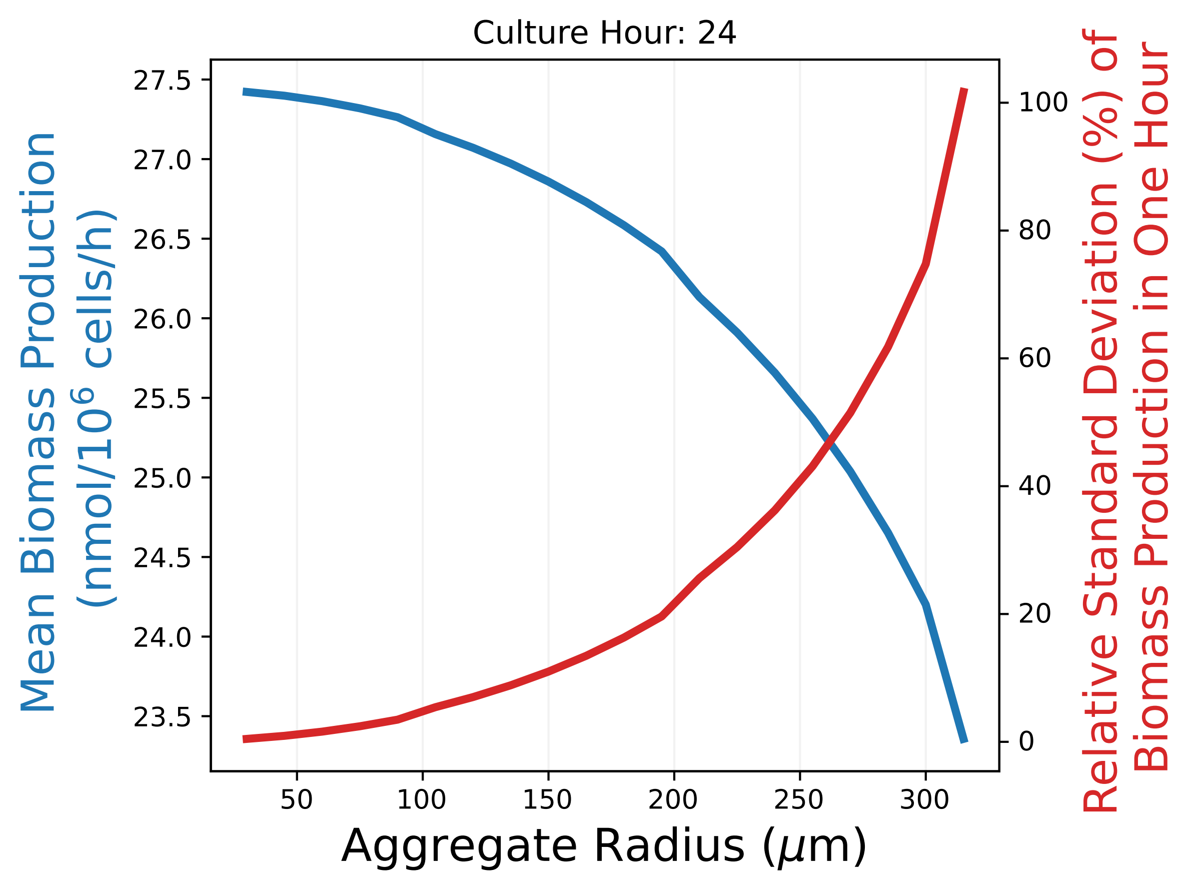

Culture Hour: 24
Mean Biomass Production (nmol/10^6 cells/h)
27.5
27.0
26.5
26.0
25.5
25.0
24.5
24.0
23.5
Relative Standard Deviation (%) of Biomass Production in One Hour
100
80
60
40
20
0
50
100
150
200
250
300
Aggregate Radius (μm)
