# Supplementary material for: Stochastic biological system-of-systems modelling for iPSC culture
Source: Commun Biol. 2024 Jan 8;7:39. doi: 10.1038/s42003-023-05653-w (PMC10774284; doi:10.1038/s42003-023-05653-w)
Supplement: Supplementary file 3 — Supplementary Software [file 42003_2023_5653_MOESM3_ESM.zip › MultiScaleModel-master/multi_scale_model/result/variance-component-analysis/mean-variance-57.pdf]

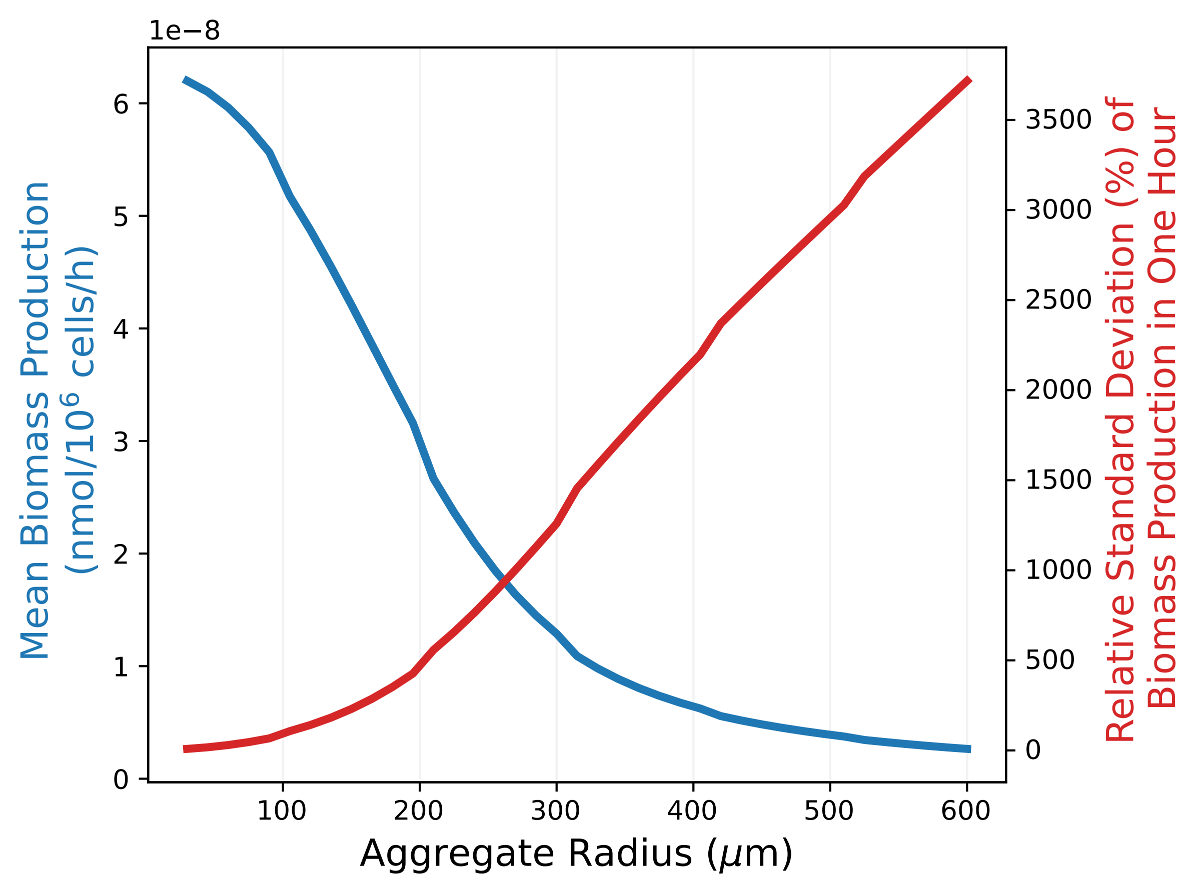
1e−8
Mean Biomass Production (nmol/10^6 cells/h)
0
1
2
3
4
5
6
100
200
300
400
500
600
Aggregate Radius (μm)
0
500
1000
1500
2000
2500
3000
3500
Relative Standard Deviation (%) of Biomass Production in One Hour
