# Supplementary material for: Stochastic biological system-of-systems modelling for iPSC culture
Source: Commun Biol. 2024 Jan 8;7:39. doi: 10.1038/s42003-023-05653-w (PMC10774284; doi:10.1038/s42003-023-05653-w)
Supplement: Supplementary file 3 — Supplementary Software [file 42003_2023_5653_MOESM3_ESM.zip › MultiScaleModel-master/multi_scale_model/result/variance-component-analysis/mean-variance-58.pdf]

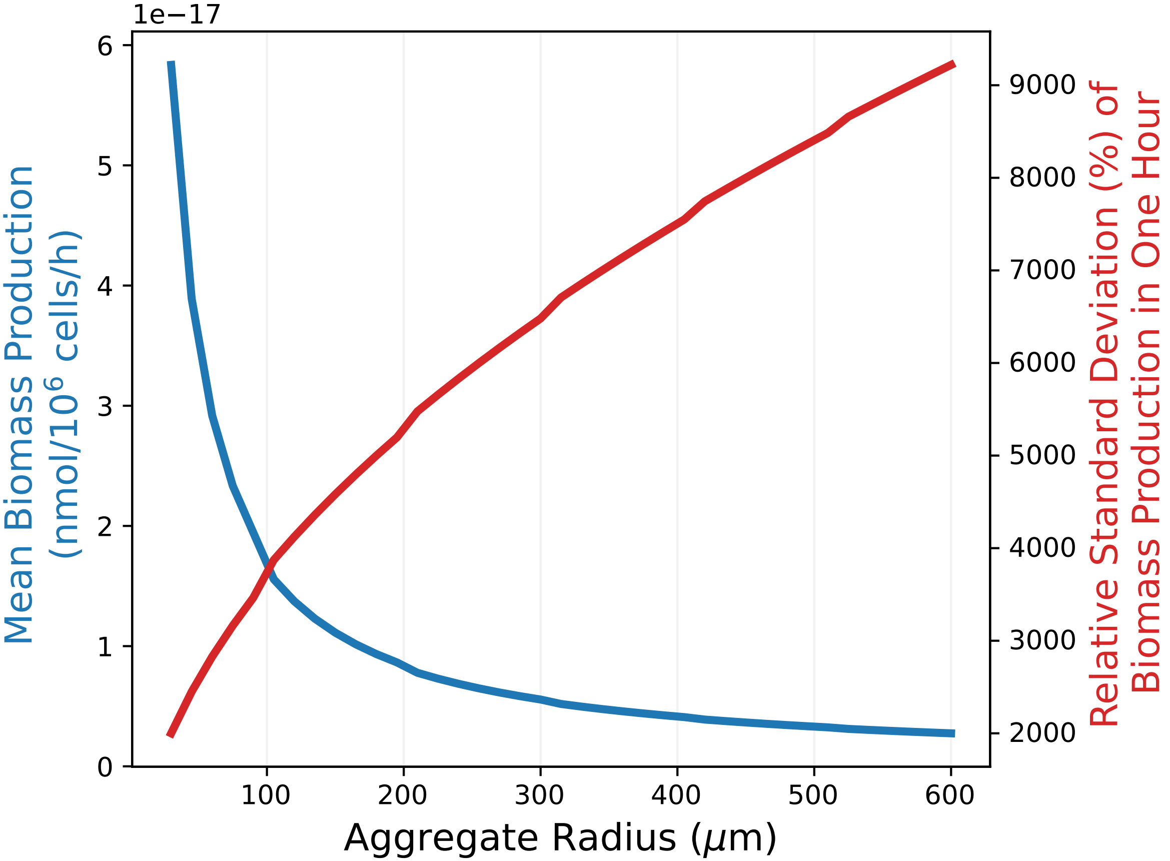
1e−17
6
5
4
3
2
1
0
100
200
300
400
500
600
Aggregate Radius (μm)
Mean Biomass Production (nmol/10⁶ cells/h)
9000
8000
7000
6000
5000
4000
3000
2000
Relative Standard Deviation (%) of Biomass Production in One Hour
