# Supplementary material for: Stochastic biological system-of-systems modelling for iPSC culture
Source: Commun Biol. 2024 Jan 8;7:39. doi: 10.1038/s42003-023-05653-w (PMC10774284; doi:10.1038/s42003-023-05653-w)
Supplement: Supplementary file 3 — Supplementary Software [file 42003_2023_5653_MOESM3_ESM.zip › MultiScaleModel-master/multi_scale_model/result/variance-component-analysis/mean-variance-72.pdf]

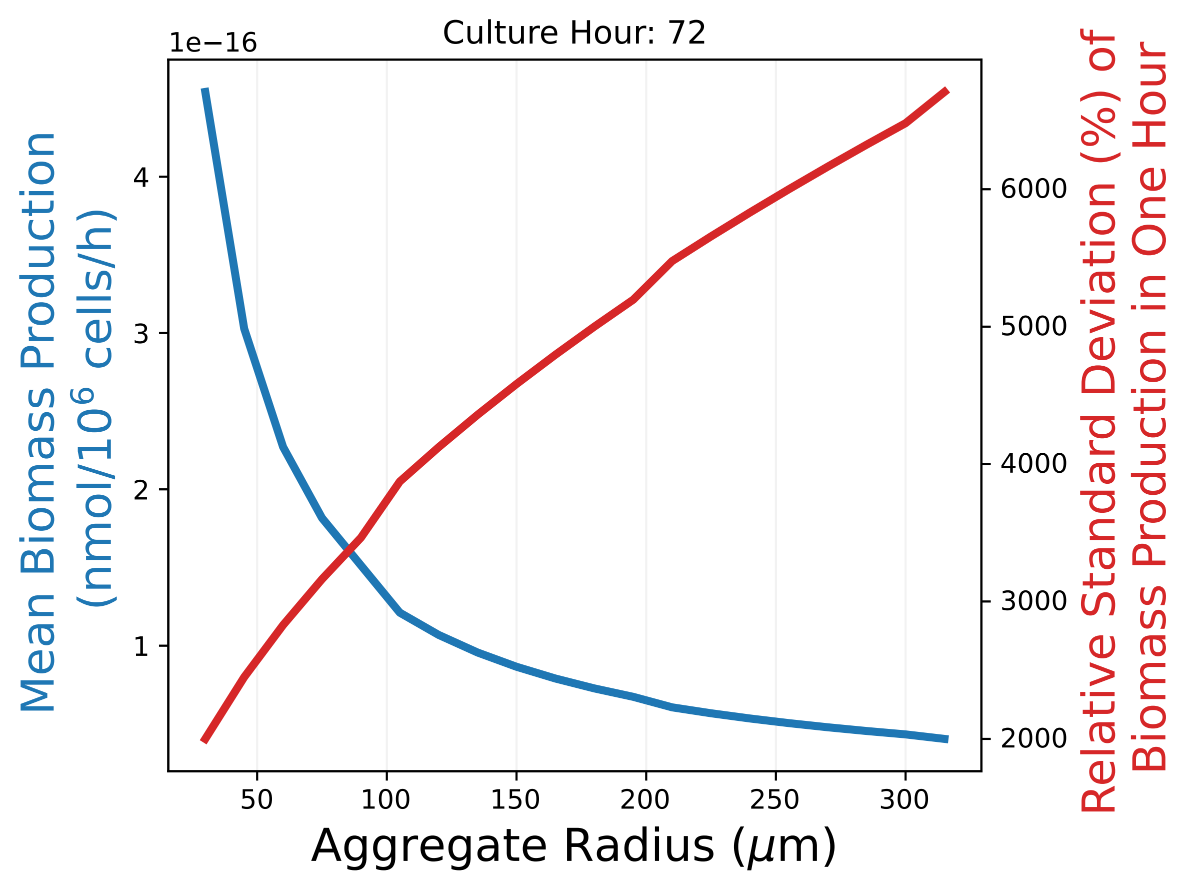

Culture Hour: 72
1e−16
Mean Biomass Production
(nmol/10^6 cells/h)
Relative Standard Deviation (%) of
Biomass Production in One Hour
Aggregate Radius (μm)
1
2
3
4
2000
3000
4000
5000
6000
50
100
150
200
250
300
