# Supplementary material for: Stochastic biological system-of-systems modelling for iPSC culture
Source: Commun Biol. 2024 Jan 8;7:39. doi: 10.1038/s42003-023-05653-w (PMC10774284; doi:10.1038/s42003-023-05653-w)
Supplement: Supplementary file 3 — Supplementary Software [file 42003_2023_5653_MOESM3_ESM.zip › MultiScaleModel-master/multi_scale_model/result/variance-component-analysis/rsd-with-time.pdf]

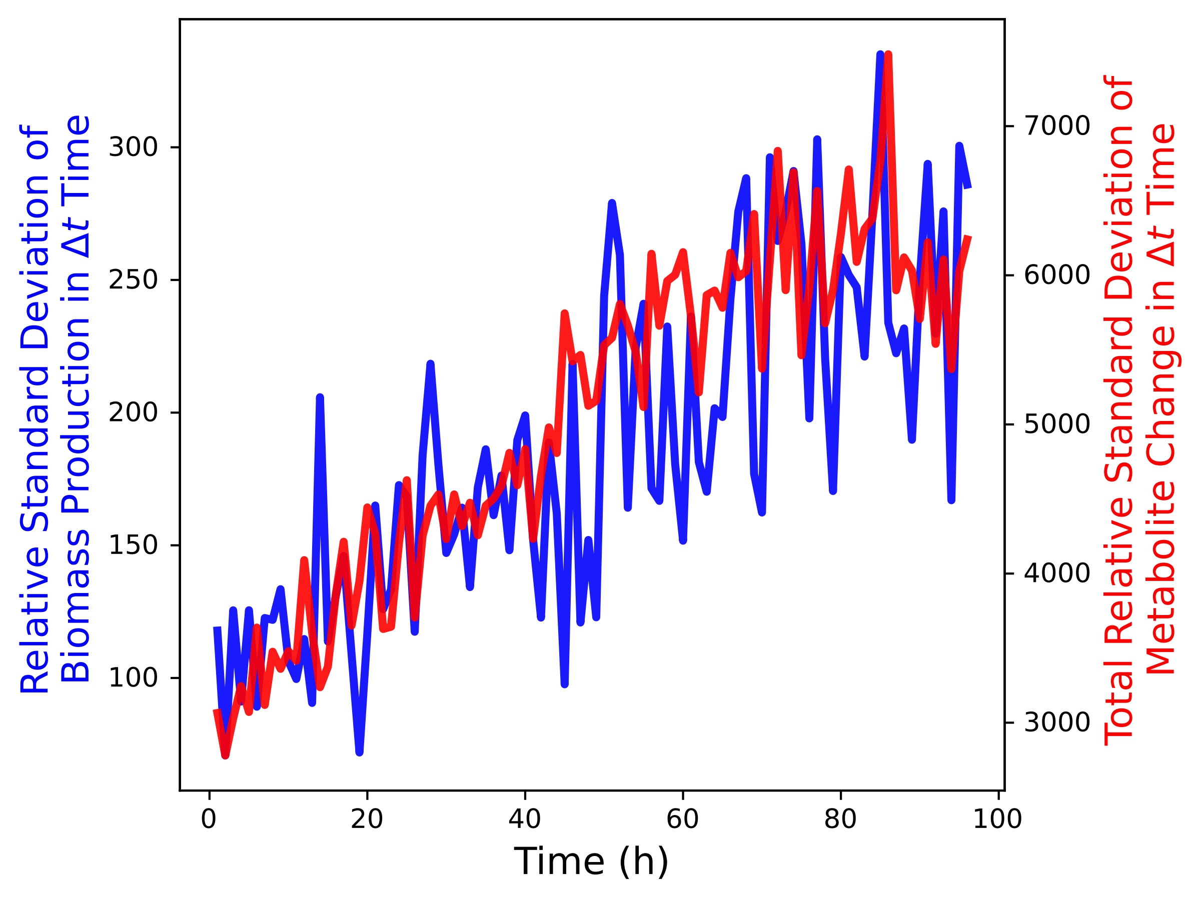

Relative Standard Deviation of
Biomass Production in Δt Time
300
250
200
150
100
Total Relative Standard Deviation of
Metabolite Change in Δt Time
7000
6000
5000
4000
3000
0
20
40
60
80
100
Time (h)
