# Supplementary material for: Stochastic biological system-of-systems modelling for iPSC culture
Source: Commun Biol. 2024 Jan 8;7:39. doi: 10.1038/s42003-023-05653-w (PMC10774284; doi:10.1038/s42003-023-05653-w)
Supplement: Supplementary file 3 — Supplementary Software [file 42003_2023_5653_MOESM3_ESM.zip › MultiScaleModel-master/multi_scale_model/result/variance-component-analysis/variance-decomposition-with-time.pdf]

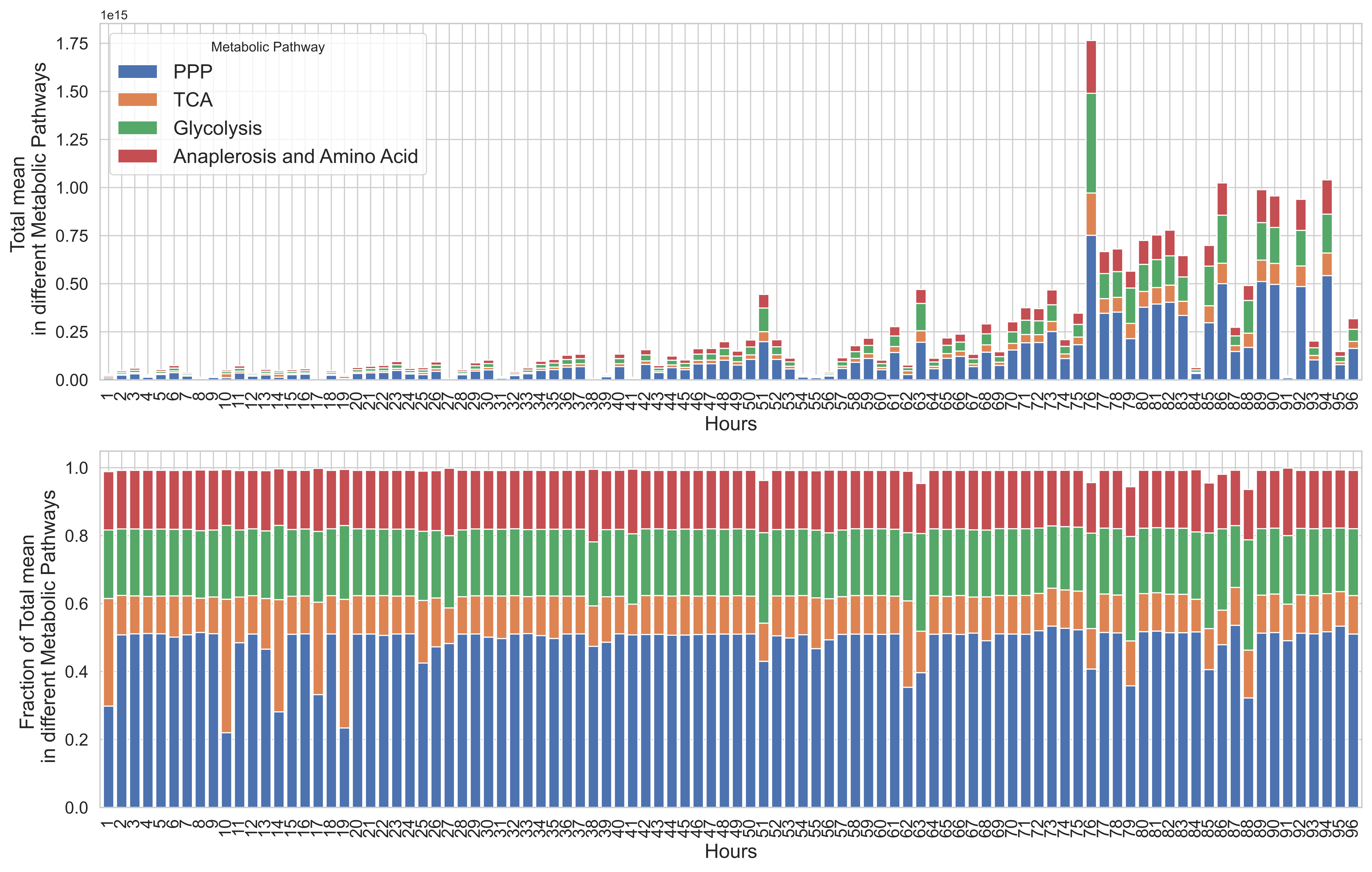

1e15
Total mean
in different Metabolic Pathways
Metabolic Pathway
PPP
TCA
Glycolysis
Anaplerosis and Amino Acid
Hours
Fraction of Total mean
in different Metabolic Pathways
Hours
